# A Conserved Second Sphere Residue Tunes Copper Site Reactivity in Lytic Polysaccharide Monooxygenases

**DOI:** 10.1021/jacs.3c05342

**Published:** 2023-08-16

**Authors:** Kelsi R. Hall, Chris Joseph, Iván Ayuso-Fernández, Ashish Tamhankar, Lukas Rieder, Rannei Skaali, Ole Golten, Frank Neese, Åsmund K. Røhr, Sergio A. V. Jannuzzi, Serena DeBeer, Vincent G. H. Eijsink, Morten Sørlie

**Affiliations:** † Faculty of Chemistry, Biotechnology and Food Science, Norwegian University of Life Sciences (NMBU), 1432, Ås, Norway; ‡ Max Planck Institute for Chemical Energy Conversion, Stiftstraße 34-36, 45470 Mülheim an der Ruhr, Germany; § Institute for Molecular Biotechnology, Graz University of Technology, 8010, Graz, Austria; ∥ Max-Planck-Institut für Kohlenforschung, Kaiser-Wilhelm-Platz 1, 45470 Mülheim an der Ruhr, Germany

## Abstract

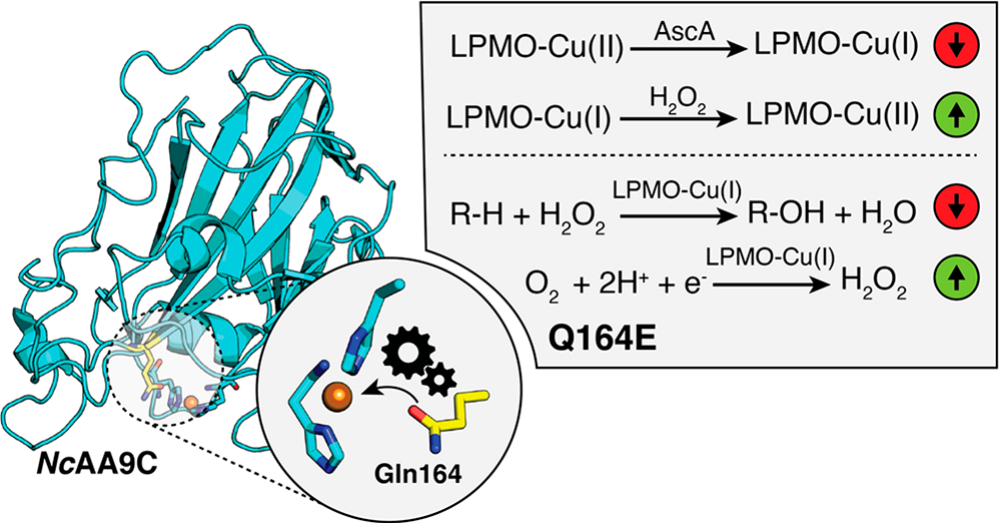

Lytic polysaccharide monooxygenases (LPMOs) are powerful monocopper enzymes that can activate strong C–H bonds through a mechanism that remains largely unknown. Herein, we investigated the role of a conserved glutamine/glutamate in the second coordination sphere. Mutation of the Gln in *Nc*AA9C to Glu, Asp, or Asn showed that the nature and distance of the headgroup to the copper fine-tune LPMO functionality and copper reactivity. The presence of Glu or Asp close to the copper lowered the reduction potential and decreased the ratio between the reduction and reoxidation rates by up to 500-fold. All mutants showed increased enzyme inactivation, likely due to changes in the confinement of radical intermediates, and displayed changes in a protective hole-hopping pathway. Electron paramagnetic resonance (EPR) and X-ray absorption spectroscopic (XAS) studies gave virtually identical results for all *Nc*AA9C variants, showing that the mutations do not directly perturb the Cu(II) ligand field. DFT calculations indicated that the higher experimental reoxidation rate observed for the Glu mutant could be reconciled if this residue is protonated. Further, for the glutamic acid form, we identified a Cu(III)-hydroxide species formed in a single step on the H_2_O_2_ splitting path. This is in contrast to the Cu(II)-hydroxide and hydroxyl intermediates, which are predicted for the WT and the unprotonated glutamate variant. These results show that this second sphere residue is a crucial determinant of the catalytic functioning of the copper-binding histidine brace and provide insights that may help in understanding LPMOs and LPMO-inspired synthetic catalysts.

## Introduction

Lytic polysaccharide monooxygenases (LPMOs) are monocopper enzymes able to cleave glycosidic bonds in a range of polysaccharides including chitin^1^ and cellulose.^2−4^ Activation of C–H bonds at the C1 or the C4 of the scissile glycosidic bonds in these crystalline polysaccharides requires powerful redox chemistry to overcome an activation barrier on the order of 95 kcal/mol.^5,6^ Understanding how these enzymes can catalyze such reactions would provide insights that can enable the design of improved LPMOs or synthetic Cu-catalysts capable of activating similarly strong C–H bonds.^7−9^ The copper environment in such synthetic catalysts can be manipulated through placement of particular functional groups in the secondary coordination sphere.^10^ Therefore, understanding how copper site reactivity is modulated by the local LPMO environment may provide hints about how synthetic copper sites could be optimized through rational design. Bridging the gap between enzymatic and synthetic catalysts may eventually expand the range of substrates for which efficient C–H bond activation is attainable and help synthetic catalysts to achieve higher turnovers numbers which are evident in natural LPMOs,^11−13^ but this requires a deeper understanding of LPMO catalysis.

Since the discovery of the LPMO reaction in 2010,^1^ headway has been made in elucidating the catalytic mechanism of these enzymes, and based on computational studies, it is now generally believed that the reactive copper species involved in hydrogen atom abstraction is a Cu(II)-oxyl species.^14−17^ Hydrogen atom abstraction by the Cu(II)-oxyl is followed by a rebound of the Cu-bound hydroxyl, leading to substrate hydroxylation and destabilization of the glycosidic bond.^4,16,18^ Reduction of LPMOs is essential for the enzymes to become catalytically competent and can be achieved by a variety of small reductants or electron-delivering enzymes.^19,20^ While LPMOs were originally thought to proceed via a monooxygenase mechanism (R–H + O_2_ + 2e^–^ + 2H^+^ → R–OH + H_2_O), evidence strongly suggests that under most, if not all, conditions, LPMOs catalyze a peroxygenase reaction (R–H + H_2_O_2_ → R – OH + H_2_O).^11,21,22^ The peroxygenase reaction is orders of magnitude faster than the apparent monooxygenase reaction.^11−13,21^ It is believed that LPMOs source H_2_O_2_ through an intrinsic oxidase activity,^23^ through autoxidation of low molecular weight reductants typically used in LPMO reactions,^24^ through exogenous oxidases,^25^ or through abiotic H_2_O_2_ generating sources such as irradiated lignin^26^ found in the same ecological niche. Predominant suggestions for the mechanism of the peroxygenase reaction entail initial homolytic cleavage of H_2_O_2_ by the reduced LPMO yielding a Cu(II)-hydroxide and a hydroxyl radical, where it is believed the latter can damage the LPMO if the LPMO is not bound to substrate to engage in productive chemistry.^22,27−29^

**Figure 1:**
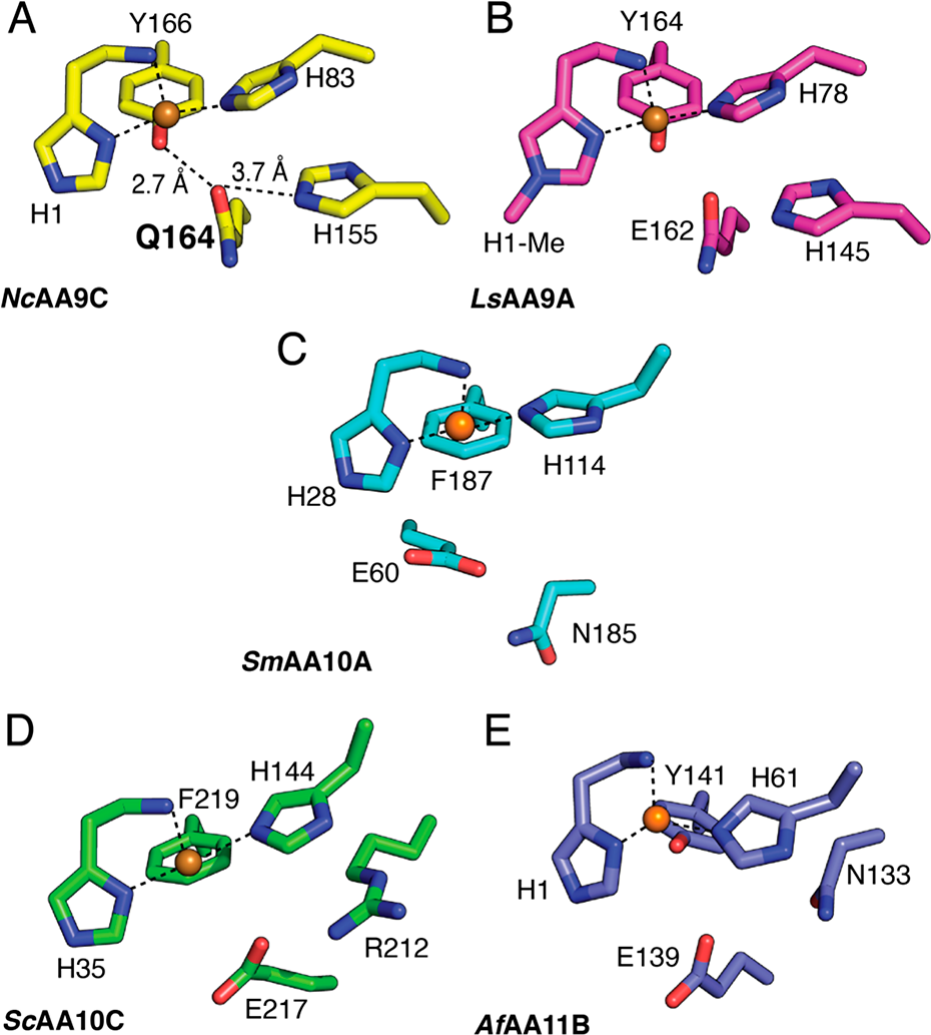
Structural comparison of the catalytic centers in various LPMOs. The histidine brace, key second sphere residues, and the copper center are shown. Examples from three LPMO families, AA9, AA10, and AA11 (classified as auxiliary activities, AA), are shown. (A) *Nc*AA9C (PDB entry 4D7U(36)), (B) *Ls*AA9A (PDB entry 5NLS(37)), (C) *Sm*AA10A (PDB entry 2BEM(34)), (D) *Sc*AA10C (PDB entry 4OY7(35)), and (E) *Af*AA11B for which a homology model is shown^38^ based on the crystal structure of *Ao*AA11 (PDB entry 4MAH(39)). The distance between key second sphere residues in *Nc*AA9C (A) was determined by using the measurement wizard in PyMOL.

Central to LPMO catalysis is a universally conserved Cu-histidine brace whereby the copper is coordinated by three nitrogen ligands, coming from the side chains of the N-terminal histidine and another, internal histidine, and the N-terminal amino group.^3,30^ This copper-binding motif is also found in other proteins, but the redox properties of these proteins differ, due to variations in the primary and second coordination sphere.^31−33^ While LPMOs have a strictly conserved histidine brace, the secondary coordination spheres of their copper centers show variation^34−39^ (Figure 1), and so does the reactivity of these centers.^12,38^ Notable variations in second sphere residues include a buried tyrosine or phenylalanine and a glutamine or glutamate (Figure 1). Notable variations in second sphere residues include a buried tyrosine or phenylalanine and a glutamine or glutamate (Figure 1). The importance of some second sphere residues has been investigated either experimentally^17,34,40−42^ or computationally,^16,43,44^ but studies combining both are scarce. These studies also do not address the role that second-sphere residues play in modulating copper reactivity or the importance of H_2_O_2_, with most studies performed prior to the discovery that H_2_O_2_ is the preferred cosubstrate.

The conserved second sphere glutamine/glutamate has received attention because mutational studies^41,42,45^ have shown that it is crucial for LPMO activity. However, these mutational studies were done before the discovery of the ability of LPMOs to act as peroxygenases and the susceptibility of LPMOs to autocatalytic inactivation. More recent mutational and computational studies have shown that Glu60 in *Sm*AA10A (Figure 1C) plays an important role in constraining and positioning H_2_O_2_ and the hydroxyl radical resulting from its homolytic cleavage.^17^ It was shown that mutation of this residue (Glu → Ala, Gln, Asn, Asp, or Ser) leads to decreased product formation and increased enzyme inactivation, where the latter suggests that this residue plays a role in confining the potentially damaging hydroxyl radical.^17,46^ Computational studies have suggested an analogous role for Gln162 in *Ls*AA9A (Figure 1B) in positioning H_2_O_2_ and in stabilizing the hydroxyl radical through hydrogen bonding.^16,43^ Importantly, while these previous studies highlight the importance of the conserved glutamine/glutamate in LPMO catalysis, how natural or engineered variation in this crucial second sphere residue affects the reactivity of the copper site remains an open question.

To decipher if and how the conserved glutamine/glutamate tailors copper reactivity, we have studied its role in a well-studied fungal family AA9 LPMO called *Nc*AA9C^23,36,47,48^ (Figure 1A). We have mutated Gln164 in *Nc*AA9C to three different amino acids (Glu, Asp, and Asn) and applied a vast range of biochemical, computational, and spectroscopic techniques to assess the role this residue plays in the myriad of reactions catalyzed by LPMOs and in controlling the reactivity of the copper active site. All mutants exhibited changes in LPMO functionality and stability, with the Q164E mutant showing drastic changes in copper site reactivity, resulting in a putative catalytic mechanism that differs from WT *Nc*AA9C.

## Results

### A Second Sphere Glutamine/Glutamate is Highly Conserved in AA9 LPMOs

**Figure 2:**
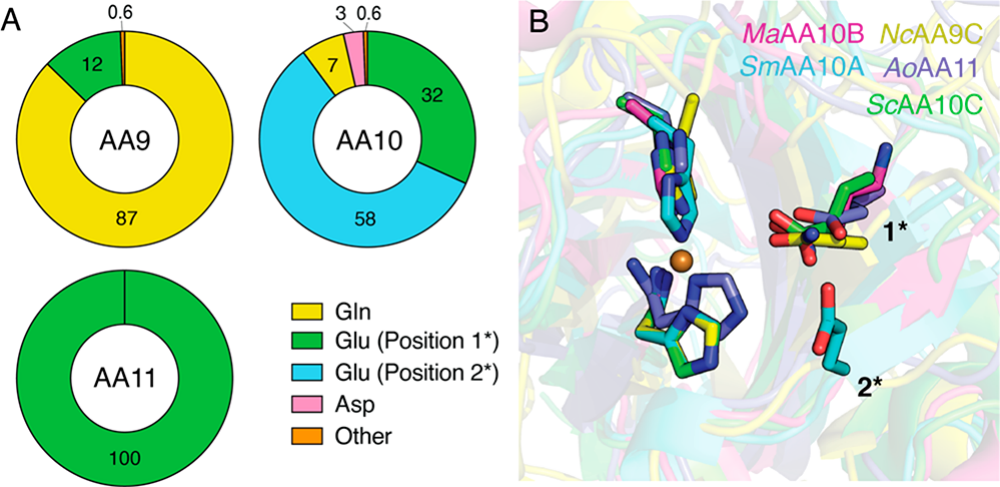
Structural alignment and residue frequency of the conserved glutamine/glutamate residue. (A) Percent frequency (%) of the different amino acids present at this position in the AA9, AA10, and AA11 LPMO families. Sequences were aligned with MAFFT,^51^ and homologous positions were selected after structural alignment in PyMOL. Note that the large majority of these LPMOs have not been functionally characterized and that all reported functionally characterized LPMOs have either a glutamine or 0glutamate. (B) Structural alignment of *Nc*AA9C (yellow; PDB 4D7U(36)), *Sc*AA10C (green; PDB 4OY7(35)), *Sm*AA10A (blue; PDB 2BEM(34)), *Ma*AA10B (pink; 5OPF^52^), and *Ao*AA11 (purple; 4MAH^39^). The copper bound by *Nc*AA9C is shown as an orange sphere; 1* and 2* indicate the different backbone locations of the glutamine/glutamate, as explained in the text.

Sequences were retrieved from the dbCAN2 server^49^ and used to create sequence alignments for the AA9 (472 sequences), AA10 (3820 sequences), and AA11 (166 sequences) LPMO families (Figure 2A), which represent the most abundant families in the CAZy^50^ database. A glutamine or glutamate residue is structurally conserved in almost all (>96%) LPMOs across these three families. This residue is located in the same place in the backbone for all AA9s, all AA11s and some AA10s (i.e., ScAA10C, position 1*; Figure 2B) however, is in a different position in the backbone in other AA10s (i.e., SmAA10A, position 2*; Figure 2B). Despite this difference, in both cases, the side chain headgroups appear in approximately the same position relative to the copper and the other active site residues.

The conserved glutamine in *Nc*AA9C (position 164) was mutated to a glutamate (Q164E), asparagine (Q164N), and aspartate (Q164D), enabling a study of both the impact of headgroup type and headgroup location on copper reactivity and LPMO functionality. All enzyme variants were derived from full-length *Nc*AA9C, containing a catalytic domain with an appended carbohydrate binding module (CBM1).

### Mutation of Gln164 Affects LPMO Performance

**Figure 3:**
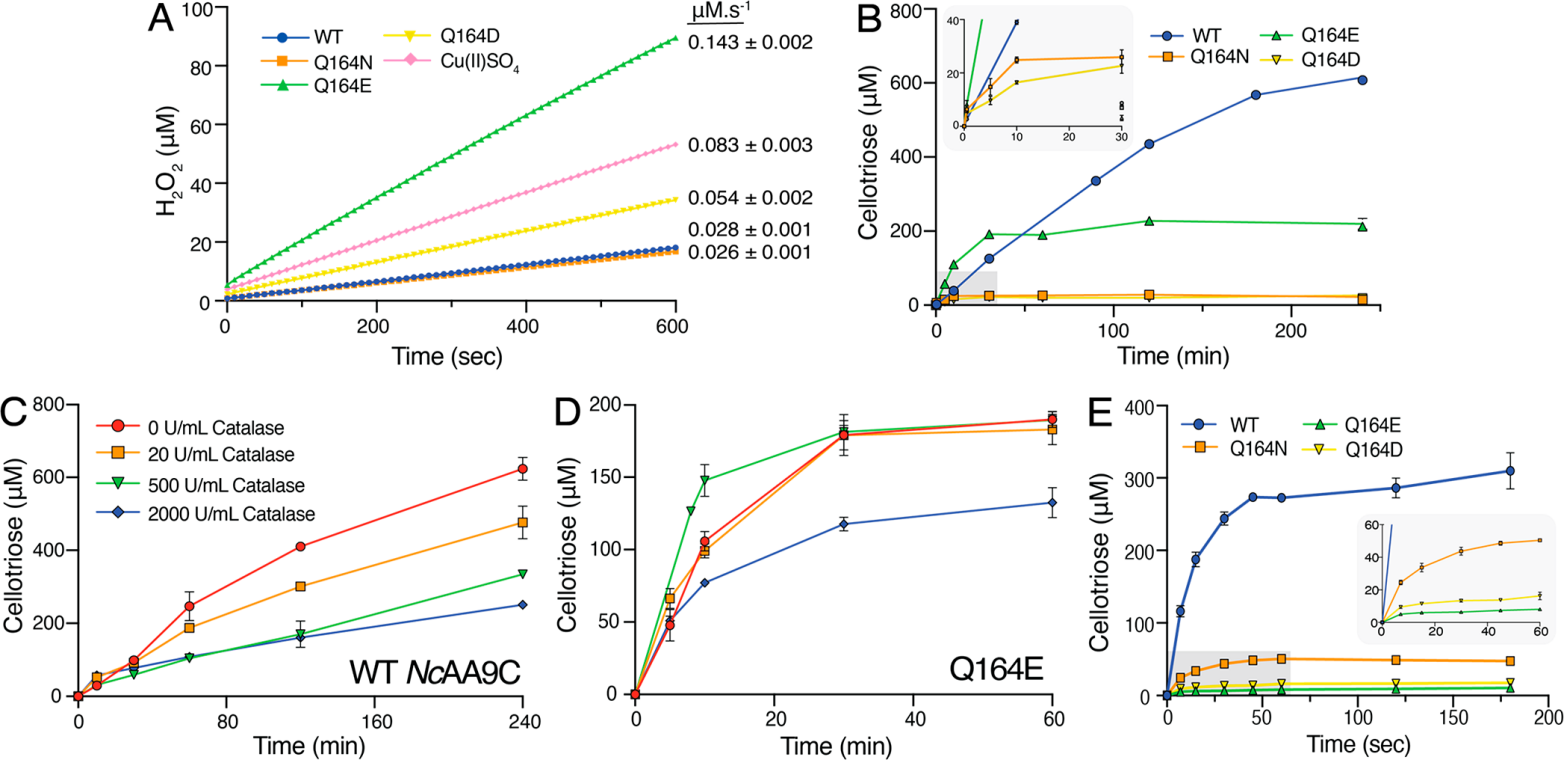
Role of H_2_O_2_ in the catalysis of WT *Nc*AA9C and the Gln164 mutants. (A) H_2_O_2_ production (oxidase activity) in the absence of polysaccharide substrate by 1 μM *Nc*AA9C variants in the presence of 1 mM AscA measured using the Amplex Red/HRP assay at 30 °C. (B-E) LPMO-catalyzed conversion of cellopentaose under different conditions. (B) Reductant-driven conversion of 1 mM cellopentaose by 2 μM *Nc*AA9C variants in the presence of 1 mM AscA. The initial rates for WT *Nc*AA9C and Q164E were determined from the first three time points and estimated to be 0.035 ± 0.001 and 0.093 ± 0.002 s^–1^, respectively. An accurate rate could not be determined for Q164N and Q164D due to rapid inactivation (see inset). (C, D) The same reactions as in panel B conducted in the presence of catalase (0–2000 U/mL) for WT *Nc*AA9C (C) and Q164E (D). (E) Conversion of cellopentaose by *Nc*AA9C variants in reactions fueled by externally added H_2_O_2_ (300 μM). Reactions contained 100 nM enzyme and 100 μM AscA (note the lower enzyme and AscA concentrations and different time scales of the *X*-axis relative to B–D). All reactions (A–E) contained 50 mM Bis-Tris at pH 6.5 and were performed at 37 °C unless otherwise stated. For all experiments, error bars show ±SD (*n* = 3; independent experiments).

Measurements of the oxidase activity of LPMOs, i.e., the ability to catalyze the reduction of O_2_ to H_2_O_2_, provide an easy tool to assess mutational effects on copper reactivity. Strikingly, the introduction of a carboxylic headgroup at position 164 led to clearly increased oxidase activity, with the Q164E and Q164D mutants exhibiting 5-fold and 2-fold rate enhancements, respectively (Figure 3A). On the other hand, the Q164N mutation did not affect the oxidase activity, underpinning the importance of the introduced aspartate and glutamate side chains. Importantly, changes in the oxidase activity are expected to also affect LPMO-catalyzed conversion of polysaccharide substrates in reductant-fueled reactions, since such reactions depend on *in situ* H_2_O_2_ production.^24,53,54^ It has been shown that the LPMO oxidase activity, rather than (LPMO-independent) autoxidation of the reductant, is the major source of H_2_O_2_ in ascorbate-driven reactions with AA9 LPMOs at neutral pH.^12^

The activity of *Nc*AA9C variants was assessed using the soluble cello-oligosaccharide cellopentaose, which is a very good substrate for this LPMO, with *k*_cat_ values surpassing 100 s^–1^ in reactions that are not limited by access to H_2_O_2_.^12^Figure 3B shows that the Q164N and Q164D mutants yielded minimal product formation, likely due to rapid enzyme inactivation. It thus appears that decreasing the side chain length and therefore proximity of the functional group to the copper makes the enzyme highly vulnerable to oxidative damage. This is not surprising considering the role of the glutamine/glutamate in confining both H_2_O_2_ and the hydroxyl radical which results upon its homolytic cleavage.^17,29^ While being less stable than the wild-type enzyme, the Q164E mutant is considerably more stable than the other two mutants (Figure 3B), allowing the detection of an increased catalytic rate, 2.6 times faster than WT *Nc*AA9C due to the increased in situ H_2_O_2_ production. The addition of various fresh reaction components after cessation of product formation by Q164E showed that only addition of fresh enzyme led to additional product formation (Figure S1), confirming that enzyme inactivation indeed took place.

To confirm the impact of H_2_O_2_ production on the performance of WT *Nc*AA9C and the Q164E mutant, reactions with the substrate were run in the presence of various concentrations of the H_2_O_2_ scavenging enzyme, catalase. It is important to note that even high concentrations of catalase (2000 U/mL) cannot completely inhibit a reaction catalyzed by *Nc*AA9C, as previously shown by Filandr et al.,^28^ which is due to the LPMOs having a *K*_M_ for H_2_O_2_ in the low micromolar range in the presence of substrate,^12,21^ while *K*_M_ values for catalases are in the millimolar range.^55^Figure 3 shows that reductant-driven conversion of cellopentaose by Q164E (Figure 3D) was less sensitive to catalase inhibition compared to WT *Nc*AA9C (Figure 3C). This is in accordance with the higher production of H_2_O_2_ by the mutant. Interestingly, the progress curves for Q164E in reactions with a midrange concentration of catalase (500 U/mL) seemed to show slightly increased initial rates which could be due to the catalase “balancing” the H_2_O_2_ concentration at a level that allows fast enzyme action while reducing enzyme inactivation.

Previous studies have shown that, when provided with sufficient amounts of a rapidly diffusing substrate such as cellopentaose, *Nc*AA9C can tolerate, and productively convert H_2_O_2_ supplied at concentrations as high as 1000 μM.^12^Figure 3E shows that, indeed, WT *Nc*AA9C rapidly turned over 300 μM H_2_O_2_ in a productive reaction. In contrast, the mutants, especially the aspartate- and glutamate-containing variants, were not capable of rapid productive consumption of H_2_O_2_ and showed rapid enzyme inactivation (Figure 3E, inset). By varying conditions (Figure S2), initial rate estimates for the Q164E mutant were obtained amounting to less than 1 s^–1^, which is at least 2 orders of magnitude slower than the wild-type enzyme.^12^

All in all, the functional data show mutational effects that can be explained by variations in the oxidase activity and the ability to handle H_2_O_2_ in a productive manner. The results show that the mutants cannot use H_2_O_2_ as efficiently as the wild-type enzyme and are more prone to oxidative inactivation under the turnover conditions.

### Effect of the Gln164 Mutation on the Redox Properties of the Copper Site

**Figure 4:**
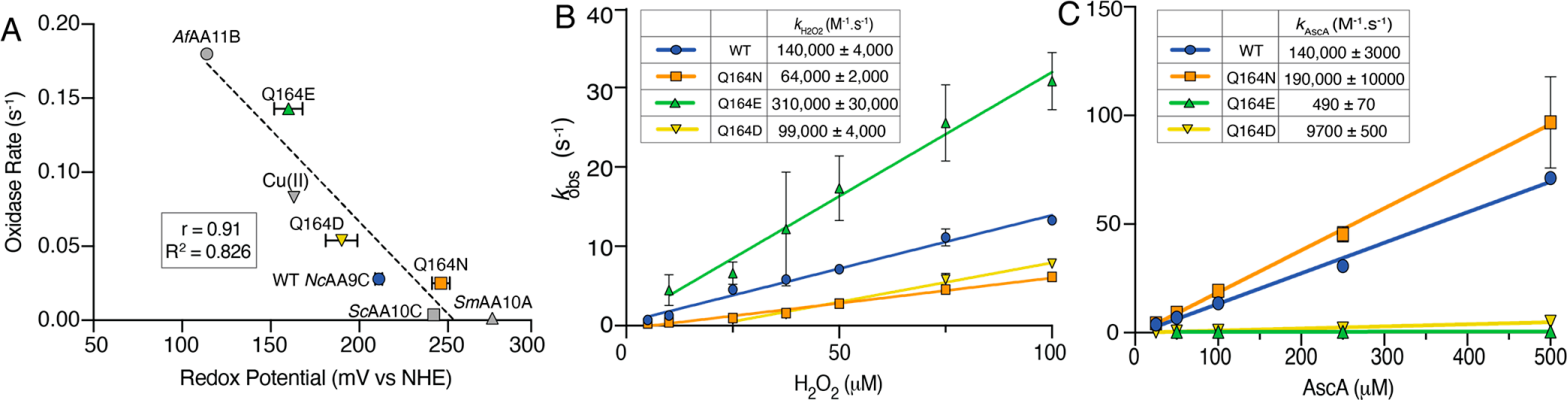
Redox potential and kinetics of reoxidation and reduction of WT *Nc*AA9C and Q164 mutants. (A) The reduction potentials (*E°*) for the *Nc*AA9C Cu(II)/Cu(I) redox couples (mV vs NHE) are plotted versus measured oxidase activities (s^–1^), with a linear best fit shown by a dotted line. Previously published reduction potentials and/or oxidase activities, determined using the same methods and conditions, for *Af*AA11B, *Sc*AA10C, and *Sm*AA10A are included.^35,38,57,58^ The oxidase activity for *Sm*AA10A was determined using the same method as that for the *Nc*AA9C variants. (B) Reoxidation of *Nc*AA9C variants by H_2_O_2_ under single turnover conditions and in the absence of substrate. (C) Reduction of *Nc*AA9C variants by AscA, measured by fluorescence stopped-flow spectroscopy. The rate constants (*k*_obs_) were plotted against the various H_2_O_2_ (B) or AscA concentrations (pseudo-first-order; C) to obtain the apparent second-order rate constant (*k*_H2O2_; B or *k*_AscA_; C) with the values indicated in the figure and listed in Table 1. For all experiments, error bars show ±SD (*n* = 3; independent experiments. Note in some cases the SD is less than the height of the marker used and is therefore not visible).

To gain insight into the electronic properties of the copper site in the *Nc*AA9C variants, the reduction potentials (mV vs NHE) were measured using the redox mediator TMP, as previously described.^36,56,57^ Relative to WT *Nc*AA9C (211 ± 2 mV), the Q164E and Q164D mutants showed lower reduction potentials (160 ± 8 and 190 ± 9 mV, respectively). On the other hand, the Q164N mutant showed an increased reduction potential of 246 ± 6 mV (Figure 4A and Table 1). Of note, the reduction potential for WT *Nc*AA9C (211 ± 2 mV at pH 6.5) compares well with a previously determined reduction potential of 224 ± 3 mV at pH 5.5.^36^ Both mutants with the lowest reduction potentials have anionic carboxylic acid side chains. It is conceivable that electrostatic effects imparted by the anionic residues modulate the copper site, resulting in diminished electron affinity and a less-positive reduction potential. Such an effect would be more pronounced the closer the carboxylic acid group is to the copper, as is indeed observed when Q164E is compared with Q164D. A less-positive reduction potential would also make the reduced LPMO more prone to oxidation by O_2_, which could explain the observed increase in oxidase activity for the Q164E and Q164D mutants (Figure 3A). Interestingly, a plot of measured reduction potentials versus measured oxidase activities including the four *Nc*AA9C variants, three previously studied LPMOs from other families, and free copper (Figure 4A) yielded a linear correlation coefficient of 0.91.

**Table 1:** Redox Properties of the *Nc*AA9C Variants^a^

	**Reduction potential (TMP assay, mV vs NHE)**	**Reduction(*****k***_**AscA**_**)(M**^**–1**^**·s**^**–1**^**)**	**Reoxidation(*****k***_**H2O2**_**)(M**^**–1**^**·s**^**–1**^**)**	**Ratio (Reduction/Reoxidation)**
***Sm*****AA10A**	275 ± 6	420,000	6,900	60
**Q164N**	246 ± 6	190,000	64,000	3
**WT*****Nc*****AA9C**	211 ± 2	140,000	140,000	1
**Q164D**	190 ± 9	9,700	99,000	0.1
**Q164E**	160 ± 8	490	310,000	0.002

a The second order rate constants for the rate of reduction (*k*_AscA_) and rate of reoxidation (*k*_H2O2_) for the *Nc*AA9C variants are derived from Figure 4, while the rates for *Sm*AA10A (at pH 7.0), shown for comparison, are derived from Bissaro et al.^17^ The rate of reduction was divided by the rate of reoxidation to determine the ratio between these two processes. The table also lists the experimentally determined redox potentials of the various enzymes (Figure 4A; the value for *Sm*AA10A is derived from Aachmann et al.^57^).

To gain further insight into the properties of the copper site we measured reduction and reoxidation rates using transient state kinetics, exploiting the fact that the oxidation state of the copper affects the fluorescence signal of the protein (Figure S3).^59^ The results are shown in Figure 4B and C and summarized in Table 1. The Q164E mutant exhibited a 2.2-fold higher reoxidation rate than WT *Nc*AA9C, while the Q164D mutant exhibited a slightly reduced reoxidation rate and the Q164N mutant exhibited an almost 2-fold lower rate (Figure 4B). Of note, this represents the first time that reoxidation by H_2_O_2_ is measured for an AA9 LPMO using fluorescence stopped-flow spectroscopy. Interestingly, these reoxidation rates are some 2 orders of magnitude higher compared to the reoxidation rate of an AA10 LPMO, *Sm*AA10A, measured using the same conditions^17^ (see below for further discussion).

Measurements of the reduction rates showed that this rate was drastically diminished in mutants containing an introduced carboxylic acid side chain (Q164E/Q164D) compared to WT *Nc*AA9C and the Q164N mutant, with the Q164E mutant exhibiting a drastic 285-fold lower rate than WT *Nc*AA9C (Figure 4C). In contrast, the impact of the Q164N mutation on the reduction rate was minimal, again underpinning the large impact of the nature of the headgroup at this position. The variation in the ratios of the reoxidation and reduction rates for the various enzymes correlates with the observed variation in the reduction potentials determined by using the TMP assay (Table 1). While the transient state rates cannot be directly (quantitatively) compared to the reduction potentials, since the observed processes are rather different, the similar trends observed for the rate ratios and the reduction potentials add confidence to the conclusion that the nature and position of the headgroup of residue 164 modulate copper electronics.

### Radical Formation (Hole Hopping) is Affected in the Q164E Mutant

All Gln164 mutants exhibited increased enzyme inactivation (relative to WT *Nc*AA9C), in both reductant-driven reactions (Figure 3B) and reactions with externally added H_2_O_2_ (Figure 3E). One method of protecting the enzyme against damage caused by reactive intermediates involves hole hopping, a process in which a series of “coupled” redox-active amino acids in the protein, primarily tyrosine and tryptophan residues, help to transfer reactive oxidizing radicals from the sensitive active site to the protein surface.^60^ For several AA9 LPMOs, including *Nc*AA9C, the formation and decay of radical species during the interaction of reduced enzymes with an oxidant has been demonstrated using UV–vis stopped-flow spectroscopy.^29,48,61,62^ These radical signals detected using UV–vis spectroscopy have been assigned as tryptophanyl and tyrosyl radicals using several methods including EPR and mutagenesis.^29,48,61,62^

**Figure 5:**
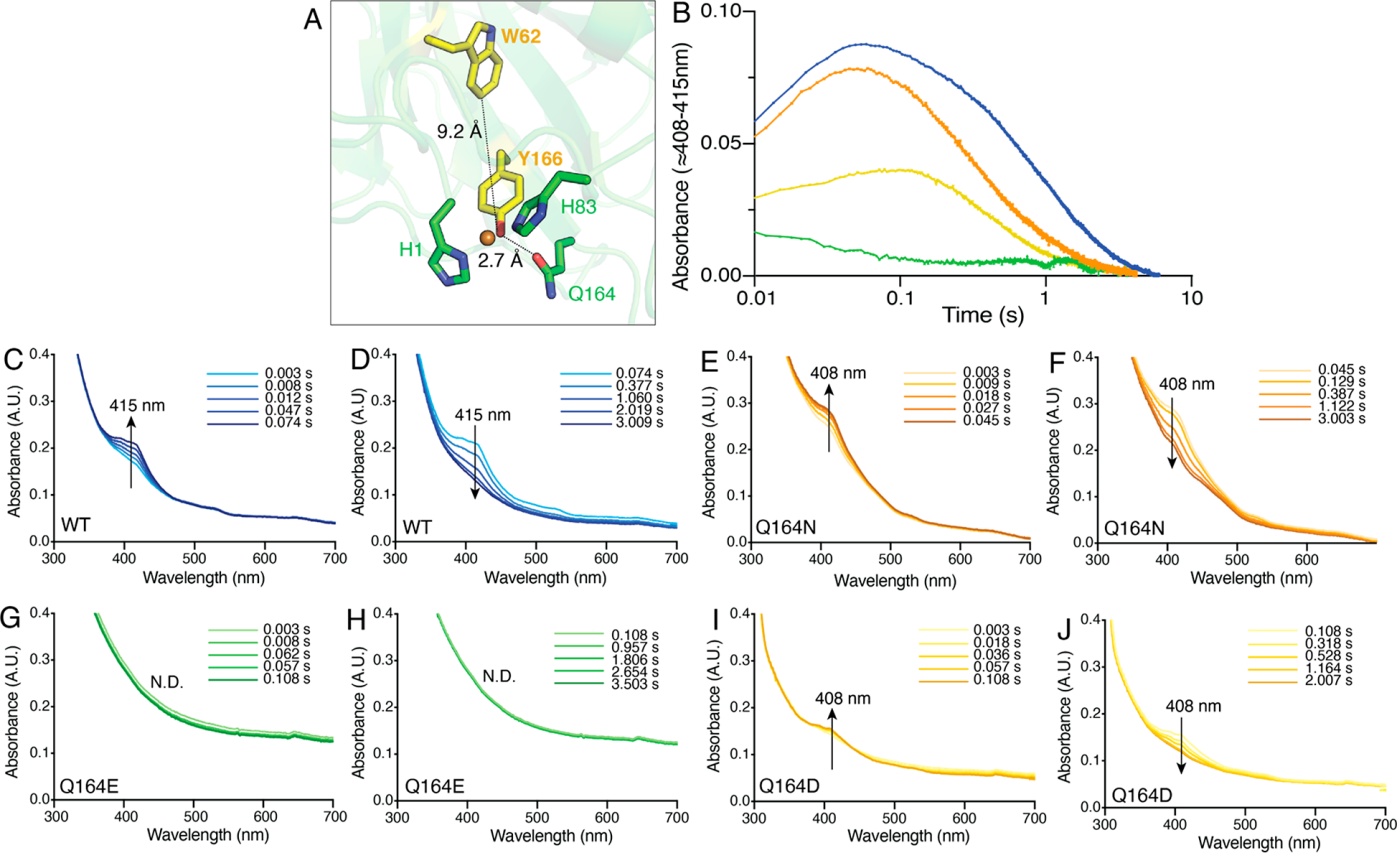
Radical formation and decay in *Nc*AA9C variants reacting with H_2_O_2_. (A) Structure of *Nc*AA9C (PDB entry 4D7U(36)) with the histidine brace (H1 and H83) and the mutated glutamine residue (Q164) shown as sticks with green carbons and the tyrosine (Y166) and tryptophan (W62) residues predicted to be involved in radical formation in *Nc*AA9C^48^ shown as sticks with yellow carbons. (B–J) Radical formation and decay. *Nc*AA9C variants in the Cu(II) state were mixed anaerobically with 1 mol equivalent of AscA in 50 mM Bis-Tris pH 6.5 at 4 °C to generate *Nc*AA9C–Cu(I). Stopped-flow transients were acquired in the absence of a substrate at 4 °C by reacting reduced enzyme with a 40-molar excess of H_2_O_2_. (B) Formation and decay of the ∼408–415 nm spectral feature for WT *Nc*AA9C (blue; details in panels C and D), Q164N (orange; details in E and F), Q164E (green; details in G and H), and Q164D (yellow; details I and J). N.D. = not detected. In the pairs of panels showing the transients, formation is shown in the first, left-hand panel (up arrow) and decay is shown in the right-hand panel (down arrow).

Radical formation and decay in all *Nc*AA9C variants was monitored by measuring spectral features predicted to be coming from the tyrosine (λ ≈ 408–415 nm) and tryptophan (λ ≈ 520 nm) residues^29,48^ found in a close proximity to the copper site (Figure 5A). The tyrosine signal was of particular interest due to the proximity (2.7 Å) of the tyrosine residue (Y166) to the mutated glutamine residue (Q164) (Figure 5A). The observed formation and decay of a tyrosyl radical in WT *Nc*AA9C were comparable to the signals observed by Hedison et al.;^48^ however, compared to this previous study the tryptophanyl signal was weaker (Figure S4). While the Q164N mutant was similar to the wild-type in terms of formation and decay of the ∼408–415 nm feature, the two variants with introduced carboxylic acid head groups showed clear changes. A weaker feature, at a slightly different wavelength (408 rather than 415 nm), was observed for the Q164D mutant, with slower formation (within 100 ms, rather than 50 ms) and slower decay (Figure 5B, E, and F). In the case of the Q164E mutant, no spectral features were detectable (Figure 5B, G, H), meaning that radical formation and decay were either very fast and happen within the deadtime of the instrument (2.9 ms; for the parameters used in this setup) or did not happen at all for the duration of the experiment (4 s). These observations suggest that an amide containing side chain at position 164 is crucial for formation of the tyrosyl radical. Considering that this tyrosine may be part of a downstream hole hopping network that may protect the enzyme from inactivation, it is interesting to note the correlation between reduced formation of the tyrosyl radical in the Q164E and Q164D mutants and the increased sensitivity to inactivation under turnover conditions (Figure 3B and E). The results suggest that, for the Q164E and Q164D mutants, the increased sensitivity to oxidative damage is due in part to disruption of a protective hole hopping pathway.

### The Cu(II) Ligand Field is Unperturbed by Mutation at Position 164

**Figure 6:**
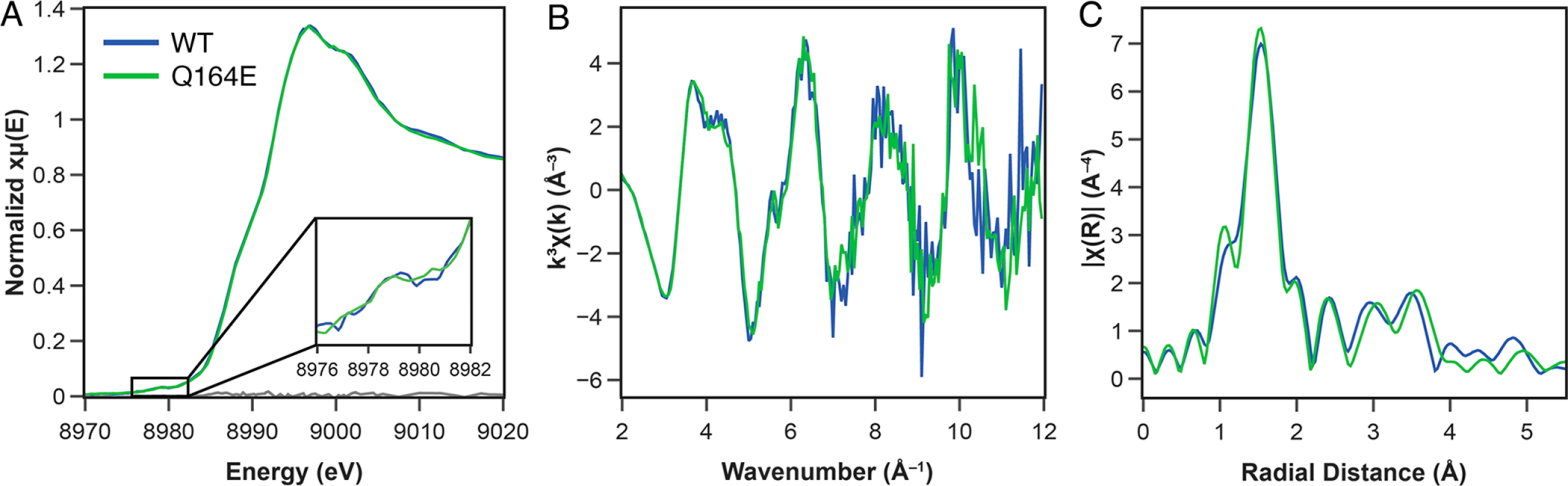
Cu K-edge X-ray absorption data for WT *Nc*AA9C and Q164E. (A) PFY-detected Cu K-edges, with the 1*s* → 3*d* feature at 8976–8982 eV enlarged in the inset. (B) *k*^3^-weighted EXAFS and (C) Nonphase shifted Fourier transform over a *k*-range of 2 to 12 Å^–1^.

X-band EPR spectroscopy at 30 K showed expected and essentially identical EPR behavior for all *Nc*AA9C variants (Figure S5, Tables S1, S2). This shows that mutation of Gln164 does not result in notable direct changes to the Cu(II) ligand field in the resting state. Further support for the largely unperturbed Cu(II) sites in the *Nc*AA9C variants was obtained from Cu K-edge X-ray absorption spectroscopy (XAS) data obtained for WT *Nc*AA9C and the Q164E mutant. The Cu K-edges of both variants (Figure 6) were nearly identical. Both spectra exhibit a weak, dipole forbidden 1*s* → 3*d* transition at 8979 eV. In agreement with what is seen for the edges, the observed *k*^3^-weighted extended X-ray absorption fine structure (EXAFS) and resultant Fourier-transformed (FT) data also approximately overlap (Figure 6B and C, respectively). Fits to the FT-EXAFS data show that the data are best fit as a 5- or 6-coordinate Cu site with a primary shell (*N* = 4 or 5) at 2.0 Å and a second shell (*N* = 1) at 2.2–2.3 Å (Figure S6, Tables S3 and S4). Fitting of the long-distance data necessitated the inclusion of multiscattering paths from two His ligands, and it was found that fits MS1 and MS2 (Tables S3 and S4, Figures S7 and S8) provided equally good fits. Further remarks on EXAFS fitting are provided in the Supporting Information.

### Density Functional Theory (DFT) Cluster Model Calculations

To further investigate the impact of the Q164E mutation on the reoxidation rate with H_2_O_2_, DFT cluster model calculations in the absence of a substrate were performed. The model consisted of H_2_O_2_ bound to the *Nc*AA9C–Cu(I) active site composed of His1, His83, and Tyr166 and the second-sphere residues His155 and Gln164 or Glu164 for the wild-type or the Q164E mutant, respectively (Figure 1A). The H_2_O_2_ molecule was placed such as to interact with His155 and Gln/Glu164 based on the results of previous computational studies.^16,17,43,63^ These previous studies have shown that LPMO reactivity is associated with an intricate fluctuating H-bonding network, which in the current model depends on the protonation state of His155 (HID or HIE tautomeric forms or HIP) and Glu164 (for the mutant). Recent neutron diffraction and high-resolution X-ray diffraction studies on other AA9s have shown His155 likely does not occur in the double protonated form, not even at acidic pH.^64,65^ The initial reactivity calculations along the HO–OH bond cleavage pathway featured His155 with the proton at the N^ε^ position (HIE). The choice of HIE155 is supported by neutron diffraction studies^64,66^ of other AA9s from *Neurospora crassa* and the higher stability of HIE compared to HID in QM/MM calculations.^43^ The lack of similar data on the Q164E mutant motivated us to explore both protonation states of Glu164.

**Figure 7:**
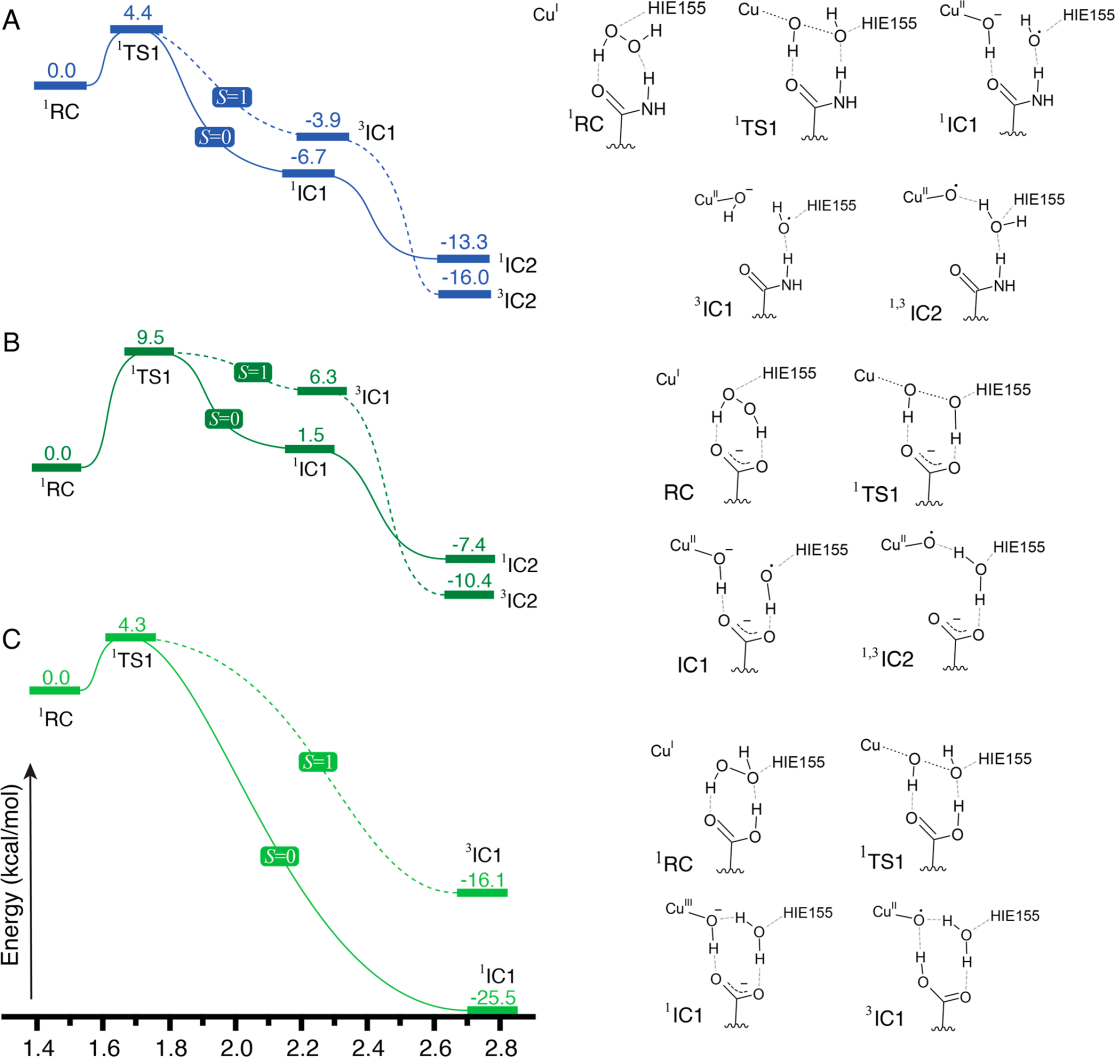
Relative electronic energies in kilocalories per mole of minima and transition states along the H_2_O_2_ splitting path of the reaction with *Nc*AA9C variants in the Cu(I) state. The panels depict the reactions for WT *Nc*AA9C (A), and the Q164E mutant with Glu164 in the glutamate (B) or glutamic acid (C) form. Solid and dashed lines represent singlet and triplet manifolds, respectively. Hydrogen bonds are shown as gray dashes. HIE155 represents His155 donating a hydrogen bond via the N^ε^ proton. RC: reactant complex; TS: transition state; IC: intermediate complex. The left superscript represents the spin multiplicity. Figures S10 and S15 show the optimized geometries and spin density plots of the structures depicted here.

The optimized protein-bound H_2_O_2_ model for the Cu(I) state of WT *Nc*AA9C (^1^RC in Figure 7A and Figure S10) revealed the N^ε^ proton of HIE155 H-bonded to either the proximal or distal oxygen atom of H_2_O_2_ (isoenergetic structures). The H_2_O_2_ binding mode is similar to that reported elsewhere.^63^ The first transition ^1^TS1 state on the broken-spin singlet surface is found at an O–O bond length of 1.68 Å, with electronic energy 4.4 kcal/mol above the initial minimum. The electronic structure of the [CuH_2_O_2_]^+^ fragment after the homolytic cleavage is similar to that previously reported for *Sm*AA10A.^17^ This intermediate consists of a Cu(II)-hydroxide H-bonded to the Gln164 carbonyl group and a hydroxyl radical stabilized by H-bonding to HIE155 and the Gln164 NH_2_ group, as inferred from the larger spin population on the distal oxygen (Table S5). Despite the distance between the unpaired electrons, the open-shell singlet ^1^IC1 is 2.8 kcal/mol lower than ^3^IC1 in the triplet state due to the presence of the Cu–OH···carbonyl(Gln164) hydrogen bond in the optimized geometry (see Figures S10 and S11).

The postulated Cu(II)-oxyl intermediate is generated by a barrierless hydrogen-atom transfer (HAT) in which the hydroxyl radical abstracts a hydrogen from the copper-bound hydroxide to form water. It is worth mentioning that other computational studies also have shown that this step has a small^16,17^ or vanishing^43^ barrier, as pointed out by Hagemann and Hedegård.^67^ The spin-coupled open-shell singlet minimum ^1^IC2 is at −13.3 kcal/mol, with Cu–O and O–O distances of 1.90 and 2.63 Å respectively. The geometry of ^3^IC2 at the triplet spin state is nearly identical to ^1^IC2, and 2.7 kcal/mol more stable, which is in agreement with previous reports on computational modeling of the Cu(II)-oxyl in LPMOs.^16,17,43,67^

Adopting compelling suggestions by Bissaro et al.^17^ that the fluorescence quenching upon reaction of Cu(I)-LPMO with H_2_O_2_ is associated with the first electron transfer, i.e. formation of the Cu(II)-hydroxide and the hydroxyl, we focus on the first transition state to rationalize the effect of the Q164E mutation on the reoxidation rate of *Nc*AA9C. Replacing Gln164 for Glu164 in the anionic form leads to a distinct H_2_O_2_ binding mode: both its hydrogens are H-bonded to the carboxylate group, causing the dihedral to decrease from 140° in the WT to 43.5°. Despite the more unfavorable conformation of bound H_2_O_2_, the first transition state was found at 9.5 kcal/mol (^1^RC in Figure 7B and Figures S12 and S13), which is more than two times higher compared to the reaction with WT *Nc*AA9C (Figure 7A). This much higher barrier lies in stark opposition to the experimental rate trend, showing that reoxidation by H_2_O_2_ is faster for the mutant. In the intermediates ^1,3^IC1 and ^1,3^IC2, the distances between the oxygen atoms are ca. 0.06 and 0.10 Å longer than in WT *Nc*AA9C, as a result of the electrostatic repulsion between the Glu164 carboxylate and hydroxide bound to Cu(II). The electric polarization effect on H_2_O_2_ induced by the negative charge placed approximately along the O–O bond may be the reason that homolytic cleavage is only possible with a high electronic energetic penalty.

At the experimental pH value (6.5), protonation of the Glu164 acid group to an appreciable degree is reasonable and, hence, worth exploring. Several studies have concluded that the nearby His155 remains uncharged in glutamine containing wild-type AA9s even at acidic pH^64,65^ and the nearby presence of this acidic His could lead to an elevated p*K*_a_ for Glu164. The optimized H_2_O_2_-bound Cu(I) structure with the glutamic acid (^1^RC in Figure 7C and Figures S14 and S15) shows a similar H-bonding pattern and H_2_O_2_ dihedral as in WT *Nc*AA9C. The transition state ^1^TS1 is found at nearly the same O–O bond distance (1.68 Å), 4.3 kcal/mol above the first minimum. The lower energy barrier relative to the calculation for WT *Nc*AA9C, although below the expected accuracy of DFT, agrees with the experimentally determined higher reoxidation rate measured for the Q164E mutant. Importantly, however, the barrier obtained with glutamic acid is much lower compared with the barrier obtained with glutamate, indicating that the fast oxidase reaction observed for the Q164E mutant involves a protonated, neutral side chain.

Interestingly, in the products ^1^IC1 or ^3^IC1, the distal OH group deprotonates the Glu164 carboxylic acid chain to form water, differently from the HAT in WT *Nc*AA9C. The resulting copper-bound OH group is H-bonded to the Glu164 carboxylate in the singlet state (^1^IC1), contributing to its stability (−25.5 kcal/mol) relative to ^3^IC1 (−16.1 kcal/mol). The Cu(II)-oxyl species, ^3^IC1, whose reactivity is deemed key for substrate activation, is formed only in the triplet spin multiplicity by a proton transfer to Glu164. However, on the singlet spin state, the resulting structure ^1^IC1 has a closed shell configuration. The Cu–O distance of ^1^IC1 (1.81 Å) is significantly lower than that of its ^3^IC1 counterpart (1.92 Å). The charge population on the Cu atom indicated a higher positive charge in ^1^IC1 (q_Cu_ = 0.36) relative to ^3^IC1 (q_Cu_ = 0.27), while the proximal O atom is more negatively charged in ^1^IC1 (q_O_ = −0.27) than in ^3^IC1 (q_O_ = −0.17). The Cu–O distance, spin, and charge populations are indicative of a Cu(III)-hydroxide species. The calculated Cu–O distance shows congruency with Cu(III)–OH distances in model complexes: Tolman and co-workers have reported a series of diamagnetic HAT-competent Cu(III)-hydroxide model complexes with a Cu–O distance of 1.80–1.81 Å determined by resonance Raman and corroborated by DFT calculations.^68,69^ This shows that the Cu(III)–OH found herein for the H_2_O_2_ activation with the protonated Q164E model finds precedence in synthetic chemistry.

A possible alternative mechanism in the Q164E mutant may also help explain why no tyrosyl/tryptophanyl radical formation is observed for Q164E (Figure 5G and H). Instead of a Cu(II)-oxyl with a triplet spin ground state as in the WT, the formation of a Cu(III)–OH species with a singlet spin state may allow for faster spin-allowed formation of a Cu(II)-tyrosyl species, which has been shown to be a singlet spin state in *Ls*AA9A after reaction with H_2_O_2_ in the absence of substrate.^61^ The intermediate states arising during a reaction involving a Cu(III)–OH species may simply not trigger a protective mechanism that has evolved to deal with hydroxyl radicals or a Cu(II)-oxyl species.

**Figure 8:**
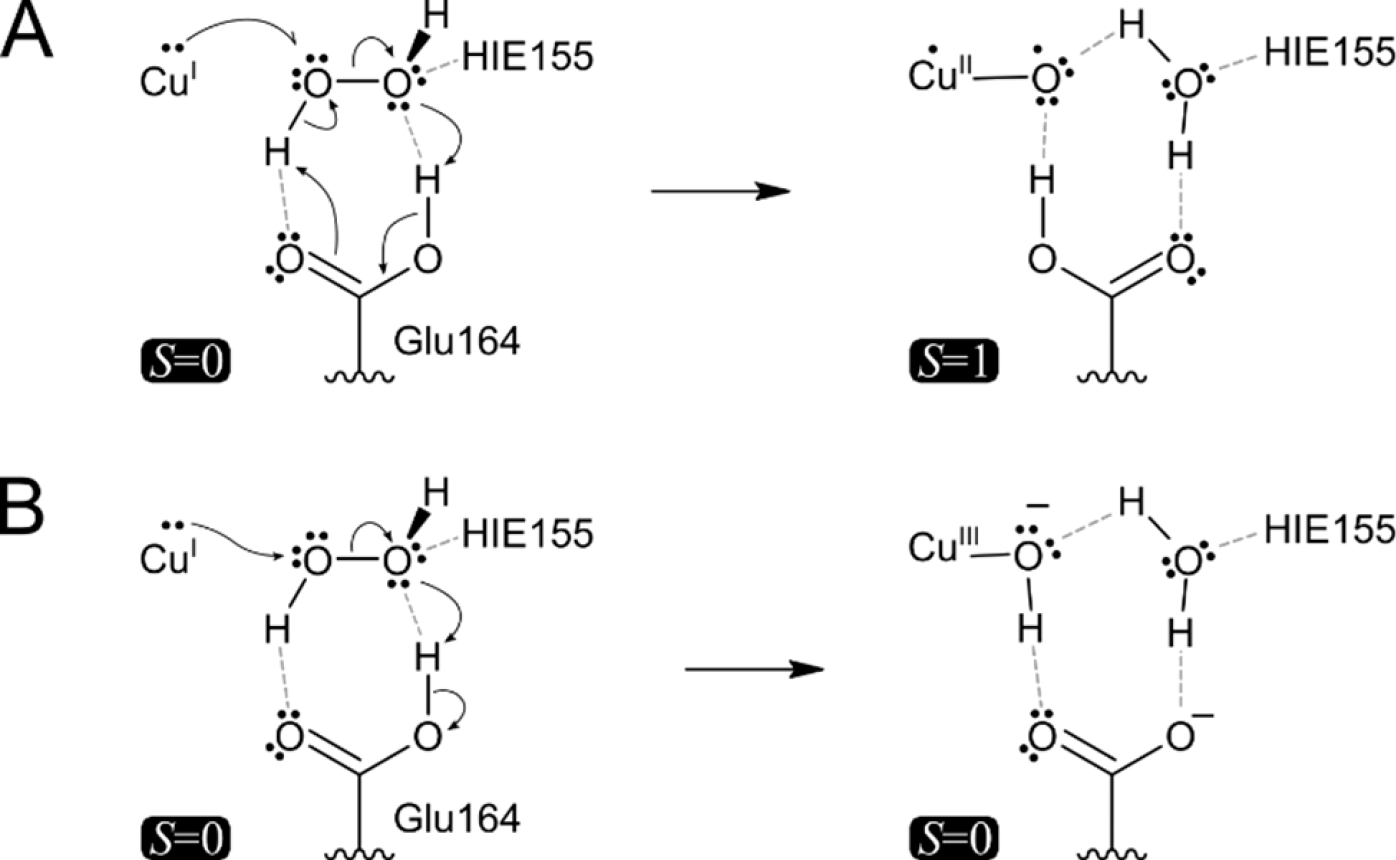
Proposed net electronic rearrangement in triplet and singlet state intermediate species in the protonated Q164E mutant. (A) The closed-shell H_2_O_2_-bound Q164E mutant in the Cu(I) oxidation state converts to the Cu(II)-oxyl species at the triplet state without the formation of Cu(II)-hydroxide and a hydroxyl radical and without a hydrogen-atom abstraction step. (B) In the singlet spin state manifold, the Cu(III)-hydroxide product is formed without the Cu(II)-hydroxide intermediate. The complete cluster models are depicted in Figure S14.

To summarize the insights from the protonated Q164E model computations, Figure 8A illustrates the net rearrangement generating the reactive Cu(II)-oxyl species, implying singlet–triplet surface crossing but without the formation of a Cu(II)-hydroxide/caged hydroxyl radical intermediate and without an HAT step. On the singlet manifold (Figure 8B), the polarization of the O–O bond due to the two H-bonds to the distal oxygen atom favors the nucleophilic attack of the Cu atom on the O–O σ* orbital, with H_2_O as an effective leaving group and a resulting net two-electron oxidation of the Cu center. Similar rationale has been applied to H_2_O_2_ activation by biomimetic heme hydroperoxidases featuring a pendant H-bond donor near the metal.^70,71^

## Discussion

The present results show that the conserved Gln/Glu second sphere residue in LPMOs (Figure 1) has a profound multifaceted effect on copper reactivity, beyond its known role in shaping the catalytic center and caging the oxygen species. Importantly, both EPR and X-ray absorption data showed that the primary Cu(II) coordination sphere was virtually identical between the mutants addressed in this study. Thus, the observed effects are truly “second sphere” and do not involve changes in atomic copper–enzyme contacts. Such purely second sphere effects have also been observed in studies of the type-1 Cu protein azurin, for which it was demonstrated that secondary sphere mutations could tune the reduction potential of the Cu site via modulation of the surrounding hydrophobicity and hydrogen-bonding network, without significant impact on the Cu binding site.^72^

The introduction of an anionic carboxylic acid group at this position (Glu or Asp) decreased the reduction potential of *Nc*AA9C–Cu(II) and increased the oxidase activity. The oxidase activity can be viewed as a proxy for reoxidation by O_2_ as the first step of this reaction involves the formation of O_2_^•–^.^73^ The fluorescence stopped-flow data showed changes in the ratio of the rate of reduction by ascorbate and the rate of reoxidation by H_2_O_2_ that align well with the observed changes in the reduction potential of the *Nc*AA9C–Cu(II)/*Nc*AA9C–Cu(I) couple. Generally, the decrease in the reduction rates was larger than the increase in the reoxidation rates and the effects were largest upon introduction of a carboxylic acid headgroup (Glu and Asp). Considering the negative charge of this headgroup, it is not surprising that its presence favors the Cu(II) state (relative to the wild-type). The reduction/reoxidation rate ratio diminished by 500-fold for the Q164E mutant, in which the negatively charged headgroup is placed close to the copper. The change was less dramatic (10-fold decrease) for the Q164D mutant, in which the headgroup is further away from the copper. Interestingly, the Q164N mutant showed a slightly increased reduction potential and in this case the reduction/reoxidation rate ratio increased by 3-fold. Clearly, mutation of the Gln164 second sphere residue has huge effects on copper reactivity.

Assuming homolytic cleavage of H_2_O_2_,^16,17,22,29^ residue 164 will play a crucial role in confining the emerging hydroxyl radical and any deterioration of its ability to do so will increase the chances for off-pathway reactions leading to oxidative damage to the enzyme. Indeed, all mutants showed a decreased ability to interact productively with H_2_O_2_. Using UV–vis spectroscopy for the detection of radical formation, we show here that mutational effects on the stability of LPMOs under turnover conditions may also relate to changes in protective hole hopping pathways. We detected no (Q164E) or strongly reduced (Q164D) radical formation for the two mutants that were most prone to damage in reactions containing supplemented H_2_O_2_. Importantly, the lack of radical formation in Q164E has several possible explanations: (1) Q164E-Cu(I) is not reacting with H_2_O_2_, (2) radical formation is occurring either too fast or too slow to be measured, or (3) the reactive “hopping” species formed in WT *Nc*AA9C is not formed in the reaction with Q164E and/or is directed to elsewhere in the protein resulting in no UV–visible feature. Hypothesis (1) can be eliminated, as reoxidation of Q164E-Cu(I) by H_2_O_2_ was observed by fluorescence stopped-flow (Figure 4B). Hypothesis (2) seems unlikely as the dead-time of the stopped-flow instrument is only 2.9 ms (minimum time), and the reaction was monitored over 4 s in total (maximum time), with 1.5 ms steps. The formation and decay rates previously measured for tyrosyl and tryptophanyl radicals fall well within this time scale,^29,61^ suggesting if a radical was being formed at these positions it should have been detected during this time frame. Still, as alluded to above, it is conceivable that hole hopping in the Q164E mutant is faster than that in the wild-type enzyme. Hypothesis (3) seems plausible since our DFT calculations lead to the remarkable conclusion that, in the Q164E mutant, the radicals involved in hole hopping are not being formed due to a change in the reoxidation mechanism.

Our DFT cluster model calculations indicated that the protonation state of glutamate is of crucial importance. If the solvent-exposed Glu164 were fully deprotonated, the calculations predict that reoxidation of Q164E would be much slower than WT *Nc*AA9C, in stark contrast to the experimental results. However, incorporation of a protonated Glu164 residue resulted in a slightly lower energetic barrier, which is consistent with the higher reoxidation rate observed experimentally. During the revision of this manuscript, Lim et al.,^63^ reported that the H_2_O_2_ binding step may have relevant role in the reoxidation kinetics. The calculation of binding constants and measurement of reoxidation rates at different pH values will be the subjects of future studies. Importantly, the present DFT calculations with protonated Glu164 suggest a mechanistic change, whereby a reactive Cu(III)-hydroxide species is formed in a single step on the H_2_O_2_ splitting pathway, in contrast to the Cu(II)-hydroxide and hydroxyl radical intermediates formed in WT *Nc*AA9C. While the reactive Cu(III)-hydroxide species has been suggested as the C–H activating intermediate in LPMO based on small models^74,75^ and calculations indirectly tied to experimental data,^14,43,76^ for the first time this species is proposed to have biological relevance from a calculation associated with experimental reaction rates. The stabilization of the high oxidation state in AA9 LPMOs is often considered correlated with the deprotonation of the terminal amine^14,74,77^ or the axial tyrosine^27^ to enhance the electron donation. Our calculation with the protonated E164 suggests that the deprotonation may not necessarily happen in the first coordination sphere but in the second, as a means to stabilize the Cu(III)–OH via a rearranged H-bonding network. Of note, a recent modeling study by Torbjörnsson et al.^27^ predicts that auto-oxidative damage to the enzyme does not necessarily involve a hydroxyl radical, aligning with the observation that the Q164E mutant, with its potentially changed catalytic mechanism, still suffers from inactivation.

While the present results point at Gln164 as an important modulator of LPMO reactivity, this reactivity is also affected by additional second sphere features, with evidence pointing toward the tyrosine/phenylalanine (Tyr166 in *Nc*AA9C) and to His155. AA9 LPMOs contain the His(155)-Gln(164)-Tyr(166) combination, whereas many AA10 LPMOs, such as chitin-active *Sm*AA10A and cellulose-active *Sc*AA10C, lack His155 and contain a glutamate-phenylalanine combination (Figure 1). Underpinning the importance of additional second sphere residues, *Sm*AA10A, despite having a glutamate instead of a glutamine, has a high reduction potential and reduction rate (Table 1) and its oxidase activity is much lower (Figure 4A) compared to the Q164E mutant of *Nc*AA9C. A very low oxidase rate has also been shown for *Sc*AA10C.^58^ Interestingly, an AA11 family LPMO called *Af*AA11B, which as a glutamate-tyrosine combination but lacks the histidine (replaced by an asparagine), showed an even higher oxidase activity^38^ than the Q164E mutant of *Nc*AA9C (Figure 4B). Although more work is needed to fully unravel second sphere effects, current data indicate that the glutamate-tyrosine combination, as in the Q164E mutant of *Nc*AA9C and *Af*AA11B, is associated with elevated oxidase activity, while a glutamate-phenylalanine combination is associated with low oxidase activity. It is worth noting that nature has apparently evolved multiple copper site arrangements in LPMOs, which has functional implications. This could be relevant from an evolutionary perspective and may, for organisms with multiple LPMOs, allow adaptation to changes in the redox state of the environment and the availability of H_2_O_2_.

In conclusion, this study shows how a conserved glutamine/glutamate in the second coordination sphere fine-tunes copper reactivity and affects the LPMO functionality. Due to effects on copper reactivity and on the geometry and hydrogen bonding networks of the catalytic center, this residue affects all aspects of LPMO reactivity including oxidase activity (H_2_O_2_ production), peroxygenase activity, enzyme stability under turnover conditions, reduction and reoxidation of the active site copper, and radical formation, which may be related to a protective hole hopping mechanism. It has been pointed out that the histidine brace found in LPMOs may be “nature’s copper alternative to haem”.^78^ The present study shows that, just as the functionality of haem enzymes depends on subtle modulations in the environment surrounding the cofactor,^79^ the catalytic performance of the histidine brace is determined by its fine-tuned environment. Next to providing insight into LPMO functionality, the present results expand the knowledge base for LPMO-inspired design of synthetic Cu-catalysts.^9,80^ Clearly, emulating LPMO-like catalysis in a synthetic catalyst requires more than a histidine-brace. Hopefully, emerging knowledge on LPMOs will eventually allow the design of enzymes and/or synthetic catalysts capable of activating a range of high-energy C–H bonds in substrates other than polysaccharides.

## Experimental Procedures

### Cloning

Plasmid design and molecular *in silico* design were performed with SnapGene (Chicago, IL, USA). *De novo* synthesis of the LPMO genes and cloning into the pBSYP_*GCW14*_Z *P. pastoris*/*E. coli* shuttle plasmid were done using the BioXP 3000 system (SGI-DNA, Inc., San Diego, CA, USA). The DNA sequence of WT *Nc*AA9C (AN: XP_965598) including the natural signal peptide was codon optimized for *P. pastoris* and had previously been cloned into the expression vector pBSY_*GCW14*_Z.^81^ To exchange the glutamine at position 164, two nucleotides had to be exchanged for Q164N (C538A and A540C) and Q164D (C538G and A540T) while only a point mutation was required for the Q164E (C538G) variant.

### *P. pastoris* Transformation and Initial Screening

The sequence verified LPMO expression plasmids were linearized with *Smi*I and used to transform killer plasmid-free *P. pastoris* BSYBG11 (Mut^S^, Δ*AOX1*) one-shot ready competent cells (Bisy GmbH, Hofstätten a. d. Raab, Austria), according to the manufacturer’s instructions. After 48 h of growth on YPD (1% (w/v) yeast extract, 2% (w/v) peptone and 2% (w/v) dextrose) with 100 μg/mL Zeocin, 24 transformants were selected and picked for small scale cultivation and subsequent expression screening, as described previously.^81^

### Expression and Purification

WT *Nc*AA9C and mutants were expressed and purified as discussed previously^81^ and are briefly described here. A 2 L baffled flask containing 500 mL of YPD was inoculated with a single yeast colony and grown for 60 h at 28 °C, 180 rpm. Cells and supernatant were collected by centrifuging at 10,000 × *g* for 20 min at 4 °C, and the resulting supernatant was filtered through a 0.22 μM Steritop filter (Merck Millipore, Burlington, MA, USA). The supernatant was concentrated 10-fold using a VivaFlow 200 tangential flow filtration system with a molecular weight cutoff (MWCO) of 10 kDa (Sartorius, Göttingen, Germany). Ammonium sulfate was added to the concentrated supernatant to a final concentration of 2.4 M prior to purification by hydrophobic interaction chromatography (HIC). The supernatant was loaded onto a 5 mL HiTrap Phenyl FF column (Cytiva, Marlborough, MA, USA) equilibrated with 50 mM Bis-Tris pH 6.5, 2.4 M ammonium sulfate. The protein was eluted by applying a linear gradient of 0–100% 50 mM Bis-Tris pH 6.5 over 40 mL, using a flow rate of 2 mL/min. Fractions were analyzed by SDS-PAGE (Biorad, Hercules, CA, USA), and those containing the correct protein were pooled and concentrated to 1 mL using an Amicon Ultra-15 10 kDa MWCO centrifugal filter unit (Merck Millipore, Burlington, MA, USA). The concentrated protein sample was loaded onto a HiLoad 16/60 Superdex 75 size exclusion column (Cytiva, Marlborough, MA, USA) equilibrated with 50 mM Bis-Tris pH 6.5, 200 mM NaCl. The protein eluted after 40 min at a flow rate of 1 mL/min. Protein purity was assessed by SDS PAGE, and fractions containing the correct protein were pooled. A 3-molar excess of Cu(II)SO_4_ was added to the protein sample followed by incubation on ice for 60 min. Excess copper and salt were removed by washing with 50 mM Bis-Tris pH 6.5 using an Amicon Ultra-15 10 kDa MWCO centrifugal filter unit until the copper had been diluted at least 1 × 10^6^-fold. The protein concentration was determined by measuring A_280_ and using the theoretical extinction coefficient (ε = 46910 M^–1^ cm^–1^), calculated using the ExPASy ProtParam tool. *Sm*AA10A used in the H_2_O_2_ production assay was purified as described previously.^34^

### H_2_O_2_ Production Assay

H_2_O_2_ production, i.e., the oxidase activity of the LPMO in the absence of carbohydrate-substrate, was measured as previously described.^23^ Stocks of Amplex Red Reagent (Thermo Fisher Scientific, Waltham, MA, USA) were prepared to 10 mM, dissolved in DMSO. Reactions were prepared in a 90 μL volume containing 50 mM Bis-Tris pH 6.5, 100 μM Amplex Red, 5 U/mL horseradish peroxide (HRP; Sigma-Aldrich, St. Louis, MO, USA), and 1 μM LPMO, and were preincubated at 30 °C for 5 min. Following preincubation, reactions were initiated with 10 μL of 10 mM ascorbic acid (AscA) (1 mM final concentration) and incubated at 30 °C. Formation of resorufin was monitored over 40 min at 540 nm in a Multiskan FC microplate photometer (Thermo Fisher Scientific, Waltham, MA, USA). A H_2_O_2_ standard curve was prepared in the same manner, with AscA added prior to the addition of Amplex Red/HRP. H_2_O_2_ concentrations were calculated after adjusting for side-reactions between AscA and Amplex Red. For comparison, a reaction with 1 μM Cu(II)SO_4_ instead of LPMO was included. Of note, while AscA concentrations of 1 mM were originally thought to not be compatible with the Amplex Red assay, it has been shown that they are, if adequate control reactions are included in the experiment.^24^

### Cellopentaose Degradation Assays

Standard LPMO reactions contained 2 μM LPMO, 1 mM cellopentaose (Megazyme, Bray, Ireland), and 50 mM Bis-Tris pH 6.5. Reactions were initiated by the addition of 1 mM AscA and incubated at 37 °C, 750 rpm in an Eppendorf Thermomixer (Hamburg, Germany). 50 μL samples were taken at various time points, and activity was stopped with the addition of 150 μL of 200 mM NaOH, followed by filtration through a 0.45 μM filter plate. In reactions containing catalase, the catalase was added prior to the addition of AscA. Catalase from bovine liver (Sigma-Aldrich, St. Louis, MO, USA) was dissolved in 50 mM Bis-Tris pH 6.5 at a stock concentration of 9000 U/mL and prepared fresh for every experiment. In reactions containing exogenous H_2_O_2_, 100–300 μM H_2_O_2_ was added to the reaction prior to the addition of AscA and a lower concentration of enzyme (100–250 nm) was used. The concentration of H_2_O_2_ in stock solutions was determined by measuring the absorbance at 240 nm and using an extinction coefficient of 43.6 M^–1^·cm^–1^. All reactions were performed under aerobic conditions.

### Product Analysis

Quantification of reaction products was done using high performance anion exchange chromatography with pulsed amperometric detection (HPAEC-PAD) as described previously.^82^ HPAEC-PAD was performed on a Dionex ICS5000 (Thermo Fisher Scientific, Waltham, MA, USA) equipped with a 3 Å 250 mm CarboPac PA200 analytical column and a CarboPac PA200 guard column. 5 μL samples were injected, and analytes were eluted using a 26 min stepwise gradient with an increasing amount of eluent B (eluent B: 0.1 M NaOH and 1 M NaOAc; eluent A: 0.1 M NaOH). The gradient consisted of 0–5.5% buffer B over 3 min, 5.5–15% buffer B over 6 min, 15–100% buffer B over 11 min, 100–0% over 6 s, and 0% buffer B over 6 min, using a flow rate of 0.5 mL/min. Chromatograms were recorded and analyzed using Chromeleon 7. When the activity on cellopentaose was assessed, only the amount of native cellotriose (the predominant product) was quantified for simplicity. Native cellotriose was used as a standard (Megazyme, Bray, Ireland).

### Determination of the Redox Potential

The cell potential of the LPMO-Cu(II)/LPMO-Cu(I) redox couple was determined from the reaction between reduced *N*,*N*,*N*′,*N*′-tetramethyl-1,4-phenylenediamine (TMP_red_) and LPMO-Cu(II). All reagents were prepared anaerobically and flushed with N_2_. Reactions contained a 60 μL final volume with a final concentration of 35 μM LPMO and 150 μM TMP_red_ in 20 mM Pipes buffer pH 6.0 and were incubated at room temperature for 5 min. The generated TMP_ox_ was measured anaerobically using a Nanophotometer C40 (Implen, München, Germany) at 610 nm. The cell potential of the LPMO-Cu(II)/LPMO-Cu(I) redox couple was determined from the concentration of TMP_ox_, as described previously.^35,57^ The reported values are shifted to be referenced against a normal hydrogen electrode (NHE).

### Fluorescence-Detected Stopped-Flow Spectroscopy

The intrinsic difference in fluorescence between the Cu(II) and Cu(I) states of the *Nc*AA9C variants (Figure S3) was used to measure the kinetics of LPMO reduction by AscA and reoxidation by H_2_O_2_. All of the experiments were carried out with a stopped-flow rapid spectrophotometer (SFM4000, BioLogic, Seyssient-Pariset, France) coupled to a photomultiplier with an applied voltage of 600 V for detection. The excitation wavelength was set to 280 nm, and the fluorescence increase (for reduction) or decay (for reoxidation) was monitored by using a 340 nm bandpass filter. The observation head was equipped with an FC-15/7.5 cuvette to maximize the fluorescence detected at a 90 °C angle. All experiments were carried out at 25 °C in 50 mM Bis-Tris at pH 6.5. To monitor the reduction of LPMO-Cu(II) to the Cu(I) state, single-mixing experiments were carried out. LPMO-Cu(II) (10 μM stock concentration, 5 μM final concentration) was mixed with different concentrations of AscA (ranging from 25–500 μM final concentrations). For reoxidation, double-mixing experiments were carried out. In a first step, the LPMO-Cu(II) (10 μM stock concentration, 2.5 μM final concentration) was mixed with a 1 mol equiv of l-cysteine for 15 s to form LPMO-Cu(I). In a second step, the *in situ* generated LPMO-Cu(I) was mixed with different concentrations of H_2_O_2_ (ranging from 5 to 100 μM final concentration) while the decay of fluorescence was recorded. All reactions were performed at least in triplicate. All reagents were deoxygenated by using a Schlenk line with N_2_ flux and prepared in sealed syringes in an anaerobic chamber. The stopped-flow rapid spectrophotometer was flushed with a large excess of anaerobic buffer before attaching the sealed syringes and performing the experiments.

### Stopped-Flow UV–vis Experiments

The formation of UV–vis features after reactivity with H_2_O_2_ was monitored using a similar setup to that described above for transient-state reoxidation kinetics. In this case, a TC-100/10 cuvette with a 1 cm path length was installed in the observation head and a TIDAS S 500 MCS UV/NIR 1910 (J&M Analytik AG, Essingen, Germany) diode array was used for the detector. The LPMO-Cu(II) (62.5–75 μM final concentration) was mixed with 1 molar equiv of AscA for 30 s to ensure LPMO-Cu(I) formation. The protein was then mixed with 40 mol equiv of H_2_O_2_, and the UV–vis traces were collected with 1.5 ms sampling between spectra. The reagents and the stopped-flow setup were prepared as described above. The experiments were performed in at least duplicate and were carried out at 4 °C in 50 mM Bis-Tris pH 6.5.

### Kinetics Data Analysis

The fluorescence data monitored with the stopped-flow was fitted to a single exponential function (*y* = *a*·*t* + *b* + *c*·*e*^–*k*_obs_·*t*^) using the BioKine32 V4.74.2 software (BioLogic, Seyssient-Pariset, France) to obtain the first order rate constant (*k*_obs_) for each AscA or H_2_O_2_ concentration. Plots of *k*_1obs_ versus AscA or *k*_2obs_ versus H_2_O_2_ concentration were fit using a linear least-squares regression to obtain the apparent second order rate constant of the reduction step (*k*_1app_^AscA^) or reoxidation step (*k*_2app_^H2O2^) using GraphPad Prism 9.

### EPR Spectroscopy

Protein solutions in 50 mM Bis-Tris at pH 6.5 were transferred into a quartz tube (4 mm diameter) and were frozen in liquid nitrogen. The continuous-wave X-band (∼9.63 GHz) EPR spectra were measured on a Bruker ELEXSYS E500 spectrometer (Billerica, MA, USA) equipped with a SuperX microwave unit, a Bruker dual-mode cavity (ER4116DM), and an Oxford ESR 900 liquid helium continuous-flow cryostat, which held the experimental cavity at a temperature of 30 K. Spectra were collected with a field modulation amplitude of 7.460 G at a frequency of 100 kHz and using a 20.48 ms time constant and 81.92 ms conversion time for the collection of a 1024-point spectrum for each scan. A total of 4 scans were collected for each sample. Sample conditions and experimental parameters are delineated in Table S1. All spectra were simulated in MATLAB 2021b with the EasySpin package (v 6.0.0-dev.43, release 2022-08-18),^83^ and simulated spin Hamiltonian parameters are listed in Table S1.

### X-ray Absorption Spectroscopy

Cu K-edge X-ray absorption data were measured at the I20-scanning beamline at Diamond Light Source (DLS; Oxfordshire, UK) (3 GeV, 300 mA). A four-bounce monochromator equipped with Si(111) crystals was utilized for upstream energy selection, and rhodium-coated mirrors were used for harmonic rejection, providing an unattenuated flux of ∼1 × 10^12^ photons/s at the sample position. The X-ray beam was focused to an approximate beam spot size of 0.3 × 0.4 mm^2^ (*v* × *h*; full width at half-maximum (fwhm)). Samples of WT *Nc*AA9C and the Q164E mutant were prepared in 50 mM MES pH 6.5 with 30% (v/v) glycerol. The final LPMO concentration for each sample was determined by UV–vis absorption at 280 nm (ε = 46910 M^–1^ cm^–1^), to be 0.6 mM for WT *Nc*AA9C and 0.8 mM for Q164E. Each solution was transferred into a Delrin cell with a Kapton tape window, followed by freezing and storage in liquid nitrogen until the measurement was performed. During the measurement, the sample temperature was maintained at 10 K using a top loading exchange gas pulse tube He cryostat to minimize photodamage.

All data were collected by scanning the incident energy from 8860 to 9641 eV, and calibrated by simultaneous measurement of a Cu foil, for which the first inflection point of the Cu foil was set to 8980.3 eV. Three ionization chambers were positioned before the sample (*I*_0_), after the sample (*I*_t_), and after a reference foil (*I*_ref_), and fluorescence data from the sample were recorded by a 64-element monolithic Ge detector, with readout performed by the Xspress4 digital pulse processor. Prior to the collection of the full spectra, a series of short, low-resolution scans at the near-edge region were collected to identify X-ray-induced photodamage, revealing the emergence of a peak at 8983.5 eV upon continued exposure to the beam (Figure S8). Therefore, the acquisition of undamaged spectra was achieved by attenuation of the incident beam to ∼4 × 10^9^ photons/s, limited to a 12 min scan duration for each scan, and translation to a fresh sample spot between each scan.

Data processing was performed in the Athena module of the Demeter software package,^84^ as previously described.^85^ The data were truncated to *k* = 12 Å^–1^, and background subtraction and normalization of the data were performed using a linear regression for the pre-edge region (8860–8962 eV) and a cubic polynomial regression for the postedge region of 9143–9539 eV. Data were splined along the *k* = 0–12 Å^–1^ range using an R-background of 1.0 and *k*-weight of 3. EXAFS modeling and fitting were performed by using the Artemis module within Demeter. The EXAFS was *k*^3^-weighted to enhance the impact of high-*k* data, and Fourier transformed over a Hanning-windowed *k* = 2–12 Å^–1^ range. Scattering paths were calculated using FEFF6 and fit to the FT data over a range of *R* = 1.0–4.0 Å (nonphase shift corrected). Similar scattering paths were grouped together as degenerate paths when the path lengths were within the Δ*R* = 0.167 Å resolution. A single E_0_ variable was applied for all paths in a given fit. The amplitude reduction factor (S_0_^2^) was fixed at 0.9 for all paths. An initial fit single-scattering path was first performed (fits denoted “SS” in Tables S3 and S4) for each set of degenerate paths, with each path assigned a unique σ^2^ and *R* variable. Paths featuring long-range single or multiple scattering atoms were then subsequently determined (fits denoted “MS”), appending reasonable fits obtained from the initial fitting of proximal shells. For each multiple-scattering path, the path distance (as a function of *R*) was allowed to vary, while σ^2^ was defined by multiplying the σ^2^ of the single-scattering path of the more proximal scatterer by a factor of 1.5, in order to avoid the introduction of weak variables into the fit. Information about selected fits is shown in Figures S6, S7, and S8 and Tables S3 and S4.

### Computational Methods

#### Protein Preparation

The small model of the *Nc*AA9C active site (Chain A) used for DFT calculations was constructed from the crystal structure of the Cu(II) state of the protein (PDB entry 4D7U,^36^ resolution 1.56 Å). The protonation states of titratable residues included in the cluster model (His, Glu) were assigned on the basis of p*K*_a_ values at pH 6.5, using the PROPKA 3 software^86,87^ in combination with a careful visual inspection of local hydrogen-bonding networks. For the Q164E mutant, the Gln164 position was mutated to Glu164 using the PyMOL mutagenesis wizard.^88^ The backbone dependent rotamer with the least structural deviation, low clashes, and appropriate side-chain positioning (similar to Gln164) was determined as a suitable starting point. Glu164 was selectively protonated to assess all possible protonation states of the residue.

#### Cluster Model

The cluster model consisted of Cu, His1, His83, Tyr166, Gln164, and His155. His1 was truncated at the α carbon, and the carboxylic acid group was replaced by hydrogen, while the N-terminal amino group was unchanged. All other residues were truncated at the α carbon and substituted with methyl groups. To maintain the constraints imposed on the active site by the full protein, including bonds to the backbone and strong hydrogen bonds, several atoms were fixed in position by using a fragment constraint protocol during optimizations. Each amino acid and Cu were considered as a separate fragment, and fragments were connected through the following atoms: the β carbon of His1, the α carbon of His83, the α carbon of Tyr166, the α carbon of His155, and the α carbon of Gln164 and Cu. This cluster model was optimized in the Cu(II) state with two H_2_O in the equatorial and axial ligand positions that are not occupied by amino acids. Optimization in the Cu(I) state was done after removal of the H_2_O molecules.^89^ The coordinates of the water molecules were taken from *Ls*AA9A (PDB entry 7PXS(90)). The H_2_O_2_ molecule was placed in the position of the equatorial water and aligned such as to interact with His155 and Gln/Glu164 based on the results of previous computational studies.^16,17,43^ Multiple conformations of the H_2_O_2_ molecule were tested to identify the lowest energy conformer, which was considered for reactivity calculations.

#### Calculations

All calculations were performed using the ORCA 5.0.3 software package.^91−93^ Geometry optimizations were performed with the B3LYP^94,95^ functional using the def2-TZVPP^96^ basis set for Cu, while using the def2-SVP^96^ basis set for all other atoms in the cluster model. The def2/J^97^ basis sets along with chain of spheres approximation (RIJCOSX)^98^ were used for the RI (resolution of identity) approximation to the Coulomb integrals. The calculations were performed using water implicit solvation as per the CPCM^99^ approach and using atom-pairwise dispersion correction based on tight binding partial charges (D4).^100,101^ The reactivity profile was investigated with relaxed surface scan unrestricted calculations varying the O–O bond distance in H_2_O_2_ from 1.2 to 3.0 Å in 0.1 Å steps in the ground state and from 3.0 to 1.0 Å in the high spin state. The same was performed for the broken-symmetry singlet surface.

The local and global minimum states and transition states identified with the small basis set were further optimized considering relativistic effects using ZORA,^102^ with the ZORA-def2-TZVPP basis set for Cu, while using ZORA-def2-TZVP for the rest of the atoms in the cluster.^103^ The SARC/J basis sets were used for the RI approximation to the Coulomb integrals.^103−106^ For optimizations, TightOpt settings were used, while for frequency calculations, the convergence criteria were increased to match those of the “TightOpt” setting. The integration grid used was the default (Def2grid).

## Supporting Information Available

Additional biochemical characterizations, experimental details for EPR and EXAFS, EXAFS fittings and additional computational information including optimized coordinates. (PDF)
