# Supplementary material for: A Conserved Second Sphere Residue Tunes Copper Site Reactivity in Lytic Polysaccharide Monooxygenases
Source: J Am Chem Soc. 2023 Aug 16;145(34):18888–903. doi: 10.1021/jacs.3c05342 (PMC10472438; doi:10.1021/jacs.3c05342)
Supplement: Supplementary file 1 — ja3c05342_si_001.pdf [file ja3c05342_si_001.pdf]

Supplementary Information for

A conserved second sphere residue tunes copper site reactivity in lytic polysaccharide monooxygenases

Kelsi R. Hall[1], Chris Joseph[2], Iván Ayuso-Fernández[1], Ashish Tamhankar[2], Lukas Rieder[3], Rannei Skaali[1], Ole Golten[1], Frank Neese[4], Åsmund K. Røhr[1], Sergio A. V. Jannuzzi[2], Serena DeBeer[2*], Vincent G. H. Eijsink[1*] and Morten Sørlie[1*]

[1] Faculty of Chemistry, Biotechnology and Food Science, Norwegian University of Life Sciences (NMBU), 1432, Ås, Norway.
[2] Max Planck Institute for Chemical Energy Conversion, Stiftstraße 34-36, 45470 Mülheim an der Ruhr, Germany.
[3] Institute for Molecular Biotechnology, Graz University of Technology, 8010, Graz, Austria.
[4] Max-Planck-Institut für Kohlenforschung, Kaiser-Wilhelm-Platz 1, 45470 Mülheim an der Ruhr, Germany.

*Correspondence to: morten.sorlie@nmbu.no, vincent.eijsink@nmbu.no, serena.debeer@cec.mpg.de

This PDF file includes:

1. List of Supplementary Figures & Tables pg. 2
2. Supplementary Figures (1-15) and Tables (1-5) pg. 2-23
3. Supplementary References pg. 23
4. Sample Input for Geometry Optimization pg. 24-25
5. Optimized Coordinates for WT *Nc*AA9C pg. 26-37
6. Optimized Coordinates for the Q164E mutant (glutamate form) pg. 38-49
7. Optimized Coordinates for the Q164E mutant (glutamic acid form) pg. 50-57

1. List of Supplementary Figures and Tables

Figure S1. Enzyme inactivation in the Q164E mutant.

Figure S2. Product formation by the Q164E mutant with various H_2O_2 and AscA concentrations.

Figure S3. Change in fluorescence signal upon reduction of *Nc*AA9C variants.

Figure S4. Formation of a tryptophanyl radical in WT *Nc*AA9C reacting with H_2O_2.

Figure S5. Frozen solution X-band EPR spectra (30 K) of the four *Nc*AA9C variants (WT, Q164N, Q164E, and Q164D.

Table S1. Spin Hamiltonian parameters for the *Nc*AA9C variants.

Table S2. EPR experimental details for the *Nc*AA9C variants.

Figure S6. Atom labels used for multiple scattering paths in Tables S3 and S4

Table S3. Selected EXAFS fitting parameters for WT *Nc*AA9C-Cu(II).

Table S4. Selected EXAFS fitting parameters for the Q164E-Cu(II) mutant

Figure S7. Selected EXAFS fits for WT *Nc*AA9C-Cu(II).

Figure S8. Selected EXAFS fits for the Q164E-Cu(II) mutant.

Figure S9. XAS scans of the Q164E mutant.

Further remarks on EXAFS fits.

Table S5. Key parameters of optimized structures along the H_2O_2 activation pathway.

Figure S9. Optimized geometries by DFT for WT *Nc*AA9C.

Figure S11. Spin density plots for intermediate complexes for the reaction with WT *Nc*AA9C.

Figure S12. Optimized geometries by DFT for the Q164E in the glutamate form.

Figure S13. Spin density plots for intermediate complexes for the reaction with the Q164E mutant in the glutamate form.

Figure S14. Optimized geometries by DFT for the Q164E mutant in the glutamic acid form.

Figure S15. Spin density plots for intermediate complexes for the reaction with the Q164E mutant in the glutamic acid form.

2. Supplementary Figures and Tables

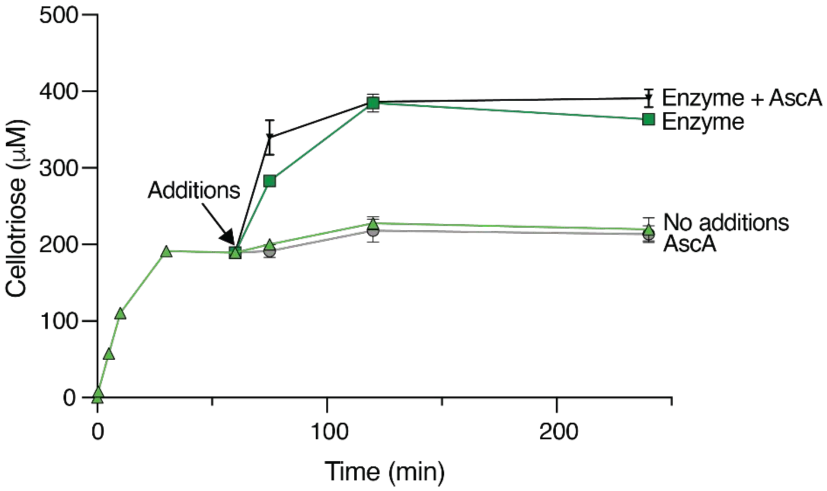

Figure S1. Enzyme inactivation in the Q164E mutant. Reactions containing 2 µM Q164E and 1 mM AscA were incubated for 60 minutes in 50 mM Bis-Tris pH 6.5 at 37 °C. The reaction was then split into four reactions and (1) enzyme, (2) AscA, (3) enzyme and AscA or (4) buffer (control) were added to these four reactions in amounts identical to those added at t = 0. The graph shows that product formation resumed only in reactions supplied with fresh enzyme.

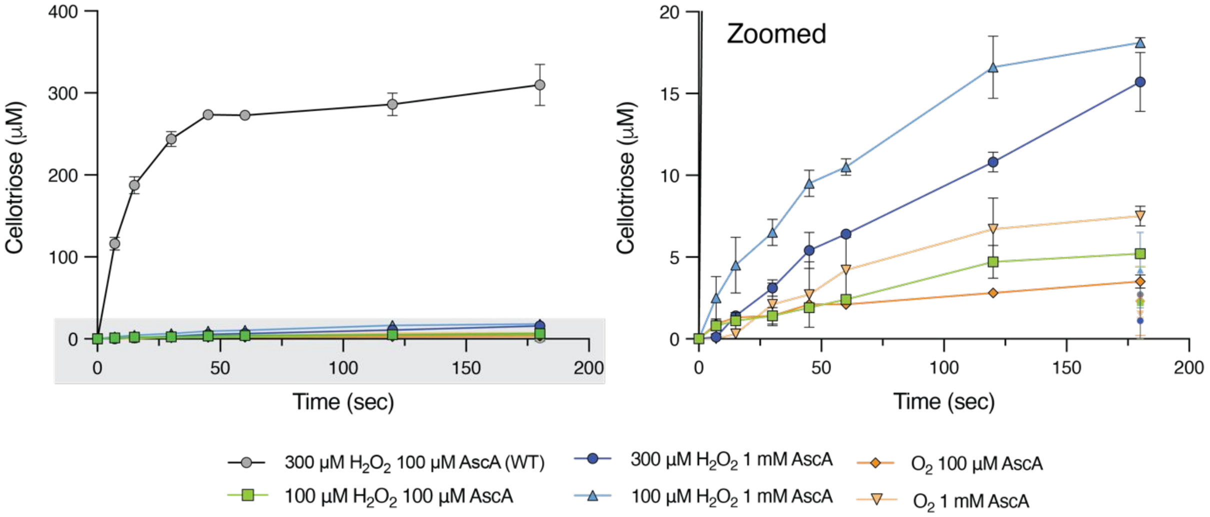

Figure S2. Product formation by the Q164E mutant with various H_2O_2 and AscA concentrations. Reactions contained 250 nM Q164E, 1 mM Glc_5, 50 mM Bis-Tris pH 6.5, 100 or 300 µM H_2O_2 and/or 100 or 1000 µM AscA, as indicated in the Figure and were incubated at 37 °C. The right-hand image shows a zoomed in view of the image depicted on the left, due to the scale the WT control reaction (grey circles in the left-hand image) is not visible. For all experiments, error bars show ± SD (n = 3; independent experiments). Product levels after 180 s in control reactions containing no AscA are shown as individual points with all negative controls showing less product than the corresponding reactions at 180 s. It is worth noting that while the reaction with Q164E and a low concentration of AscA (100 µM) plateaued after 5 µM of product was produced, reactions with a high concentration of AscA (1 mM) proceeded for a longer time and produced more product. This suggests that at the lower concentrations, AscA is limiting the activity of the Q164E mutant. It should also be noted that, while high concentrations of AscA (1 mM) did result in faster, and more prolonged release of product, the approximate rate of this reaction was still much slower compared to rates observed under similar conditions for WT *Nc*AA9C, with the fastest initial rate (k_{obs}) measured for Q164E being only 0.71 ± 0.09 s^{-1} (see main text for further discussion).

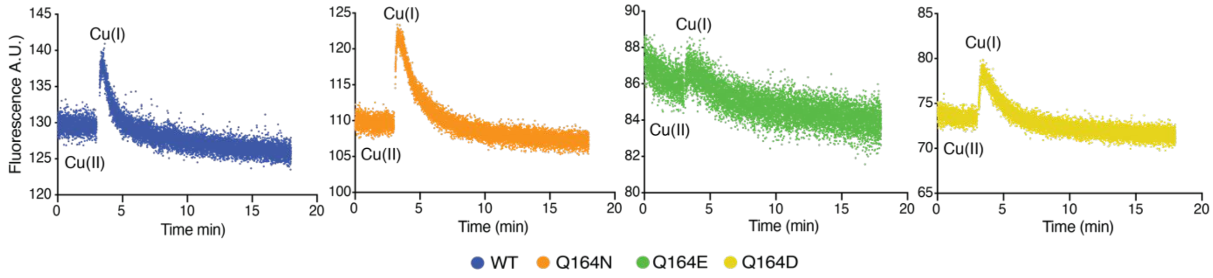

Figure S3. Change in fluorescence signal upon reduction of *Nc*AA9C variants. The reactions contained 2 µM LPMO in the Cu(II) state (blue, WT; orange, Q164N; green, Q164E; yellow, Q164D) in 50 mM Bis-Tris pH 6.5. Reactions were added to 2 mL quartz cuvettes and monitored at ex 280/em 343 nm for 3 minutes to establish the background fluorescence. One molar equivalent of AscA (2 µM) was then added to the cuvette to generate the Cu(I) form of the enzyme.

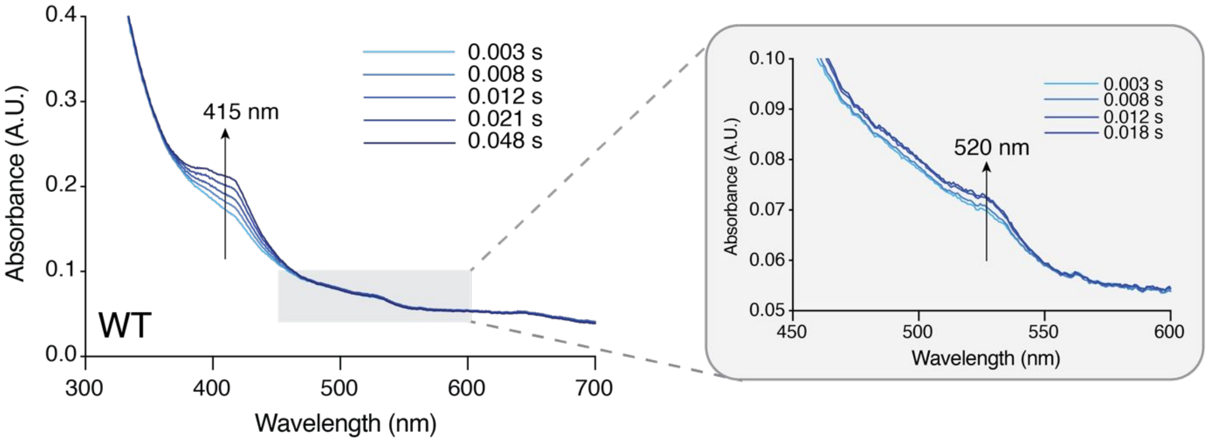

Figure S4. Formation of a tryptophanyl radical in WT *Nc*AA9C reacting with H_2O_2. WT *Nc*AA9C-Cu(II) was mixed anaerobically with a 1-molar equivalent of AscA to generate *Nc*AA9C-Cu(I). Stopped-flow transients were then measured in the absence of substrate at 4 °C by reacting reduced enzyme with a 40-molar excess of H_2O_2. Formation of the ≈ 415 nm spectral feature is shown on the left and formation of the ≈ 520 nm spectral feature is shown in the grey inset.

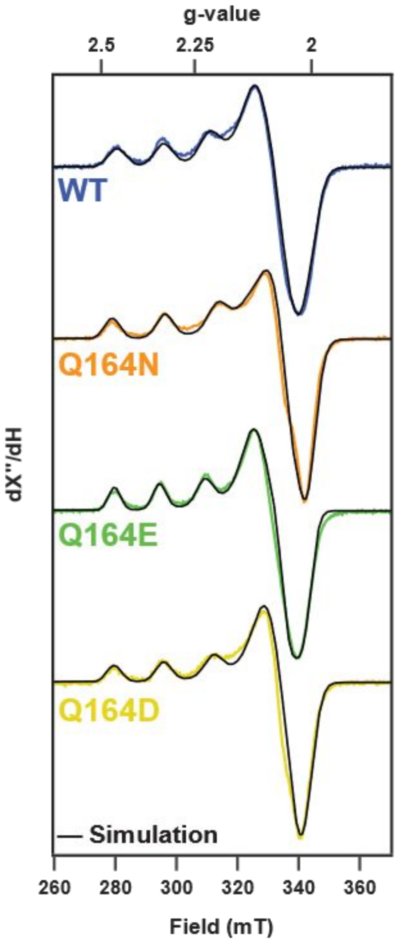

Figure S5. Frozen solution X-band EPR spectra (30 K) of the four *Nc*AA9C variants (WT, Q164N, Q164E, and Q164D). Colored traces depict the experimental data for the indicated *Nc*AA9C variant. Black traces depict simulations using spin Hamiltonian parameters delineated in Table S1. Spectra were collected on protein samples in 50 mM Bis-Tris pH 6.5. Experimental conditions and parameters are shown in Table S2.

All four variants show spectra indicative of a strongly axial type 2 copper site, consistent with previously described EPR behavior of AA9s[1], including WT *Nc*AA9C.[2] The spin Hamiltonian parameters (Table S1) in the parallel direction could be accurately simulated, and applying the Peisach-Blumberg classification to the simulated g_z and $|A_z|$ parameters places the *Nc*AA9C Cu sites within the typical values for type-2 copper sites.[3]

Table S1. Spin Hamiltonian parameters for the *Nc*AA9C variants. The parameters were determined by simulation in MATLAB 2021b with the EasySpin package (v 6.0.0-dev.43, release 2022-08-18).[4]

		**g**	**A_{Cu} (× 10^{-4} cm^{-1})**
*Nc*AA9C	WT	[2.033, 2.097, 2.270]	[11, 20, 157]
	Q164N	[2.045, 2.079, 2.258]	[11, 27, 178]
	Q164E	[2.037, 2.091, 2.280]	[17, 22, 154]
	Q164D	[2.047, 2.070, 2.265]	[11, 20, 168]

Table S2. EPR experimental details for the *Nc*AA9C variants.

	*Nc*AA9C			
	**WT**	**Q164N**	**Q164E**	**Q164D**
LPMO Concentration [a]	617 µM	492 µM	559 µM	279 µM
Microwave Freq (GHz)	9.634	9.634	9.632	9.635
Power (mW)	0.02002	0.1003	0.05029	0.05029
Receiver gain (dB)	60	60	60	60
Center field (mT)	350	350	350	350
Sweep width (mT)	250	250	250	250
Modulation amplitude (mT)	0.7460	0. 7460	0. 7460	0. 7460
Modulation Frequency (kHz)	100.00	100.00	100.00	100.00
Number of points	1024	1024	1024	1024
Conversion time (ms)	81.92	81.92	81.92	81.92
Filter time constant (ms)	20.48	20.48	20.48	20.48
Temperature (K)	30	30	30	30

[a] concentration determined by UV-vis absorbance at 280 nm; $\varepsilon = 46910\ M^{-1}cm^{-1}$.

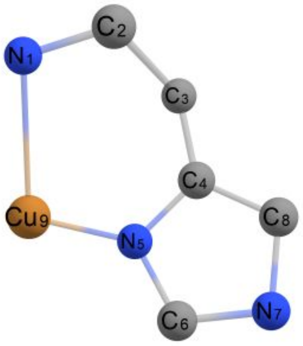

Figure S6. Atom labels used for multiple scattering paths in Tables S3 and S4. Scattering paths were calculated using FEFF6 and fit to the FT-EXAFS data. Considering the ΔR = 0.167 Å resolution, similar scattering paths were grouped together as degenerate paths when the path lengths were within the limits of resolution. For example, paths composed of the atoms used for the histidine ligand (C4, N5, C6, N7, C8) were assigned a degeneracy of N=2 to account for a second histidine ligand.

Table S3. Selected EXAFS fitting parameters for WT *Nc*AA9C-Cu(II). *N* is the path degeneracy, *R* is half the scattering path distance (*i.e.* the distance between the absorber and scatterer in the single-scatterer paths), σ^2 is the Debye-Waller factor, and E_0 is the origin of the photoelectron wave vector. Atom labels for multiple-scattering pathways are illustrated in Figure S6. Selected fits are depicted in Figure S7.

Fit	Path	N	R (Å)	σ^2 ($\times 10^{-3}$ Å^2)	E_0 (eV)	red χ^2
**SS1**	Cu-N/O	5	2.01	5.83	8991.6	8.85
	Cu-N/O	1	2.31	3.93		
**SS2**	Cu-N/O	4	2.01	3.83	8992.5	9.15
	Cu-N/O	1	2.31	9.05		
**SS3**	Cu-N/O	4	2.00	3.88	8990.5	8.98
**MS1**	Cu-N/O	5	2.01	5.56	8992.2	3.78
	Cu-N/O	1	2.30	4.30		
	[His] Cu-$C_{2/4/6}$	5	2.99	6.05		
	[His] Cu···$N_{1/5}$···$C_{2/4/6}$	10	3.15	8.34[a]		
	[His] Cu···N_5···N_7/C_8	8	4.24	8.34[a]		
**MS2**	Cu-N/O	4	2.01	3.64	8992.8	3.79
	Cu-N/O	1	2.29	9.61		
	[His] Cu-$C_{2/4/6}$	5	2.99	4.99		
	[His] Cu···$N_{1/5}$···$C_{2/4/6}$	10	3.15	5.46[a]		
	[His] Cu···N_5···N_7/C_8	8	4.24	5.46[a]		
**MS3**	Cu-N/O	4	2.00	3.81	8990.7	4.42
	[His] Cu-$C_{2/4/6}$	5	2.98	5.04		
	[His] Cu···$N_{1/5}$···$C_{2/4/6}$	10	3.14	5.72[a]		
	[His] Cu···N_5···N_7/C_8	8	4.21	5.72[a]		
**MS4**	Cu-N/O	5	2.01	5.56	8992.0	3.92
	Cu-N/O	1	2.30	4.39		
	[His] Cu-$C_{4/6}$	4	2.99	4.88		
	[His] Cu···N_5···$C_{4/6}$	8	3.15	8.34[a]		
	[His] Cu···N_5···N_7/C_8	8	4.23	8.34[a]		
**MS5**	Cu-N/O	4	2.01	3.64	8992.5	3.84
	Cu-N/O	1	2.29	9.95		
	[His] Cu-$C_{4/6}$	4	2.99	3.93		
	[His] Cu···N_5···$C_{4/6}$	8	3.15	5.46[a]		
	[His] Cu···N_5···N_7/C_8	8	4.23	5.46[a]		
**MS6**	Cu-N/O	5	2.00	5.63	8989.8	6.19
	Cu-N/O	1	2.31	4.06		
	[Tyr] Cu-C	1	2.98	–2.06		
	[His] Cu···$N_{1/5}$···$C_{2/4/6}$	10	3.19	8.45[a]		
	[His] Cu···N_5···N_7/C_8	8	4.21	8.45[a]		

[a] σ^2 is defined as 1.5 times that of the first Cu-N/O single scattering path ($1.5 \times \sigma^2_{Cu\text{-}N/O}$).

Table S4. Selected EXAFS fitting parameters for the Q164E-Cu(II) mutant. *N* is the path degeneracy, *R* is half the scattering path distance (*i.e.* the distance between the absorber and scatterer in the single-scatterer paths), σ^2 is the Debye-Waller factor, and E_0 is the origin of the photoelectron wave vector. Atom labels for multiple-scattering pathways are illustrated in Figure S6. Selected fits are depicted in Figure S8.

Fit	Path	N	R (Å)	σ^2 ($\times 10^{-3}$ Å^2)	E_0 (eV)	red χ^2
SS1	Cu-N/O	5	2.00	5.51	8991.1	21.8
	Cu-N/O	1	2.27	4.76		
SS2	Cu-N/O	4	2.00	3.58	8991.6	20.3
	Cu-N/O	1	2.26	10.49		
SS3	Cu-N/O	4	1.99	3.77	8989.3	19.7
MS1	Cu-N/O	5	2.00	5.16	8992.1	10.1
	Cu-N/O	1	2.26	3.82		
	[His] Cu-$C_{2/4/6}$	5	2.99	6.43		
	[His] Cu···$N_{1/5}$···$C_{2/4/6}$	10	3.15	7.74		
	[His] Cu···N_5···N_7/C_8	8	4.24	7.74		
MS2	Cu-N/O	4	2.00	3.27	8992.5	6.65
	Cu-N/O	1	2.24	7.48		
	[His] Cu-$C_{2/4/6}$	5	3.00	5.49		
	[His] Cu···$N_{1/5}$···$C_{2/4/6}$	10	3.16	4.91		
	[His] Cu···N_5···N_7/C_8	8	4.24	4.91		
MS3	Cu-N/O	4	1.99	3.56	8989.2	9.64
	[His] Cu-$C_{2/4/6}$	5	2.99	6.79		
	[His] Cu···$N_{1/5}$···$C_{2/4/6}$	10	3.18	5.34		
	[His] Cu···N_5···N_7/C_8	8	4.21	5.34		
MS4	Cu-N/O	5	2.00	5.16	8991.9	10.4
	Cu-N/O	1	2.26	3.97		
	[His] Cu-$C_{4/6}$	4	2.99	5.41		
	[His] Cu···N_5···$C_{4/6}$	8	3.15	7.73		
	[His] Cu···N_5···N_7/C_8	8	4.24	7.73		
MS5	Cu-N/O	4	2.00	3.27	8992.2	6.68
	Cu-N/O	1	2.24	7.96		
	[His] Cu-$C_{4/6}$	4	3.00	4.60		
	[His] Cu···N_5···$C_{4/6}$	8	3.16	4.90		
	[His] Cu···N_5···N_7/C_8	8	4.24	4.90		
MS6	Cu-N/O	4	2.00	3.29	8992.4	7.33
	Cu-N/O	1	2.24	8.18		
	[His] Cu-$C_{4/6}$	4	2.95	5.28		
	[His] Cu-C_2	1	3.05	–1.58		
	[His] Cu···$N_{1/5}$···$C_{2/4/6}$	8	3.13	4.94		
	[His] Cu···N_5···N_7/C_8	8	4.24	4.94		

[a] σ^2 is defined as 1.5 times that of the first Cu-N/O single scattering path ($1.5 \times \sigma^2_{Cu\text{-}N/O}$).

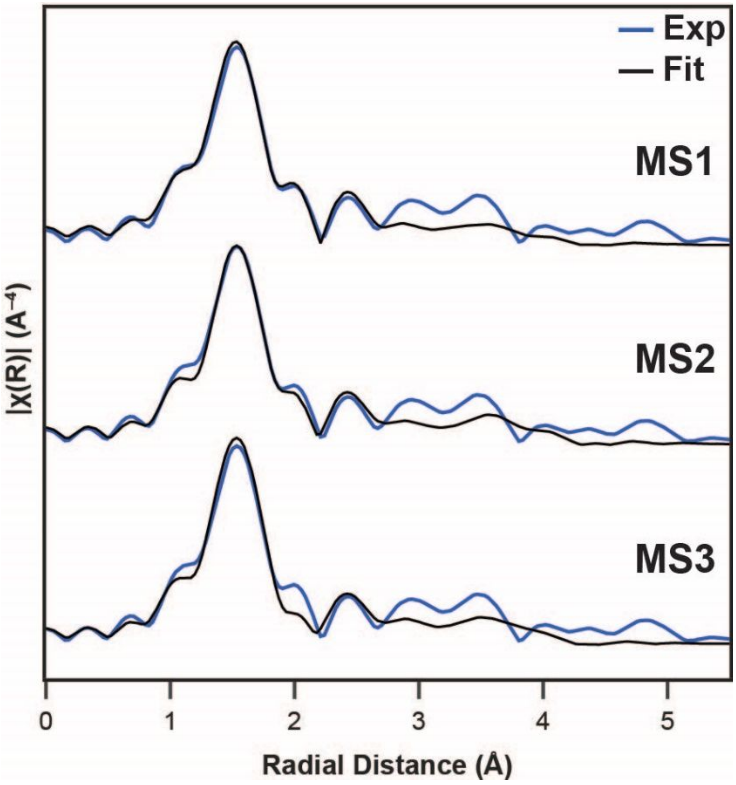

Figure S7. Selected EXAFS fits for WT *Nc*AA9C-Cu(II). Fits (with multiple-scatterer paths) demonstrating three cases derived from first fits: 6-coordinate, 5-coordinate, and 4-coordinate Cu site (MS1, MS2, and MS3, respectively). Fitting parameters are delineated in Table S3.

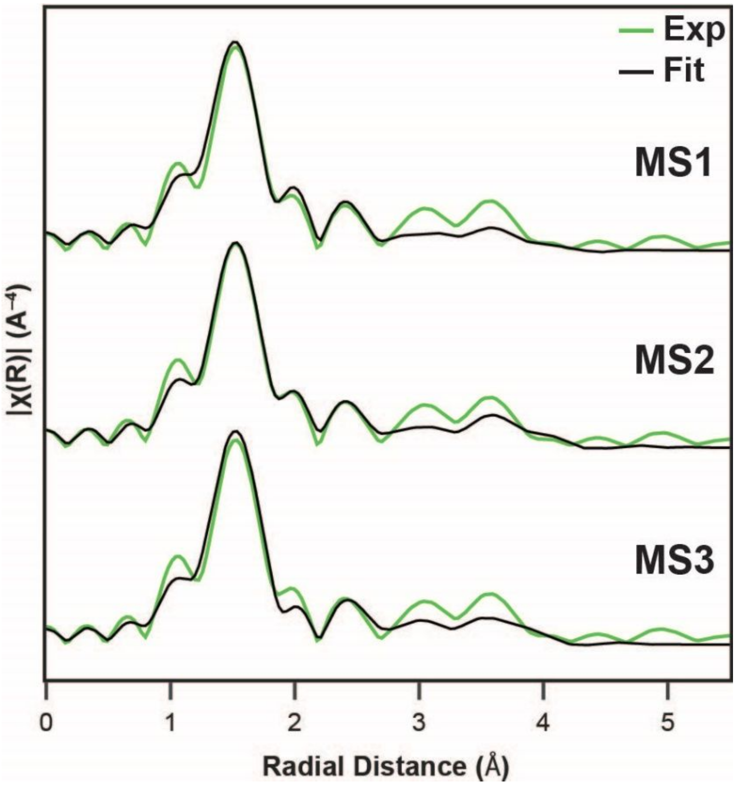

Figure S8. Selected EXAFS fits for the Q164E-Cu(II) mutant. Fits (with multiple-scatterer paths) demonstrating three cases derived from first fits: 6-coordinate, 5-coordinate, and 4-coordinate Cu site (MS1, MS2, and MS3, respectively). Fitting parameters are delineated in Table S4.

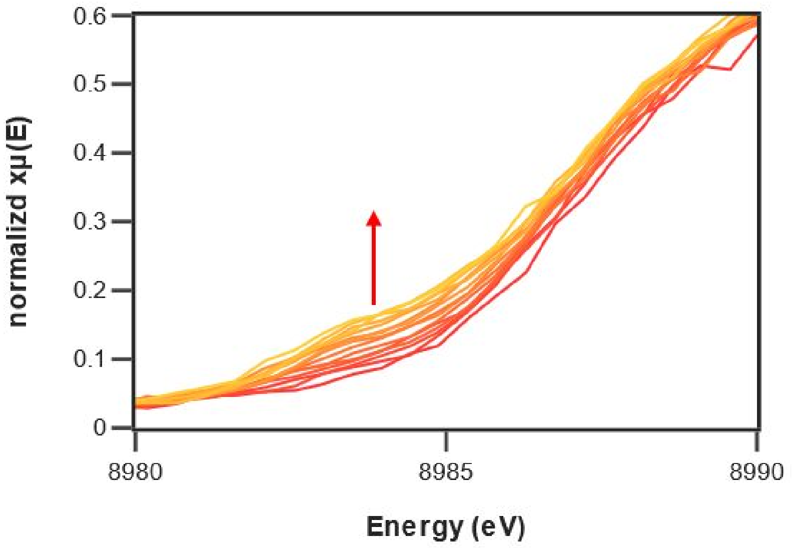

Figure S9. XAS scans of the Q164E mutant. A series of short, edge-region XAS scans obtained during damage assessment of *Nc*AA9C Q164E, revealing the emergence of a photoinduced feature at 8983.5 eV, attributed to Cu(II)→Cu(I) reduction.

Further remarks on EXAFS fits. The distances are generally in agreement with crystal structure data for AA9s, in which histidine imidazole distances are 1.9–2.0 Å from Cu while the terminal amine N resides 2.2–2.3 Å away from the Cu site. Interestingly, a previous report in which EXAFS data for an AA9 from *T. aurantiacus* were modeled found that the Cu(II) site was best fit with a 4-coordinate site[5]. However, attempts to model the *Nc*AA9C data like this led to unsatisfactory fits, in which a second-shell feature at 2 Å in the FT-EXAFS was not well-represented in the fit (Fit MS3 in Figures S7 and S8). Fitting of the long-distance data necessitated the inclusion of multiscattering paths from two His ligands. For the WT *Nc*AA9C data, MS1 and MS2 (Table S3) provided the best fits, though MS2 (the "5-coordinate" fit) yields a Debye-Waller factor which is unusually high for a path of singular degeneracy. In the case of the Q164E mutant, while the MS2 model provides the superior fit (Table S4), the Debye-Waller factor obtained for the second shell single scattering path is still notably high, though within an acceptable range. It was found that the best fits were those in which the His1 α-C (labeled C_2 in Figure S6) was grouped with the proximal C scatterers of the imidazole rings. Attempts to lower the degeneracy of the path (omitting the α-C) resulted in a nearly equivalent fit. On the other hand, including a unique path for the α-C either resulted in path lengths that could not be resolved within the resolution of the data (ΔR = 0.167 Å) or led to non-physically relevant fitting parameters. Finally, attempts to include longer range scatterers from Tyr (using models in which Cu–O_{Tyr} distances were assumed to be either 2.0 or 2.3 Å) did not provide any satisfactory fits.

Table S5. Key parameters of optimized structures along the H_2O_2 activation pathway. The values apply to calculations for the cluster models of WT *Nc*AA9C and the Q164E mutant with E164 negatively charged (Q164E(–)) or protonated (Q164E(0)), at the B3LYP/ZORA-def2-TZVP level of theory.

Parameter	Model	^{1}RC	^{1}TS	^{1}IC1	^{3}IC1	^{1}IC2	^{3}IC2
O1-O2 (Å)[a]	WT	1.462	1.681	2.183	2.225	2.635	2.640
	Q164E(–)	1.458	1.743	2.243	2.278	2.740	2.735
	Q164E(0)	1.454	1.681	2.776	2.748		
Cu-O1 (Å)	WT	3.085	2.267	1.955	1.975	1.897	1.899
	Q164E(–)	3.171	2.202	1.951	1.953	1.897	1.895
	Q164E(0)	3.178	2.281	1.808	1.918		
H1-O1 (Å)	WT	0.996	0.990	0.977	0.965	1.637	1.644
	Q164E(–)	0.996	0.995	0.980	0.983	1.766	1.758
	Q164E(0)	0.990	0.986	0.998	1.541		
H2-O2 (Å)	WT	0.970	0.967	0.966	0.968	0.965	0.965
	Q164E(–)	1.009	0.992	0.988	0.982	0.992	0.993
	Q164E(0)	0.972	0.968	0.972	0.978		
Cu-O1-O2 (°)	WT	149.3	175.9	172.2	113.1	119.7	127.9
	Q164E(–)	130.1	175.0	171.3	148.4	122.5	128.0
	Q164E(0)	154.2	176.0	177.1	168.3		
H1-O1-O2-H2 dihedral (°)	WT	139.9	144.5	163.0	65.7	-	-
	Q164E(–)	43.5	27.3	31.4	41.7	-	-
	Q164E(0)	100.8	136.1	-	-		
Cu spin pop.[b]	WT	0.000	-0.141	-0.552	0.674	-0.595	0.666
	Q164E(–)	0.000	0.224	0.573	0.667	-0.603	0.661
	Q164E(0)	0.000	0.000	0.000	0.679		
O1 spin pop. [b]	WT	0.000	0.027	0.194	0.476	0.729	1.057
	Q164E(–)	0.000	-0.016	-0.101	0.433	0.745	1.070
	Q164E(0)	0.000	0.000	0.000	1.012		
O2 spin pop. [b]	WT	0.000	0.141	0.524	0.597	0.003	0.004
	Q164E(–)	0.000	-0.253	-0.634	0.657	0.002	0.004
	Q164E(0)	0.000	0.000	0.000	0.006		
Cu charge pop. [b]	WT	0.003	0.106	0.288	0.287	0.258	0.248
	Q164E(–)	-0.009	0.122	0.267	0.271	0.246	0.236
	Q164E(0)	0.006	0.105	0.364	0.273		
O1 charge pop. [b]	WT	-0.135	-0.163	-0.293	-0.275	-0.296	-0.257
	Q164E(–)	-0.166	-0.209	-0.342	-0.325	-0.306	-0.275
	Q164E(0)	-0.122	-0.151	-0.270	-0.175		
O2 charge pop. [b]	WT	-0.136	-0.187	-0.277	-0.253	-0.243	-0.242
	Q164E(–)	-0.238	-0.302	-0.354	-0.337	-0.350	-0.354
	Q164E(0)	-0.102	-0.162	-0.329	-0.326		

[a] Involving the H_2O_2 oxygen atoms, in which O1 is the atom closer to the copper atom.
[b] Hirshfeld populations.

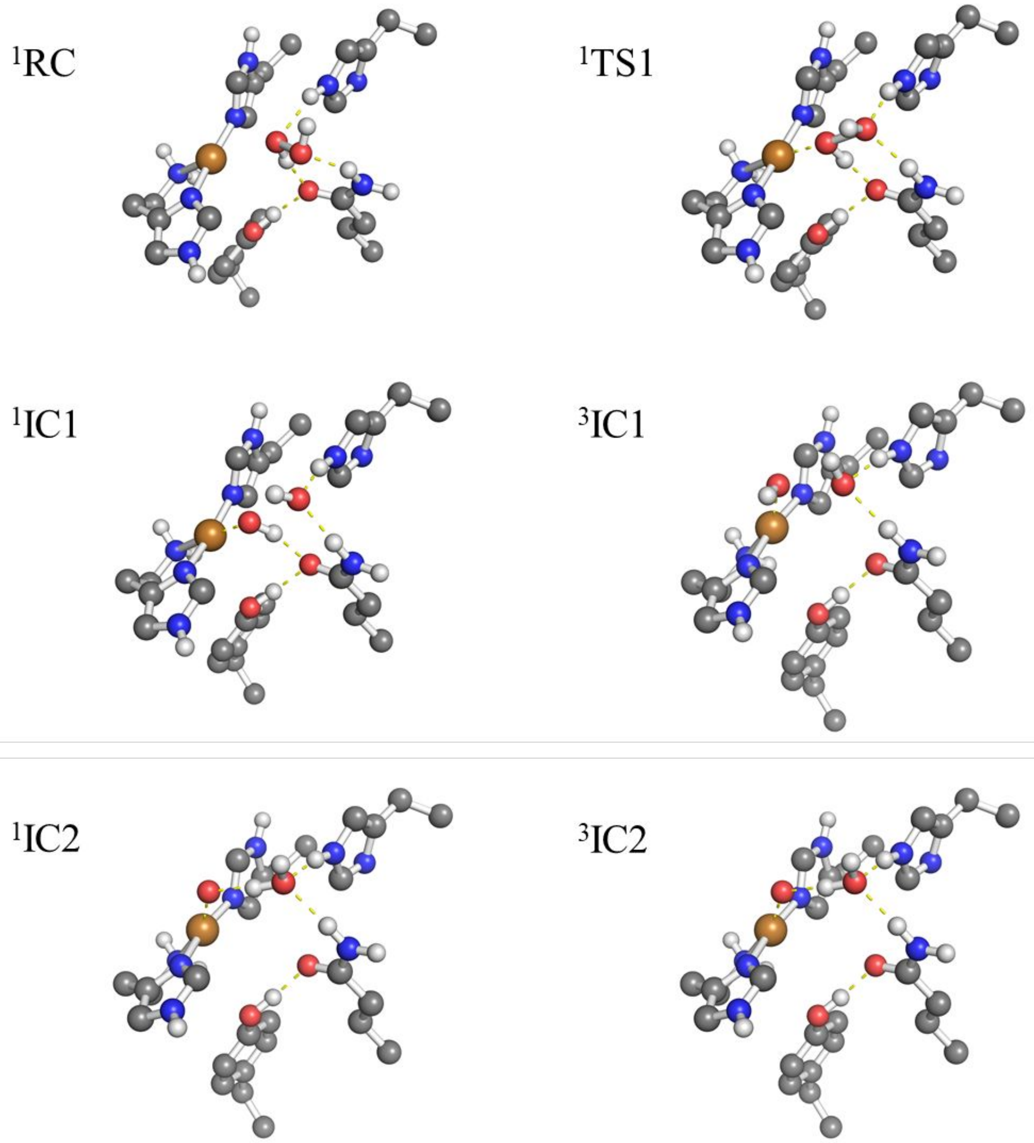

Figure S10. Optimized geometries by DFT for WT *Nc*AA9C. Optimized geometries are shown for the reactant complex (RC) $Cu^{I}+H_2O_2$, the transition state of the O-O homolytic cleavage (TS) and the intermediate complexes of the Cu(II)OH + $OH^{\bullet}$ step (IC1) and Cu(II)O• + H_2O (IC2) step found along the H_2O_2 splitting path of the reaction with WT *Nc*AA9C in the Cu(I) state. The left superscript indicates the spin multiplicity.

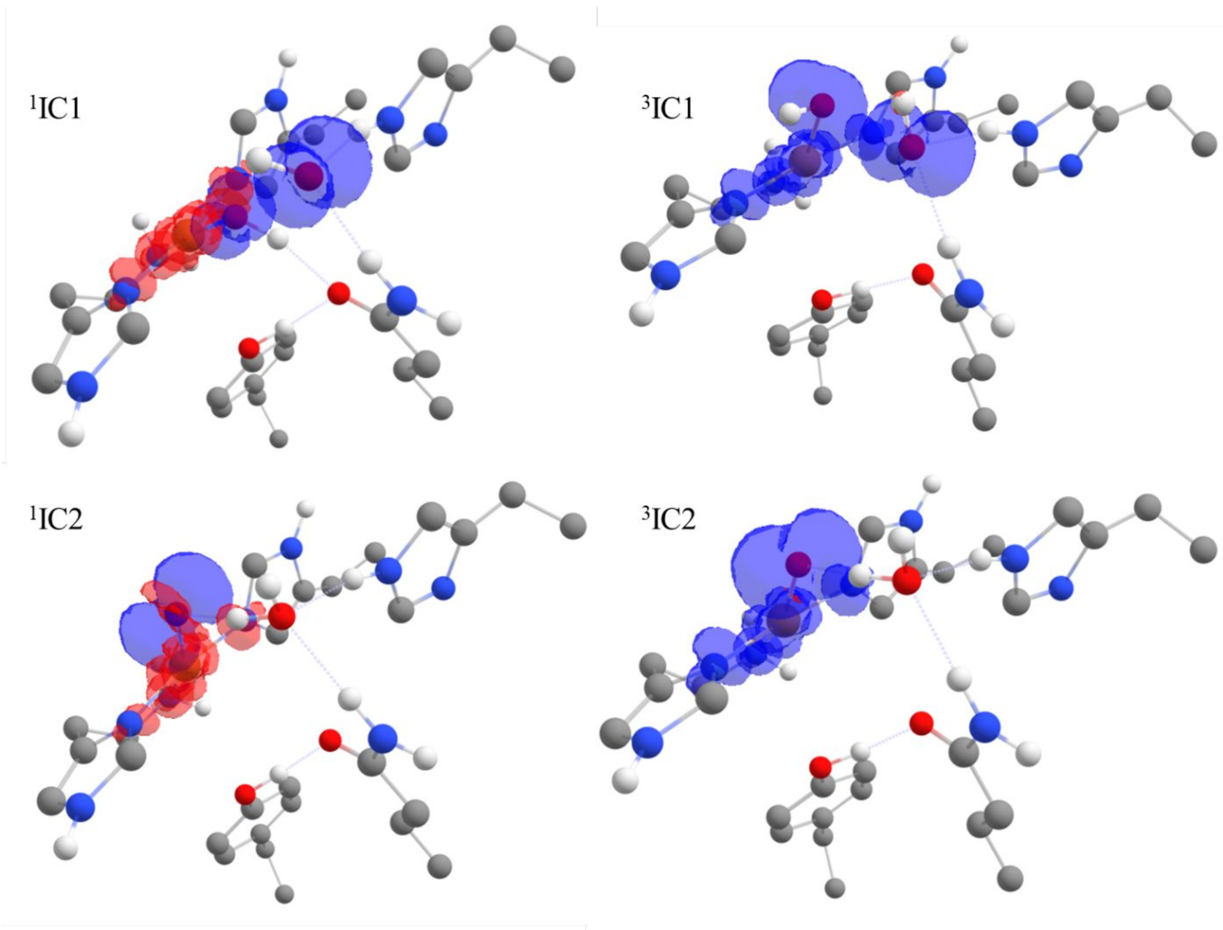

Figure S11. Spin density plots for intermediate complexes for the reaction with WT *Nc*AA9C. Net positive spin is indicated by blue and net negative spin by red.

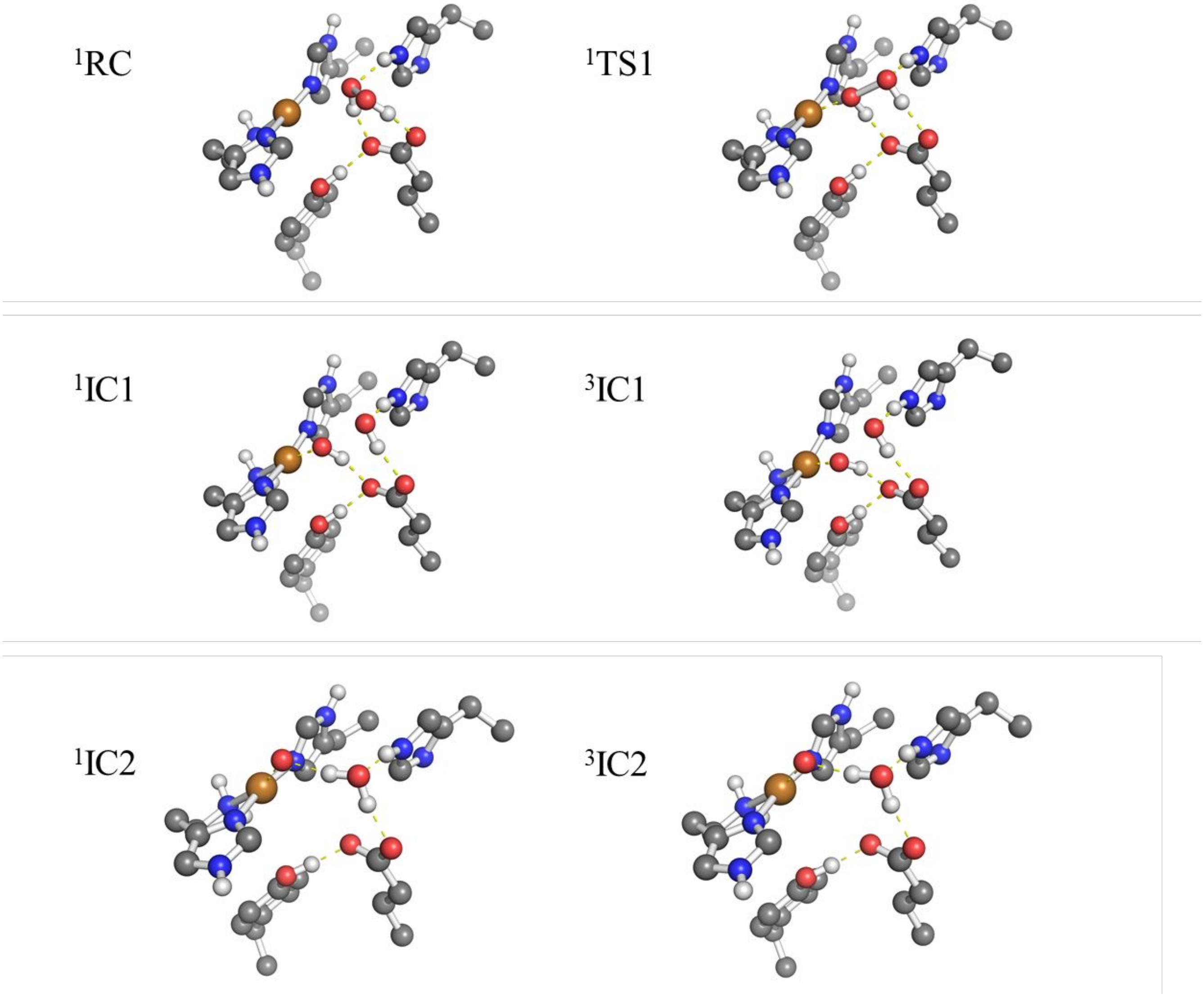

Figure S12. Optimized geometries by DFT for the Q164E in the glutamate form.
Optimized geometries are shown for the reactant complex (RC) Cu(I) + H_2O_2, the transition state of the O-O homolytic cleavage (TS), and the intermediate complexes of the Cu(II)OH + $OH^{\bullet}$ step (IC1) and the Cu(II)O• + H_2O step (IC2) found along the H_2O_2 splitting path of the reaction with the Q164E mutant in the glutamate form and in the Cu(I) oxidation state. The left superscript indicates the spin multiplicity.

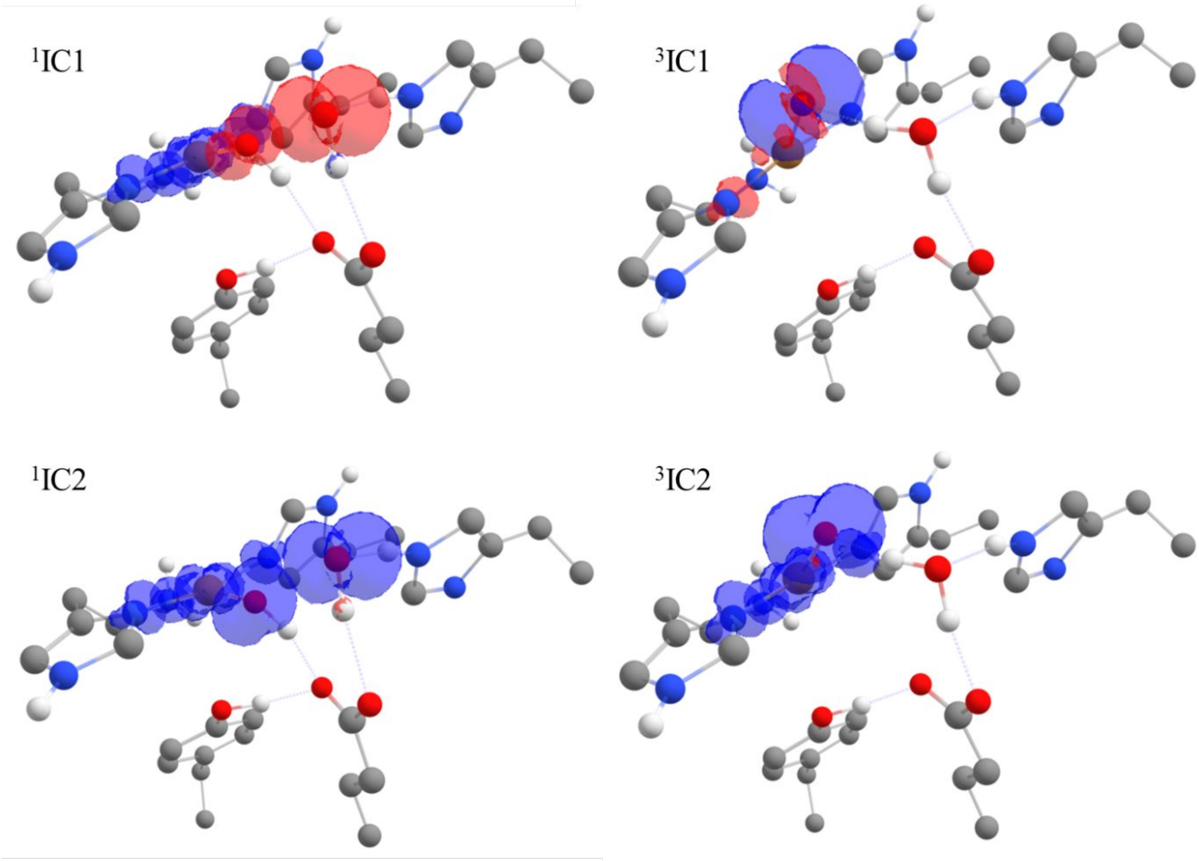

Figure S13. Spin density plots for intermediate complexes for the reaction with the Q164E mutant in the glutamate form. Net positive spin is indicated by blue and net negative spin by red.

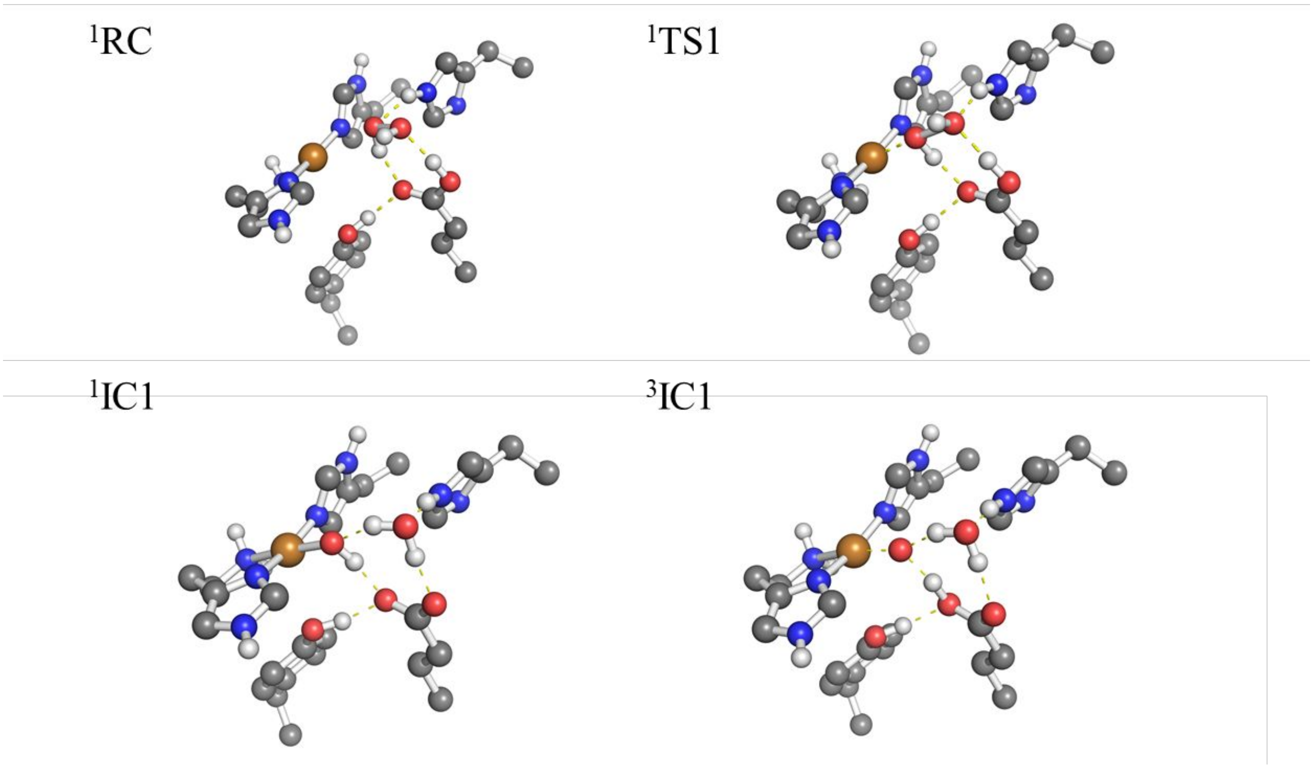

Figure S14. Optimized geometries by DFT for the Q164E mutant in the glutamic acid form. Optimized geometries are shown for the reactant complex (RC) Cu(I) + H_2O_2, the transition state of the O-O cleavage (TS), and the intermediate complexes of the Cu(II)O• + H_2O step (^{3}IC1) and the Cu(III)OH + H_2O step (^{1}IC1) found along the H_2O_2 splitting path of the reaction with the Q164E mutant in the glutamic acid form and in the Cu(I) oxidation state. The left superscript indicates the spin multiplicity. It is worth highlighting that in the case in which E164 is protonated, the Cu(II)OH + OH$^{\bullet}$ intermediate was not obtained for either spin multiplicity. The nature of the final reactive copper intermediate is either a Cu(II)O$^{\bullet}$ or Cu(III)OH depending on whether the spin state is set to be triplet or singlet, respectively. Attempts to converge the singlet open-shell spin-coupled Cu(II)O$^{\bullet}$ via broken-symmetry calculation starting from the optimized Cu(II)O$^{\bullet}$ geometry at the triplet state failed. As the optimization progresses, the proximal oxygen atom of the E164 carboxylate barrierlessly donates a proton to the copper-bound oxygen, followed by a large energy stabilization. The final stable minimum is a closed-shell singlet with a Cu-O distance consistent with that of known Cu(III)-OH coordination compounds (1.81 Å), indicating that after the protonation of the CuO core, the formal Cu(II)O$^{\bullet}$ evolves to Cu(III)-OH.

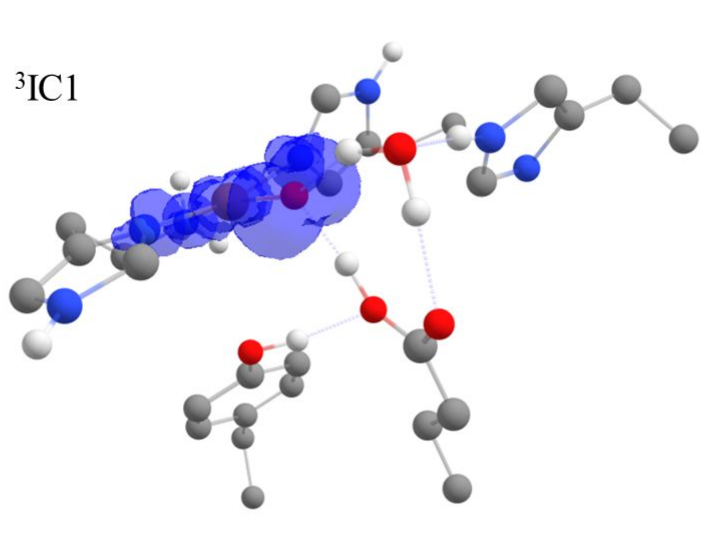

Figure S15. Spin density plots for intermediate complexes for the reaction with the Q164E mutant in the glutamic acid form. Net positive spin is indicated by blue and net negative spin by red.

3. Supplementary References

(1) Quinlan, R. J.; Sweeney, M. D.; Lo Leggio, L.; Otten, H.; Poulsen, J. C. N.; Johansen, K. S.; Krogh, K. B. R. M.; Jorgensen, C. I.; Tovborg, M.; Anthonsen, A.; Tryfona, T.; Walter, C. P.; Dupree, P.; Xu, F.; Davies, G. J.; Walton, P. H. Insights into the oxidative degradation of cellulose by a copper metalloenzyme that exploits biomass components. *Proc. Natl. Acad. Sci. USA* **2011**, *108*, 15079-15084.

(2) Borisova, A. S.; Isaksen, T.; Dimarogona, M.; Kognole, A. A.; Mathiesen, G.; Varnai, A.; Rohr, A. K.; Payne, C. M.; Sorlie, M.; Sandgren, M.; Eijsink, V. G. Structural and functional characterization of a lytic polysaccharide monooxygenase with broad substrate specificity. *J. Biol. Chem.* **2015**, *290*, 22955-22969.

(3) Peisach, J.; Blumberg, W. E. Structural implications derived from the analysis of electron paramagnetic resonance spectra of natural and artificial copper proteins. *Arch. Biochem. Biophys.* **1974**, *165*, 691-708.

(4) Stoll, S.; Schweiger, A. EasySpin, a comprehensive software package for spectral simulation and analysis in EPR. *J. Magn. Reson.* **2006**, *178*, 42-55.

(5) Kjaergaard, C. H.; Qayyum, M. F.; Wong, S. D.; Xu, F.; Hemsworth, G. R.; Walton, D. J.; Young, N. A.; Davies, G. J.; Walton, P. H.; Johansen, K. S.; Hodgson, K. O.; Hedman, B.; Solomon, E. I. Spectroscopic and computational insight into the activation of O_2 by the mononuclear Cu center in polysaccharide monooxygenases. *Proc. Natl. Acad. Sci. USA* **2014**, *111*, 8797-8802.

4. Sample Input for Geometry Optimization.

```
# wild type 1RC
!UKS B3LYP TightOPT TightSCF ZORA ZORA-def2-TZVP SARC/J D4 CPCM(Water) LargePrint UNO
UCO
%basis newGTO Cu "ZORA-def2-TZVPP" end
end
%pal nprocs 6
   end
%geom
  ConnectFragments
   {1 2 C 2 17 }
   {1 3 C 2 32 }
   {1 4 C 2 47 }
   {1 5 C 2 62 }
   {1 6 O 2 81 }
   {1 7 O}
   {2 7 O}
   {3 7 O}
   {4 7 O}
   {5 7 O}
   {6 7 O}
  end
        RelaxFrags {7} end
end

%output
Print[ P_Basis ] 2
Print[ P_MOs ] 1
Print[P_ReducedOrbPopMO_L] 1
Print[ P_Hirshfeld] 1
end

* xyz 1 1
N(1)  -4.12730549282390   24.03276433431390   33.10815546566136
C(1)  -4.55721656781710   24.36879849363456   31.74611228529338
C(1)  -6.07020306825674   24.62953155996522   31.68044264379724
C(1)  -6.88654530461255   23.37466701649166   31.71469248071470
C(1)  -7.68909482331828   22.81366488304959   30.74813592179624
N(1)  -6.91585196237749   22.55486041828731   32.83462365568323
C(1)  -7.71448558645411   21.53289693050616   32.55037814504380
N(1)  -8.19789726450330   21.65720206991578   31.29814117615868
H(1)  -6.36423708127049   25.29360412734643   32.51210203050663
H(1)  -6.29930071406571   25.16342712748984   30.74553208462342
H(1)  -7.94113952897872   23.13917329311267   29.74231114383963
H(1)  -7.96373112593124   20.71214828592462   33.22137527660701
H(1)  -8.84182478987122   21.01249907607665   30.84833442156977
H(1)  -3.18265228967468   23.64528255705214   33.09399036069838
H(1)  -4.07434327547339   24.87757695921803   33.68030235107351
H(1)  -4.29882074355611   23.52757530175217   31.08913698035585
H(1)  -4.02526267971877   25.26192797568477   31.36946551535118
C(2)  -1.22812877296685   22.38082527128091   39.16564942528873
C(2)  -1.43596009213949   22.97621391892951   37.77311091712991
C(2)  -2.82210544976662   22.86023456263504   37.23359228418670
C(2)  -3.23974975966914   22.58024794122033   35.95531303471175
N(2)  -3.98244428323136   23.03342724695175   37.95733229940824
C(2)  -5.03867044716877   22.86525041282130   37.12803328130614
N(2)  -4.61918919320251   22.59691094822118   35.89949392051646
H(2)  -1.14894965420389   24.04439731729838   37.79151233938443
H(2)  -0.76716172569160   22.49221180683584   37.04443706193684
```

H(2)	-4.04836658211731	23.23699539388126	38.95049885537130
H(2)	-2.62744867635622	22.37274147078306	35.08157315649595
H(2)	-6.07430054948458	22.94010546953378	37.45099121109836
H(2)	-1.51473498675430	21.31756534145198	39.18365671379318
H(2)	-1.82800322222321	22.91297247882478	39.92247215917264
H(2)	-0.17030763077594	22.47217076510176	39.45599617449945
C(3)	-2.76805360682632	17.47920716297753	41.25435040838092
C(3)	-3.41670882863877	18.87064714100959	41.20641867941882
C(3)	-4.02946888639000	19.18312600571196	39.87148896253171
C(3)	-5.34002971364920	19.45886549222016	39.53221181936365
N(3)	-3.24256475902338	19.23361054393790	38.73509163861129
C(3)	-4.05932735105222	19.53173263928074	37.74162783661969
N(3)	-5.33762614626211	19.67567332389130	38.17166135200189
H(3)	-4.19526914707594	18.94791171858907	41.98335826228524
H(3)	-2.65778736560153	19.63698982974497	41.44642920768591
H(3)	-6.24747393723431	19.51817999288598	40.12876672532667
H(3)	-3.78660079342214	19.66285961454565	36.69528634659375
H(3)	-6.12436315519005	19.93049833041857	37.56948708408677
H(3)	-1.97845200670988	17.38692832689354	40.49205000028694
H(3)	-3.51607655434903	16.69136575106984	41.06710825062957
H(3)	-2.31400972099137	17.29500623735546	42.24175675462229
C(4)	-1.90380816722361	16.37156786173235	32.92453757685686
C(4)	-2.79344154887780	17.47650008101297	33.49838655165643
C(4)	-4.09756828756396	16.91079575149474	34.04694148753819
C(4)	-5.08894827608984	17.90832400175537	34.60992880508213
N(4)	-6.12756483508710	17.38385604667018	35.26471622006016
O(4)	-4.96460509046227	19.14847693456127	34.46762069095583
H(4)	-2.25462681824471	18.02375020453619	34.29078844688504
H(4)	-3.00768368218469	18.22038210908972	32.71625342067070
H(4)	-4.63259369687092	16.36151173603642	33.25020608709323
H(4)	-3.89585313314916	16.16095095559237	34.83170541015288
H(4)	-6.22864243920749	16.37826288002508	35.34778155778713
H(4)	-6.92127384687819	17.97761582568670	35.51929581724369
H(4)	-2.41036988333238	15.83704096557766	32.10354416977032
H(4)	-1.63930628638870	15.62828181365993	33.69513995446516
H(4)	-0.96755053810219	16.79417377186441	32.52632984508829
C(5)	0.94935951461832	20.56850648277995	28.79561811003615
C(5)	0.64581743679240	21.77141473741393	29.70550817913036
C(5)	-0.65686145846700	21.55905838826100	30.45686953301892
C(5)	-1.88375035405406	21.66352749933449	29.77575228557258
C(5)	-0.70173980232601	21.15109632499410	31.79821895711467
C(5)	-3.09289434461985	21.34253711667277	30.38891797209503
C(5)	-1.91115679488277	20.83823250105406	32.43579333489289
C(5)	-3.11915198737811	20.90898026326117	31.72540266079145
O(5)	-4.32429298299982	20.58215468387946	32.24889699640556
H(5)	1.47031039239512	21.91716557313859	30.42283470763706
H(5)	0.58265231543487	22.68870938677091	29.09533215099497
H(5)	-1.89078441580368	21.98604631704807	28.72984198804543
H(5)	0.23049681662236	21.06376258144126	32.36496166013524
H(5)	-4.03521637177459	21.40419250626852	29.83911568820886
H(5)	-1.91824022921565	20.51941296187254	33.48102113469824
H(5)	-4.27943496465570	20.11791124486948	33.11486797694867
H(5)	0.14192286132475	20.42004696539256	28.06054916989736
H(5)	1.03876983632170	19.64460821637173	29.39020204990101
H(5)	1.89218062342124	20.71112587843319	28.24152993991518
Cu(6)	-5.55460013157716	22.69665716858110	34.20723877673480
O(7)	-7.96347535100680	19.59168065317512	35.40823574763269
O(7)	-6.89434077470710	20.49133247087957	35.80194711637260
H(7)	-8.59073273878322	19.71730277911601	36.14315575334990
H(7)	-6.14013526814182	20.11029649355781	35.26103496762313

5. Optimized Atomic Coordinates for WT *Nc*AA9C

Optimized atomic coordinates in Å – WT *Nc*AA9C [1]RC

WT 1RC -3459.533694939519

N	-4.12386266272451	24.04592176660164	33.10204441537460
C	-4.54602054561808	24.38882100980483	31.73823740636572
C	-6.05500648701803	24.65344818278102	31.66701404180446
C	-6.87025368727729	23.40781416432127	31.69233008093524
C	-7.66573070390606	22.84948127296997	30.73471360018484
N	-6.89821247180220	22.59075962374674	32.81705645060987
C	-7.69127865284750	21.57154791781155	32.53103514159067
N	-8.17218212170225	21.69397564107935	31.28348186057393
H	-6.34831947653550	25.30903570970178	32.49264876349373
H	-6.27661336780350	25.18734302606555	30.74098530691141
H	-7.91514802655023	23.16863372949937	29.73826988106657
H	-7.92931040083958	20.75319324111117	33.18791637737374
H	-8.80905227647917	21.05220256552462	30.83637980771147
H	-3.19297361507721	23.64323953403069	33.08904228127437
H	-4.06690506647821	24.88443778107406	33.67132147491198
H	-4.28907921979159	23.55694328628283	31.08479984221997
H	-4.01651318549242	25.27445692299927	31.37081748317334
C	-1.25266854927626	22.36091953736642	39.16451673725376
C	-1.45296220188046	22.96354354560574	37.77123375753698
C	-2.83445174514474	22.85739185108844	37.21836496350529
C	-3.25336790867539	22.57331087131309	35.95020746718185
N	-3.99052094591965	23.08900760377990	37.92832050804171
C	-5.04456343422423	22.94808516209668	37.09948204314576
N	-4.63249562657672	22.64194182743114	35.88236110958267
H	-1.16518103075419	24.02049681586499	37.79799002847538
H	-0.78583070369874	22.48127881106157	37.05452961391282
H	-4.05473909183094	23.31459069185411	38.90925381456945
H	-2.65393635179893	22.33099042758130	35.09054818739408
H	-6.06690201071145	23.06807238876783	37.41274114668223
H	-1.53945157583813	21.30864962817887	39.17676826735462
H	-1.84882774368188	22.88663471795365	39.91372544871331
H	-0.20561247820963	22.44737545617498	39.45724918388014
C	-2.81529238125377	17.45505818300271	41.22622288032221
C	-3.45844688924828	18.84524653941480	41.18119495647773
C	-4.06346824282954	19.16776017113818	39.85122397231557
C	-5.33526875751709	19.56788900585203	39.53396057197376
N	-3.29590432552716	19.13423769146548	38.69998206241357
C	-4.09410989242787	19.50705615172293	37.72204889226021
N	-5.33702530776094	19.77841459557480	38.17410730673394
H	-4.23335971119643	18.92094708617048	41.94804415991801
H	-2.70293742297738	19.59932500172549	41.42591758215939
H	-6.21307692517034	19.71998485943370	40.13829420977249
H	-3.82716022019382	19.60243097938617	36.68269630528680
H	-6.10553692305748	20.09510729978243	37.59578897873708
H	-2.03014580415334	17.36460347539689	40.47437431394504
H	-3.55790678299132	16.67706865247115	41.03414831705300
H	-2.37031792436215	17.26732706636298	42.20578079182681
C	-1.91674756621070	16.37907433943807	32.89588914755763
C	-2.80402727250668	17.48172657503524	33.46904815985973
C	-4.10335389522390	16.89576685781070	34.00228841190857
C	-5.08312743178184	17.85794501908844	34.62988263476956
N	-6.00953861232511	17.29933996110590	35.41055008370650
O	-5.05982475818045	19.08960127658190	34.42604635079297
H	-2.27171564828087	18.01884649947178	34.25862275174470

451	H	-3.00847674324597	18.22011205541768	32.69372082301528
452	H	-4.64398870460190	16.40541596403938	33.18396129298067
453	H	-3.88947194197706	16.10732049464956	34.72906735894041
454	H	-6.02831971735579	16.30226407203289	35.54860370855967
455	H	-6.78983758369258	17.84999214701453	35.75184756688442
456	H	-2.41653428569354	15.85407088059614	32.07789684385682
457	H	-1.66006475861746	15.63866507605956	33.65768086617901
458	H	-0.98591104893906	16.79698624660992	32.50709011657807
459	C	0.96631984385224	20.58499381824104	28.79698761402520
460	C	0.66250240294157	21.78295559981061	29.70887097935577
461	C	-0.64102314805282	21.56395503390241	30.44723733601774
462	C	-1.85471505904973	21.66218166086267	29.75882384181254
463	C	-0.69389256099515	21.15931605200872	31.77982386797424
464	C	-3.06059226682954	21.33244276004857	30.35602472548936
465	C	-1.90062626671911	20.83718343678435	32.40196988464938
466	C	-3.09153698636575	20.89772404517925	31.68175967294701
467	O	-4.30623505415082	20.56098864993875	32.19305510068774
468	H	1.47712432908394	21.92147773634790	30.42349838205907
469	H	0.60527406031087	22.69398973311516	29.10701326807975
470	H	-1.85389108024696	21.98398637879741	28.72317508443934
471	H	0.22513147849078	21.07954490021353	32.35002169212234
472	H	-3.98897258492883	21.39478111156053	29.80179056740400
473	H	-1.91390136077611	20.52136501632030	33.43773487131472
474	H	-4.26242257610379	20.10295891637903	33.05599905275897
475	H	0.16924340425187	20.44308106876353	28.06456941596447
476	H	1.04979085362895	19.66674747422338	29.38287160273211
477	H	1.90347414485522	20.72600459002190	28.25266007198539
478	Cu	-5.59796853989947	22.72587861280559	34.22618142525107
479	O	-8.04437407165821	19.39809204558978	35.76040164185675
480	O	-7.04399678404845	20.46448860136054	35.74707096903894
481	H	-8.38334071779341	19.47676871637277	36.66632263667562
482	H	-6.31088061432975	20.02568810596003	35.23477938399530

Optimized atomic coordinates in Å – WT *Nc*AA9C ^{1}TS1

WT 1TS1 -3459.526645317755

N	-4.20589050435313	24.00749382138492	33.13175584326596
C	-4.59113014467865	24.34182366822432	31.75133943890823
C	-6.09550338503379	24.59441638935503	31.68522492885766
C	-6.90112364602394	23.34396410238406	31.71511996947278
C	-7.69153721499201	22.79345794900706	30.74886588786955
N	-6.93334006383790	22.51663970989746	32.82741454473783
C	-7.72486812391928	21.49774984747674	32.53101377155489
N	-8.20086994500288	21.63406366727308	31.28567686294384
H	-6.39066024016453	25.24909122748960	32.51091657255781
H	-6.32412114779766	25.12539657469385	30.75917558799147
H	-7.94013610278588	23.12556139301986	29.75649635221242
H	-7.95501071462813	20.67487276389483	33.18321027645600
H	-8.83602728945144	20.99583344288531	30.83075649552944
H	-3.26804761221714	23.62125925042938	33.15259686422684
H	-4.18592123154285	24.84818013108565	33.70052083759332
H	-4.32145795767314	23.51214473101264	31.10099531099803
H	-4.05780649334235	25.22418776908559	31.38316622435443
C	-1.25088796220072	22.34725747423984	39.16925229957275
C	-1.45993303975133	22.94529972270117	37.77839287828517
C	-2.83048860031185	22.83974542830205	37.21332685156661
C	-3.21694798896648	22.62192803527450	35.92285371872221
N	-4.00174470286818	22.99169985530219	37.91703157860402
C	-5.03740618129836	22.86100380389699	37.06684677900773
N	-4.59169990220101	22.64557632788148	35.84325553139139
H	-1.17865489048744	24.00436150996859	37.80640610906676
H	-0.79076948227981	22.46520805533293	37.06182658421893
H	-4.08681093626610	23.15321563032255	38.90906429391087
H	-2.59580004802060	22.44277172530802	35.06337704414384
H	-6.06872406897299	22.91105976692765	37.36665092269150
H	-1.53010898117965	21.29285964485944	39.18400735534158
H	-1.84838397529176	22.86994125050741	39.92001194494408
H	-0.20357902898354	22.44162733955120	39.45818716704085
C	-2.77174343692192	17.43360460438573	41.24380381707821
C	-3.42565426296300	18.82052959476391	41.19890481657526
C	-4.04112676463126	19.14859329291048	39.87582997587583
C	-5.35685518985350	19.39840317072717	39.58080705288822
N	-3.29141674310358	19.23523249307294	38.71874124811423
C	-4.14504619077965	19.53123807216910	37.75778287188994
N	-5.40592590356669	19.64140672968523	38.22915626933256
H	-4.19782199387671	18.88961351239986	41.96878607130456
H	-2.67151035954098	19.57713175179360	41.44044673392712
H	-6.23786624873042	19.42270853237228	40.19934173134426
H	-3.90475472702741	19.68018803215743	36.71831495465777
H	-6.23869439046992	19.82960835206874	37.67471324418559
H	-1.98817651423430	17.34760553882072	40.48905068856143
H	-3.50943832470199	16.65002762807236	41.05541032321147
H	-2.32210488176898	17.25052150569286	42.22208138437333
C	-1.89397806610846	16.35163728256935	32.91174940124939
C	-2.78672488807773	17.44823812638111	33.48609173027591
C	-4.09532777544313	16.90243784044328	34.03850263150113
C	-5.02792829041922	17.94648049953149	34.61190895176350
N	-6.08763918169126	17.49298231234130	35.27834820270481
O	-4.83064774224083	19.17155179980131	34.47210722249208
H	-2.25196053749558	17.98746529470888	34.27223728397959
H	-2.99611765423581	18.18136763583067	32.70650125101611
H	-4.64749996408717	16.37977362112181	33.24940611763092
H	-3.90591455301163	16.15509126802695	34.81477954870316

543	H	-6.23757078533635	16.50459138355137	35.39770203429830
544	H	-6.76847766653726	18.15189146163871	35.65183025244006
545	H	-2.39288599170959	15.82178885013087	32.09609922991265
546	H	-1.62933543077252	15.61403435836400	33.67388472230539
547	H	-0.96766506492926	16.77554460942297	32.51922030775932
548	C	0.94482893512103	20.57206122264113	28.79683825434189
549	C	0.63574268237796	21.76855441359194	29.70759319730571
550	C	-0.66111482848543	21.54582059064379	30.45170042808189
551	C	-1.87510959251576	21.64663845488728	29.76455718564323
552	C	-0.70981544310998	21.14446959976178	31.78478363369792
553	C	-3.08066601234344	21.32374541461888	30.36467219393088
554	C	-1.91627585061096	20.83024746516737	32.41004811498917
555	C	-3.10873840507018	20.89532841652093	31.69208273063989
556	O	-4.32023709904972	20.57028051953765	32.21458981493587
557	H	1.45257822025995	21.91290971299090	30.41878490640056
558	H	0.56997273286860	22.67817360095057	29.10444499923187
559	H	-1.87370090497395	21.96620700507352	28.72820810391456
560	H	0.21094768000666	21.06352977855818	32.35201570366443
561	H	-4.01047202384437	21.38738278304794	29.81294060210330
562	H	-1.92989790310488	20.51810380284562	33.44685152169879
563	H	-4.25843631441430	20.09392445180533	33.07051926979143
564	H	0.14623513326638	20.42338123445039	28.06734128142344
565	H	1.03687967119004	19.65527382907243	29.38375660871893
566	H	1.87908335797264	20.71897326332019	28.24907536681634
567	Cu	-5.64111868659857	22.60853403145726	34.24370695506846
568	O	-7.54185933975125	19.80078523055948	36.26240930423040
569	O	-6.75733659008690	20.97056291989037	35.34407902192108
570	H	-8.44340621895751	20.11041276619196	36.09803361056589
571	H	-6.05326206934151	20.34344132840649	35.04270129136063

Optimized atomic coordinates in Å – WT *Nc*AA9C ^{1}IC1

WT 1IC1 -3459.544394560962

N	-4.30062600304955	23.98581071565276	33.15576388300666
C	-4.62034843523909	24.31406665448948	31.74336868023329
C	-6.11407102015978	24.56037174546563	31.67713275933198
C	-6.91252697214773	23.31312651846735	31.70743780416007
C	-7.70289048999554	22.75379757215439	30.74579527995018
N	-6.93736953351116	22.49960695363675	32.81307594622327
C	-7.72202123749733	21.46803297847387	32.53717333822789
N	-8.20560057466174	21.59737495656776	31.29676381360941
H	-6.41136720047376	25.21466803127508	32.50226612863296
H	-6.34397916952426	25.09018202322832	30.75052973792774
H	-7.96094181228008	23.07959264691246	29.75385427904391
H	-7.92797521958553	20.65076625806942	33.20282324151819
H	-8.84149732031305	20.95309732165737	30.85067475043002
H	-3.35752559397869	23.61692001421074	33.23158109096101
H	-4.32714409117301	24.83247609999950	33.71695684323439
H	-4.33406388493731	23.48724729591834	31.09829865541959
H	-4.06592344915720	25.18279161113478	31.38031372667986
C	-1.26527658300698	22.33204193224801	39.16408314531147
C	-1.47474339131217	22.92609058511001	37.77861604680090
C	-2.82687957615118	22.83042485653715	37.19339167268069
C	-3.16887952768603	22.67094321996897	35.88511731045433
N	-4.01362575126093	22.98562627798907	37.86736237533159
C	-5.02323989604300	22.90938860539055	36.98513838966389
N	-4.53444249847792	22.73236806083861	35.77152058269307
H	-1.19593486242728	23.98621094985983	37.80818847299096
H	-0.80227626249269	22.44508030388395	37.06512564515717
H	-4.12482087509922	23.11537172599360	38.86180031810903
H	-2.52258391106685	22.50866272191618	35.04107664698837
H	-6.06385673671563	22.96275453678185	37.24835536851714
H	-1.54147405669525	21.27670583849305	39.17942268512635
H	-1.86468491989941	22.85360391664235	39.91452930089417
H	-0.21842796660162	22.42962929345601	39.45325691823995
C	-2.77248455059442	17.41530223497582	41.24127503714826
C	-3.43061611631645	18.80072832982797	41.19528031451926
C	-4.04742377102512	19.12883877565262	39.87307104932690
C	-5.36572030301653	19.35795855203833	39.56981211238583
N	-3.29129670476995	19.24267047313112	38.72311930292700
C	-4.14598646231419	19.53283345626220	37.75958559589614
N	-5.41170568474599	19.61584931763798	38.22087678502843
H	-4.20291408663164	18.86621071656214	41.96553446183357
H	-2.67840997801193	19.55929739306937	41.43662623704908
H	-6.25065517502083	19.35830444259198	40.18359432902993
H	-3.90295395283971	19.69147183837713	36.72222125074360
H	-6.25273692328389	19.77607422874906	37.65102140361599
H	-1.98836541823255	17.33109532191828	40.48675001863788
H	-3.50784301043370	16.62946316637959	41.05314986476289
H	-2.32258816564934	17.23413318746140	42.21987590089120
C	-1.88899039222494	16.33061697368422	32.91018374300508
C	-2.78397916209483	17.42361465250302	33.48283838164757
C	-4.09659038373577	16.89413249152773	34.04040375849725
C	-4.99661956598068	17.97460165147294	34.60212666123728
N	-6.13751338027629	17.57919054693725	35.16312194422601
O	-4.68659761307118	19.18165555296611	34.55128505349317
H	-2.24967914018621	17.96359848975226	34.26827903928844
H	-2.99465233903633	18.15545520897982	32.70125261976855
H	-4.65692024081687	16.36241303043171	33.26414260749957
H	-3.91407586787597	16.16180803667041	34.83338913950936

632	H	-6.36702072590380	16.60012060583467	35.21504988109012
633	H	-6.78261427950346	18.27129596331310	35.55659603357500
634	H	-2.38616557050567	15.79873708056213	32.09461287207743
635	H	-1.62240037696707	15.59415364485409	33.67298827744174
636	H	-0.96376631244970	16.75697741615805	32.51766146015584
637	C	0.93881789155349	20.55666498440409	28.79347397977800
638	C	0.62605675124911	21.75251098409863	29.70313031794217
639	C	-0.66892193363237	21.54022404154045	30.45401940092068
640	C	-1.89805802648971	21.64244035816009	29.79697698308200
641	C	-0.68623205204082	21.15063908441185	31.79271621521082
642	C	-3.09088055095460	21.34544367920853	30.43898511430253
643	C	-1.87622048839541	20.86276463213586	32.45745499356173
644	C	-3.08759736749799	20.94561689337149	31.77518578173065
645	O	-4.28964789764269	20.67357513658153	32.35508274204165
646	H	1.44193742089735	21.89980387850675	30.41437220126897
647	H	0.55842323002426	22.66009818879306	29.09666255536876
648	H	-1.92292496107713	21.94535233790704	28.75597423760028
649	H	0.24995715264030	21.06406029479380	32.33309108625560
650	H	-4.03503322784845	21.41174365685928	29.91250308139294
651	H	-1.86513098791148	20.56348029315137	33.49809153825898
652	H	-4.20540921616167	20.13549613886481	33.17482356255969
653	H	0.14077817823910	20.40518217041081	28.06372583276002
654	H	1.03335738905422	19.64044874580902	29.38104851843958
655	H	1.87266770349638	20.70592146991454	28.24599113302931
656	Cu	-5.61285938907578	22.58684667758060	34.19863784509418
657	O	-7.55704873957633	19.69844512529546	36.41638299623977
658	O	-6.60217003118782	21.18248877762350	35.13128868310064
659	H	-8.33421120397436	20.25172339838481	36.26693956400930
660	H	-5.95172516753826	20.45531704941219	35.08693266206278

Optimized atomic coordinates in Å – WT *Nc*AA9C ^{3}IC1

WT 3IC1 -3459.539979206429

N	-4.32324568065441	23.90496990723359	33.16120827183146
C	-4.64325907680544	24.23258935092651	31.74362005476310
C	-6.14132417055788	24.45157624877323	31.67130709810455
C	-6.91770058987167	23.18822227521275	31.69433693225748
C	-7.69599981625282	22.60903075377162	30.73312865196197
N	-6.92715885361326	22.37746022219009	32.80043568597438
C	-7.67491472276813	21.32118042547654	32.52134355308844
N	-8.15831787503213	21.43433932355306	31.27780843489068
H	-6.45428745376309	25.09767562676976	32.49687264820519
H	-6.37685161826266	24.97997254555314	30.74503905049322
H	-7.95555329052425	22.92291330229555	29.73788296568859
H	-7.87058012280600	20.48850431404684	33.17479709464651
H	-8.76724940849877	20.76761399117574	30.82692004531057
H	-3.40759675307343	23.47200826104952	33.22897398172282
H	-4.27879048943841	24.76101383267110	33.70683449250787
H	-4.33941310383002	23.41310142559817	31.09729437743995
H	-4.10334093109048	25.11217170302529	31.38577328430816
C	-1.28593101273223	22.29032355062505	39.17362891258479
C	-1.50026955755288	22.88509843281688	37.78782762934097
C	-2.85841668922995	22.75105638063098	37.22163195677755
C	-3.25715797010730	22.58522477215454	35.93010343123127
N	-4.01633090943193	22.93655785927330	37.93985939262895
C	-5.05957175943532	22.88306984041948	37.10027191877694
N	-4.62781773344636	22.67855772953592	35.86811807868339
H	-1.24299822086076	23.95020755230406	37.81461804824076
H	-0.81611664906931	22.41922991734296	37.07511886384921
H	-4.08496120184530	23.08017825059749	38.93598598298307
H	-2.64841376727612	22.40531881866606	35.06201368444959
H	-6.08823879714178	22.97337298747903	37.39464003235989
H	-1.54264596809327	21.23019146668444	39.18460329660801
H	-1.89805148434518	22.79833486487249	39.92266816778314
H	-0.24231923827584	22.40631676934347	39.46785891100546
C	-2.71146416008957	17.34033431522548	41.22941012450067
C	-3.39456816191295	18.71301520047905	41.18452919867303
C	-4.00989023199573	19.02748584595706	39.85610304001639
C	-5.29905450731130	19.37129565176699	39.53568265274565
N	-3.23648512582315	19.05250145756244	38.71005256586236
C	-4.05100384124557	19.40333074407728	37.73410403119016
N	-5.30780741161711	19.60760353820817	38.18016816339006
H	-4.17185307742463	18.76318724608045	41.95124232628922
H	-2.65997378874083	19.48687286820421	41.43114327119497
H	-6.18511917083805	19.46751358622806	40.14006355980776
H	-3.78647224221306	19.52487856694581	36.69683104870256
H	-6.10160263347390	19.87868278102037	37.58559163393619
H	-1.92289524017657	17.27291179135256	40.47853929912870
H	-3.43133364949063	16.54167052809995	41.03563508800099
H	-2.26270152065999	17.16452221992306	42.20949709153593
C	-1.77075472208304	16.29738908176501	32.89928866428082
C	-2.68983802166578	17.37375380136513	33.47204344363190
C	-4.01514425141907	16.88425797081147	34.03998942003667
C	-4.87433181272026	18.03352460023615	34.54561721639284
N	-6.05575560315616	17.72262563208488	35.08524137075003
O	-4.50201994975106	19.21641024006200	34.47669541232758
H	-2.16200360355801	17.91603369440186	34.26019152929366
H	-2.90595242580124	18.09972167127122	32.68681026977625
H	-4.58925637567548	16.33628736170628	33.28577939637793
H	-3.85931858742759	16.18714039596501	34.86970761249598

721	H	-6.35821604133019	16.76627874782734	35.17037608261594
722	H	-6.64949366835499	18.47181636224496	35.43356801562182
723	H	-2.25443981993462	15.75883230232237	32.07978602657223
724	H	-1.49399885608876	15.56351149082903	33.66127414242111
725	H	-0.85211509793831	16.74176286755896	32.51236511500559
726	C	0.99708614579807	20.58729669468253	28.80794550049466
727	C	0.65837471113381	21.77394713477801	29.71933127359749
728	C	-0.63462089778353	21.52988155554772	30.45962279863166
729	C	-1.84564495262826	21.61852625235302	29.76552181648301
730	C	-0.68519961694811	21.13809424044312	31.79474668738633
731	C	-3.05284032092530	21.30289563157500	30.36459604256978
732	C	-1.89347639564509	20.83107095039253	32.41901739759484
733	C	-3.08562199420998	20.89640640241907	31.69896689821293
734	O	-4.29712537890000	20.59873315704331	32.23172438889521
735	H	1.46928996221890	21.93299837006553	30.43439728944291
736	H	0.57615147934660	22.68328476781328	29.11777500714533
737	H	-1.83973222937081	21.92771780905062	28.72605561164113
738	H	0.23412853206900	21.06399837968519	32.36527783522577
739	H	-3.98125239595867	21.35973610805399	29.80964297122111
740	H	-1.90979575610474	20.52826885436385	33.45821126105760
741	H	-4.22132361627974	20.12217182780543	33.09201824284483
742	H	0.20529737786747	20.42334939501889	28.07417017202517
743	H	1.10586504774646	19.67125126132046	29.39321129606730
744	H	1.93057142215587	20.75541555749617	28.26509602832774
745	Cu	-5.78751951277918	22.68666901744364	34.32597143684406
746	O	-7.31526282918268	20.17167780804020	36.23561195705270
747	O	-7.28896418364426	22.28713660850198	35.54499587431926
748	H	-8.14020539126160	20.46260137500158	36.65079982549825
749	H	-8.07687069109537	22.08405857737007	35.02545164420498

Optimized atomic coordinates in Å – WT *Nc*AA9C ^{1}IC2

WT 1IC2 -3459.554822565371

N	-4.27583877889292	23.95172558144270	33.13699783840516
C	-4.60260090415267	24.28115340380222	31.72288314071669
C	-6.09906531284887	24.51687365033513	31.64994843989962
C	-6.88943372445389	23.26241601597544	31.67509897063860
C	-7.67635850009545	22.68174826134435	30.72197389809590
N	-6.90331931114362	22.45928055788248	32.78532040725010
C	-7.66930766330390	21.41229456964395	32.52582891626566
N	-8.15380743647384	21.51950459830211	31.28200783476987
H	-6.40488730660856	25.16788477789252	32.47434403361926
H	-6.32854890419935	25.04616567782449	30.72284452823076
H	-7.93288986304521	22.98661393600154	29.72314724399835
H	-7.87578234192245	20.59373793072377	33.19226465766921
H	-8.77321896776931	20.85680711878210	30.83948612262678
H	-3.36802234599167	23.50062406465002	33.19365140762419
H	-4.20358273596999	24.80925315928356	33.67782048099909
H	-4.30943415869561	23.45689590691955	31.07750258795217
H	-4.05403716217111	25.15437887170916	31.36343239329162
C	-1.26891857675789	22.31475025228117	39.15668027321563
C	-1.47612415525328	22.90849568673443	37.77201245077036
C	-2.83409878215868	22.79011564401387	37.20365041697129
C	-3.21622877303615	22.65419152446299	35.90386080956339
N	-4.00036231379861	22.94884011163307	37.91393252676836
C	-5.03313244785933	22.91298502592535	37.05821622122253
N	-4.58403546224426	22.74414009751209	35.82805502403239
H	-1.20704028158852	23.97081274121643	37.79662634120234
H	-0.79762361415173	22.43451613394899	37.05924348194175
H	-4.08120041132658	23.06701749498338	38.91265050118269
H	-2.59581094337436	22.49717602239226	35.03953759686062
H	-6.06527588696718	23.00756310115485	37.34686019355505
H	-1.53747031484233	21.25751241996611	39.16948659754611
H	-1.87547022322706	22.83093262775126	39.90482052694439
H	-0.22406782221722	22.41960588732472	39.45085106572047
C	-2.74984144830860	17.38461665063192	41.22098001837966
C	-3.41741863736191	18.76443966698768	41.17360957087754
C	-4.02920426834835	19.08643298360176	39.84819951788529
C	-5.34277345833889	19.33097683062775	39.53946018909092
N	-3.26647245883734	19.18238029201962	38.70021472604991
C	-4.11159716933937	19.47771249909584	37.73116641112366
N	-5.37782394306122	19.57865951916931	38.18817809071113
H	-4.19392753412369	18.82428963154554	41.94006208632921
H	-2.67261551233478	19.52890483949845	41.41922416738247
H	-6.23032905258460	19.34850626865235	40.14887989494843
H	-3.86441589137232	19.62952972485500	36.69337420489803
H	-6.20336455265997	19.76804754775285	37.61639622994524
H	-1.96172125874252	17.30704588172463	40.47006944575476
H	-3.47857115891315	16.59368996555043	41.02851636538796
H	-2.30317435812759	17.20553052812147	42.20143298758016
C	-1.81993371014904	16.31657615094254	32.89282363280724
C	-2.72678689854074	17.40388004600116	33.46343011128310
C	-4.04798326636881	16.89877065430633	34.02346100658291
C	-4.92404608997250	18.01238859714272	34.57135782959229
N	-6.04000454805973	17.63934984469677	35.20273331250900
O	-4.63150544787118	19.21434213051796	34.45409237185427
H	-2.19577169390995	17.94391914771873	34.25146576086021
H	-2.93711492458440	18.13306763184435	32.67968431511008
H	-4.62072386842466	16.37584779207620	33.24992084339899
H	-3.88406625331880	16.16922228185794	34.82266976559277

H	-6.27157165440067	16.66659100720102	35.31815173250226
H	-6.66458789903663	18.35357919956888	35.57182422966563
H	-2.30948302745389	15.78204432069526	32.07425821935971
H	-1.55150187502090	15.58108429349744	33.65604284372811
H	-0.89619474087401	16.75005742360596	32.50517123818913
C	0.99606285060035	20.56810191661988	28.79429512494661
C	0.67044125763918	21.76036236387142	29.70377819207346
C	-0.62622046049791	21.53157721907045	30.44469651441646
C	-1.83947053867861	21.62903513923258	29.75604317311209
C	-0.67541388064987	21.14009827206236	31.78056627468978
C	-3.04636229134038	21.32092239235696	30.36089801926523
C	-1.88288350232969	20.84090948016312	32.41039600578114
C	-3.07747746036210	20.91272624730486	31.69514671173282
O	-4.28965943977757	20.62124932197118	32.23073963876933
H	1.48269716389686	21.91195092792193	30.41877795023478
H	0.59851959456856	22.66931521886313	29.10026333939048
H	-1.83669450168699	21.93771996928049	28.71639485190522
H	0.24571842035692	21.05935748523260	32.34728681482963
H	-3.97632558275866	21.38368274351352	29.80914877085728
H	-1.89723533196213	20.53870353970787	33.44999623311192
H	-4.22585658556126	20.12617490759006	33.08148089333912
H	0.20257560356205	20.41171466543923	28.06071189942639
H	1.09454449388965	19.65192571792423	29.38118807048113
H	1.93142166430052	20.72483154824161	28.25127179198591
Cu	-5.73973444769975	22.80260395901529	34.27740329225449
O	-7.53790240113735	19.94085235819927	36.29708976343215
O	-7.26416634589618	22.37334334177874	35.32104348344765
H	-8.44469239097421	19.86504496621861	36.61736570367437
H	-7.46874386050891	20.84002309304007	35.85614043781190

Optimized atomic coordinates in Å – WT *Nc*AA9C ^{3}IC2

WT 3IC2 -3459.559148125441

N	-4.26256699476813	23.96673392417873	33.12881829742215
C	-4.58935726994553	24.29602108180920	31.71563570655340
C	-6.08566456235595	24.53624099334028	31.64231270516099
C	-6.87985013284528	23.28404798533820	31.66830017227467
C	-7.66755419725076	22.70929638553826	30.71112090606002
N	-6.89814484009107	22.48080976505064	32.77633645020551
C	-7.66909807820565	21.43882035660247	32.51334572650173
N	-8.15292837826343	21.54925598716743	31.26872076070854
H	-6.38964399274462	25.18877644726245	32.46618852300512
H	-6.31347733396376	25.06557763004884	30.71469577608861
H	-7.92389597902211	23.01852915702654	29.71352090362478
H	-7.87950723000141	20.61955977008499	33.17807548989158
H	-8.77569931438448	20.89084442072273	30.82473191761105
H	-3.35054187040912	23.52430882099022	33.18629404887855
H	-4.19952784010924	24.82386887866550	33.67101342239514
H	-4.29928029493408	23.47023577718133	31.07062371387951
H	-4.03964569121056	25.16847691178778	31.35516854765897
C	-1.26302650897697	22.32529947823053	39.15127984264819
C	-1.46824547151922	22.91849951491688	37.76645153274681
C	-2.82722345691509	22.80092144819973	37.20358466240406
C	-3.22202904618098	22.64420918867213	35.91010289213003
N	-3.98235003645772	23.01455839660943	37.91744555710994
C	-5.02183219707691	22.99398701480205	37.07025915970063
N	-4.58627536780410	22.77971786743031	35.84099947940928
H	-1.19658628526822	23.98019521446621	37.78783756751798
H	-0.79106739969175	22.44198497053156	37.05403216801390
H	-4.05015918039204	23.16728027737546	38.91244308716686
H	-2.61398961969853	22.45012451405033	35.04460366802878
H	-6.04739800556908	23.13400249653198	37.36243408073125
H	-1.53471412330947	21.26895217496579	39.16483769951024
H	-1.86814943148073	22.84386532100608	39.89898925278979
H	-0.21783479398297	22.42726256242609	39.44552179938974
C	-2.75889954798284	17.40114089300995	41.21905626215419
C	-3.42233295533684	18.78287590802948	41.17057891152768
C	-4.03190682440361	19.10318678687592	39.84458512980921
C	-5.35356878537007	19.29325662212143	39.53292864063395
N	-3.27052397501318	19.23425114900688	38.69972236487327
C	-4.12470362009248	19.49694591294829	37.72874691960325
N	-5.39524700958295	19.54433978616184	38.18299836535400
H	-4.19865438219210	18.84537007804479	41.93691712960121
H	-2.67529149483877	19.54523399540926	41.41571009656459
H	-6.24282217181826	19.27025903938853	40.13971575569124
H	-3.88134041376795	19.66079162229848	36.69188608863765
H	-6.23093785829360	19.68665544411834	37.61293376010634
H	-1.97083680524732	17.32065207005034	40.46821898114219
H	-3.48995661114703	16.61225528051279	41.02709171242349
H	-2.31286547363533	17.22144281151849	42.19974690551126
C	-1.83117366706261	16.32413784898291	32.89181409914954
C	-2.73440526936706	17.41403320331003	33.46122051752239
C	-4.05160597422656	16.89505441581535	34.01645151360501
C	-4.94557074769450	17.98850695757304	34.57244836474566
N	-6.04433246930541	17.58437235615156	35.21531093078519
O	-4.68430304269096	19.19700681582510	34.44979615645820
H	-2.20401134876840	17.95469106127605	34.24935707836710
H	-2.94387360454324	18.14456056777814	32.67857543119843
H	-4.61659960397522	16.37258633502276	33.23669555548731
H	-3.88024857175526	16.15891225819790	34.80792159846075

899	H	-6.24759840997171	16.60481749692272	35.32662755339621
900	H	-6.69221689804274	18.27601856174459	35.58707747269029
901	H	-2.32219562444214	15.79048286470118	32.07361974062812
902	H	-1.56504438006571	15.58845376153270	33.65557398316293
903	H	-0.90599602920790	16.75461986990283	32.50391138241069
904	C	0.99799937643962	20.56419656466870	28.79047715635131
905	C	0.67573333895193	21.75844145266709	29.69929344642302
906	C	-0.62256508600054	21.53304295854689	30.44020485358526
907	C	-1.83744487413867	21.63230770611744	29.75468831622765
908	C	-0.67002334946973	21.13836024019696	31.77554509538466
909	C	-3.04314952612362	21.32156778390068	30.36144984477038
910	C	-1.87601702561026	20.83633521915329	32.40701185471533
911	C	-3.07199460264027	20.90822774769715	31.69428944172283
912	O	-4.28330987314707	20.61132730060343	32.22970832813709
913	H	1.48800714983512	21.90858660882292	30.41446564386115
914	H	0.60661458047948	22.66712808304035	29.09500193683345
915	H	-1.83757862380672	21.94370016330662	28.71582893783961
916	H	0.25197939200964	21.05639219942800	32.34065486730790
917	H	-3.97415648564939	21.38552613191777	29.81156964563369
918	H	-1.88794018222680	20.53096537460116	33.44579180805452
919	H	-4.22267664873705	20.11395857212448	33.07887915195445
920	H	0.20413571330712	20.40963105886732	28.05690915355509
921	H	1.09367412672636	19.64818230209629	29.37805375813388
922	H	1.93387249139752	20.71772852353325	28.24746253496794
923	Cu	-5.72313228520458	22.78171362933093	34.28006922744122
924	O	-7.61263251920196	19.78976553540975	36.33164532226913
925	O	-7.15034332271448	22.21484043311904	35.39736863727558
926	H	-8.50395605742133	19.77504417697455	36.69994838694086
927	H	-7.48714721237172	20.69357672656785	35.92072973158069

Optimized atomic coordinates in Å – Q164E glutamate form – ^{1}RC

Q164E glutamate -3478.995726561916

N	-4.13330410766023	24.03679241323820	33.11599235672684
C	-4.56716672038605	24.37562753383888	31.75526736349551
C	-6.07669363892440	24.64044837568646	31.69048790402602
C	-6.89262757685337	23.39432149590414	31.72400506178975
C	-7.69469813592307	22.82667504212016	30.77750050670147
N	-6.91093924579210	22.58528121126109	32.85499457561179
C	-7.70330798933908	21.56078455830853	32.58477781441798
N	-8.19364583526885	21.67368271501909	31.33928367252028
H	-6.36581990659345	25.29888748793488	32.51530904594314
H	-6.30268832014971	25.17128789621329	30.76371579007633
H	-7.95254525922816	23.13643966384056	29.78023622433566
H	-7.93226582800757	20.74551139700923	33.25200244622175
H	-8.83175675705987	21.02717395426358	30.90096743485426
H	-3.20416877294713	23.63042544862766	33.09335557304017
H	-4.06536034590619	24.87761008231641	33.68074332080131
H	-4.31478445361016	23.54132896212254	31.10305078183108
H	-4.04016475451324	25.26035524596121	31.38211588902666
C	-1.23844599838912	22.37203193315202	39.17222107211445
C	-1.44521000520085	22.96951180318186	37.77903116634399
C	-2.83031006740606	22.86023529304796	37.23619981031591
C	-3.25282344161755	22.61353144600296	35.96176236969008
N	-3.98536123353803	23.03216952099441	37.96409138456983
C	-5.04289262904797	22.89456757679708	37.13856749677733
N	-4.63330913632465	22.64843025053109	35.90720585996831
H	-1.15718097878595	24.02656226164415	37.80021737779513
H	-0.78190514099345	22.48499769544276	37.06027757625255
H	-4.04649521690505	23.21410997399600	38.95419041629058
H	-2.65415697188805	22.41604133972456	35.09017822591232
H	-6.06526528759640	22.97333093676036	37.46435311176057
H	-1.52542806850563	21.31984889331988	39.18942622423389
H	-1.83089063961794	22.90048921742807	39.92262899242291
H	-0.18993828079122	22.45920903605015	39.45956196002397
C	-2.79218510195757	17.47356070579920	41.25807714636838
C	-3.43549224127968	18.86434882423458	41.21146456793350
C	-4.04237839713113	19.16855121266257	39.87835789114179
C	-5.34174279098119	19.45261123190250	39.54466490746717
N	-3.26401271223353	19.18830017918735	38.73563374080951
C	-4.08422625258074	19.47720486456720	37.74575579400847
N	-5.35056713474241	19.64383431621681	38.18267046871566
H	-4.20658868467942	18.94293848649410	41.98170987722623
H	-2.67926787048126	19.61948968679280	41.44983359148182
H	-6.23488191019344	19.53061739784135	40.14078106623738
H	-3.82009792841801	19.57895100165461	36.70598340137910
H	-6.13680357959236	19.87984791727261	37.58030766308931
H	-2.01066806605924	17.38035737520440	40.50266206925802
H	-3.53586750646426	16.69516538977513	41.07226313391963
H	-2.34239562425028	17.28898693360899	42.23628960608914
C	-1.93448586711510	16.36921160819643	32.92715299999258
C	-2.81892106565909	17.47429999864056	33.50104069896791
C	-4.12966846929269	16.93714942559410	34.05558331347170
C	-5.12388067872450	17.95231932623105	34.60538337942719
O	-4.92831412285107	19.19413926767706	34.43421243799447
O	-6.12258288425460	17.47756243583049	35.20961155091258
H	-2.27850262684091	18.01115508158851	34.28573621722218
H	-3.02282946054270	18.20633756689267	32.71887326313274
H	-4.66304885590178	16.38068549603544	33.27623105526101
H	-3.94025140916955	16.21232020163873	34.85281299651231

988	H	-2.43851571778631	15.84143068077746	32.11315958326440
989	H	-1.67416333867468	15.63100243291913	33.69050628286434
990	H	-1.00512135915398	16.78571765947616	32.53227386749867
991	C	0.92959174452558	20.56051787165482	28.80006781502453
992	C	0.63052933571144	21.76154387560941	29.70929959233518
993	C	-0.66903914184117	21.54502203712545	30.45426609256524
994	C	-1.88355728222159	21.64501837115271	29.76752475508315
995	C	-0.72109981870918	21.14187235823689	31.78718383961361
996	C	-3.08860577252746	21.31660874591789	30.36589618027759
997	C	-1.92762467772122	20.82169067800693	32.41086823611437
998	C	-3.12120400743044	20.88048559087679	31.69223521111651
999	O	-4.33177765748958	20.54553293919977	32.20302473055335
1000	H	1.44895504155295	21.90194024206938	30.41938264371025
1001	H	0.57057178990675	22.67061811807329	29.10471085174256
1002	H	-1.88302180657810	21.96697400036956	28.73180534903526
1003	H	0.19874040221678	21.06193335623913	32.35629213255336
1004	H	-4.01746786167387	21.38003194819351	29.81234679982039
1005	H	-1.93972528420387	20.50760041445608	33.44694945761857
1006	H	-4.29849365375296	20.06828166762122	33.06723101981190
1007	H	0.12892085526186	20.41633569020969	28.07202192278186
1008	H	1.01569424669536	19.64424235987985	29.38863355969422
1009	H	1.86417400649562	20.69945660905582	28.25067594666686
1010	Cu	-5.60505550525932	22.73588430449863	34.25465477499310
1011	O	-7.92054854861855	19.35341249611421	35.41266105297663
1012	O	-6.95817022077773	20.35524890771935	35.85438752117108
1013	H	-7.32214676461526	18.54207309346685	35.37196817449379
1014	H	-6.17374783316267	20.13604931779227	35.28087446469145

6. Optimized Atomic Coordinates for the Q164E mutant (glutamate form)

Optimized atomic coordinates in Å – Q164E glutamate form ^{1}TS1

Q164E glutamate -3478.980632326049

N	-4.22293014352969	24.01072647935685	33.13600741674092
C	-4.59999510675152	24.34353253789483	31.75225313904621
C	-6.10266960448215	24.59996372732502	31.68569779617055
C	-6.91180275340631	23.35158002093254	31.71732051870383
C	-7.70348975780599	22.80266406186117	30.75089019361965
N	-6.94767507484352	22.52530347888283	32.82885175604198
C	-7.74102320316365	21.50777165686345	32.53384810053351
N	-8.21646480591580	21.64488045508413	31.28845970802829
H	-6.39610717277430	25.25662051076059	32.51041869961183
H	-6.32977160248599	25.13025099894660	30.75879502115948
H	-7.95065299562415	23.13458123728798	29.75810325177855
H	-7.96638919568559	20.68636272005363	33.18957941810056
H	-8.85228005479189	21.00750939274717	30.83332397564154
H	-3.28550006838682	23.62397524886508	33.16348160963206
H	-4.20742907126186	24.85183252420484	33.70417329421524
H	-4.33101118831790	23.51232219729974	31.10351127176251
H	-4.06274743221915	25.22314482807256	31.38313832753679
C	-1.26508074925084	22.35035578791904	39.17353345476427
C	-1.47214352390947	22.94641034400459	37.78306004590562
C	-2.84170089576505	22.84374136969635	37.21889034184649
C	-3.22699796458672	22.65093614304660	35.92465189130512
N	-4.01284132961432	22.97332624266656	37.92687648399441
C	-5.04800098790362	22.85335211277732	37.07473599015884
N	-4.60087082415352	22.66745442286118	35.84730018716949
H	-1.18794997116923	24.00482190099505	37.80955357323838
H	-0.80418539033767	22.46343970236592	37.06718131558826
H	-4.09790463018455	23.11272562727550	38.92223180371179
H	-2.60492289809718	22.49148709402360	35.06198783611679
H	-6.07956076888234	22.88717935996971	37.37572387707016
H	-1.54719168988428	21.29673153840440	39.18977685315468
H	-1.86126544775785	22.87578537925283	39.92350967716012
H	-0.21752045443129	22.44227877851081	39.46246479724129
C	-2.79899717281364	17.44359200318415	41.25456039025831
C	-3.44949807655795	18.83301732908350	41.20763401848799
C	-4.05801855957542	19.14072067484230	39.87481638908090
C	-5.37334105738879	19.33626214578631	39.53532742837604
N	-3.27797213531195	19.23990971827764	38.73883501191504
C	-4.11445393231587	19.48777924495430	37.74850765960360
N	-5.39200773180199	19.55749506419644	38.17801233317935
H	-4.22157450604241	18.90507401918773	41.97753250903880
H	-2.69626764314010	19.59046519712159	41.44764954308958
H	-6.27275431541291	19.33384999154348	40.12764089191275
H	-3.85346817551156	19.62204357880180	36.71162226547783
H	-6.21418690326654	19.69360983649150	37.57242753486187
H	-2.01571399725701	17.35440928499962	40.50006904431071
H	-3.53872675597164	16.66184967187634	41.06724315697107
H	-2.34979401803434	17.26061224483894	42.23339498971648
C	-1.92367496433810	16.34755510198977	32.92438222459322
C	-2.81398435865583	17.44802285965303	33.49741154103168
C	-4.13544416781398	16.94543969600559	34.06114712238944
C	-5.09501126909703	18.02365020363479	34.56391263123263
O	-4.73454929388345	19.24251834914200	34.50985769700490
O	-6.19841289304365	17.64089314065287	35.02077802238404

1074	H	-2.27425160001191	17.98437657905097	34.28228471446204
1075	H	-3.01759369151496	18.17727737235256	32.71188767643455
1076	H	-4.67651807434542	16.36338255160662	33.30761092230196
1077	H	-3.96434936361094	16.25853734743333	34.89663050988928
1078	H	-2.42408866342583	15.81775916644790	32.10924099414690
1079	H	-1.66098612679619	15.61001297226584	33.68792343660697
1080	H	-0.99578740410893	16.76855636323187	32.53115412759110
1081	C	0.92694165770307	20.55432746871631	28.80372124360074
1082	C	0.62100214562919	21.75309270743416	29.71282197928889
1083	C	-0.67674545863626	21.53721944720802	30.45861631733315
1084	C	-1.89477899410433	21.63207380880260	29.77834914396657
1085	C	-0.71886210274189	21.14424970938617	31.79495860825299
1086	C	-3.09633335162666	21.31244196352548	30.38924984301876
1087	C	-1.92047971940796	20.83283585267192	32.43032537149073
1088	C	-3.11924809528401	20.89179432228372	31.72052095236021
1089	O	-4.32447166688141	20.57038280849365	32.25202231859433
1090	H	1.43789986089796	21.89659255938995	30.42400879879160
1091	H	0.55797333745922	22.66151766762611	29.10730117144582
1092	H	-1.90068548776862	21.94423007906367	28.73965258746388
1093	H	0.20561488218422	21.06754220885110	32.35693202869513
1094	H	-4.02931478479393	21.37210372933798	29.84224988923116
1095	H	-1.92641804248670	20.52618983692809	33.46862405978022
1096	H	-4.26758085378385	20.08758528105081	33.11529301409045
1097	H	0.12800574779941	20.40676908455340	28.07436677936057
1098	H	1.01645177919423	19.63812111114516	29.39197784917376
1099	H	1.86165141255793	20.69791217110032	28.25582174955706
1100	Cu	-5.66370532622888	22.59509464054768	34.25503807224356
1101	O	-7.49143700794192	19.72853475583224	36.24779396517824
1102	O	-6.70814058848258	20.95881006437747	35.29402249814676
1103	H	-7.12389696625740	18.94925676308261	35.75624132919813
1104	H	-5.94905958456704	20.36377472968244	35.04897751855571

Optimized atomic coordinates in Å – Q164E glutamate form ^{1}IC1

Q164E glutamate 1IC1 -3478.993261198784

N	-4.29635136560556	23.99736121277044	33.15408738251645
C	-4.61646266182401	24.32686328284369	31.74338642772923
C	-6.11009065588277	24.57995543856627	31.67669063100612
C	-6.91438535656948	23.33576796819117	31.70695154393708
C	-7.70522033441726	22.78687219529353	30.73867433654916
N	-6.94852509654899	22.51771305298016	32.80774997911632
C	-7.73940169885296	21.49395965663263	32.52391530347730
N	-8.21813761188341	21.63120396318328	31.28168297299133
H	-6.40471435748189	25.23569721442932	32.50170577315128
H	-6.33741784004137	25.11068372748210	30.74988187452880
H	-7.95647253722049	23.11908811561907	29.74706818394091
H	-7.94826014326090	20.67543228366078	33.18723138572540
H	-8.85566660220590	20.99278770790516	30.82990226217974
H	-3.35342838643274	23.62857156623636	33.22912660671141
H	-4.32355798044768	24.84263592359025	33.71678515476099
H	-4.33460213124991	23.49846672258623	31.09811108646356
H	-4.06047908744457	25.19487591853603	31.37970462264051
C	-1.27343588858752	22.33109908675710	39.16535539860156
C	-1.47977344601033	22.92563354066925	37.78033289084492
C	-2.83383328551356	22.83312272437855	37.19977212700918
C	-3.18268130838358	22.68260646629734	35.89241101087488
N	-4.01721200407489	22.98089416853737	37.88099194835079
C	-5.03084393055756	22.90883125635028	37.00155874021419
N	-4.54848349400964	22.74181855436650	35.78485654319869
H	-1.19638905793514	23.98460533170942	37.80926276139331
H	-0.80958557812995	22.44208955004899	37.06637416991486
H	-4.12363060366768	23.10257887715033	38.87681082942775
H	-2.53989071392486	22.52737325061259	35.04429999296190
H	-6.07014987700348	22.95839649111863	37.27079772654005
H	-1.55431779499461	21.27701832995160	39.18075204552364
H	-1.87074965866561	22.85542388302443	39.91556548327394
H	-0.22617787698210	22.42408003394652	39.45484364174600
C	-2.80246373412848	17.42109433731121	41.24232454635180
C	-3.45445574784127	18.80943985270340	41.19603176692269
C	-4.06524851478443	19.12702010235427	39.86853777007207
C	-5.38217951797120	19.32535060352229	39.53760842577031
N	-3.28946561749244	19.24719231769670	38.73216545130029
C	-4.13214513637851	19.51018931058996	37.74985599064778
N	-5.40753116538636	19.57064002131234	38.18534213942777
H	-4.22671462831248	18.87836701204598	41.96619550144112
H	-2.70094456524160	19.56647969020869	41.43714719933210
H	-6.27852891072770	19.31125785613579	40.13461217890493
H	-3.87835759161960	19.65672853815853	36.71277214641010
H	-6.24148584570230	19.69939730383273	37.58371152337671
H	-2.01860936098128	17.33335082766283	40.48805840929424
H	-3.54120047375793	16.63855110910461	41.05411103774882
H	-2.35353115166347	17.23799939626425	42.22121971457700
C	-1.92178890248333	16.33173542115044	32.91183345480120
C	-2.81231764085099	17.42904751711759	33.48428420043874
C	-4.13419940629939	16.92997893672478	34.04813118251489
C	-5.05980433098324	18.02240925182198	34.58570591131831
O	-4.65585869329368	19.22609330025993	34.55545208903937
O	-6.17036084182978	17.66377461936776	35.04459771020245
H	-2.27302838773371	17.96490103727048	34.26950026805955
H	-3.01743771599298	18.15985805852188	32.69977105759832
H	-4.69291734824484	16.37514050775728	33.28699098688217
H	-3.96505416417683	16.22140609058825	34.86597222853718

1165	H	-2.42123852970429	15.80181812141716	32.09598122680013
1166	H	-1.65857028737778	15.59395348151677	33.67504528326948
1167	H	-0.99411464640619	16.75416293328441	32.51929676212216
1168	C	0.92577195878650	20.54471146408229	28.79539936702471
1169	C	0.61797120977653	21.74241841745339	29.70510270111785
1170	C	-0.67918295829820	21.53687298285822	30.45692315840972
1171	C	-1.91197557676389	21.63408509964895	29.80625385084504
1172	C	-0.69222977203446	21.15295756621401	31.79799115150536
1173	C	-3.10104307129886	21.33739438446978	30.45650127421331
1174	C	-1.87801000864651	20.86493145117705	32.47027949028574
1175	C	-3.09517421526557	20.94076124801751	31.79528355075425
1176	O	-4.29151658924015	20.66840702782367	32.37877403179050
1177	H	1.43391043778341	21.88671334984132	30.41674933315475
1178	H	0.55468261687033	22.64983619136584	29.09767019323484
1179	H	-1.94371609193741	21.93232807487090	28.76395770493599
1180	H	0.24651381610371	21.07013842870048	32.33477878649414
1181	H	-4.04741577332267	21.40095351899315	29.93343951331797
1182	H	-1.85969062440140	20.56952178805828	33.51173699707159
1183	H	-4.22274508126708	20.11757204888489	33.20360945686815
1184	H	0.12727947109293	20.39667013641431	28.06547686440772
1185	H	1.01610225906259	19.62816478064737	29.38311384765948
1186	H	1.86043582224479	20.68976709109404	28.24813202448817
1187	Cu	-5.62964734657983	22.57240183389618	34.20801785985292
1188	O	-7.60093109151078	19.63753382193417	36.44136776236491
1189	O	-6.60625836155572	21.14666953014718	35.11310082246967
1190	H	-7.15560895928175	18.97448960250879	35.86057007241941
1191	H	-5.93076166313221	20.43802746973179	35.06458668678193

Optimized atomic coordinates in Å – Q164E glutamate form ^{3}IC1

Q164E glutamate 3IC1 -3478.985650982987

N	-4.31239999261014	23.99054833911582	33.15881638153676
C	-4.62723727267301	24.32206995694312	31.74521092782242
C	-6.12140292767460	24.57239756888258	31.67885003257598
C	-6.92105842069936	23.33003506661168	31.70642437801657
C	-7.70354925144870	22.80779229527139	30.71451173726176
N	-6.97057748683101	22.49721943944232	32.78659138669224
C	-7.76629522183762	21.48385063272254	32.47242496138371
N	-8.23075258164666	21.64701463923239	31.22830454956349
H	-6.41748509244781	25.22609197792040	32.50540442224931
H	-6.34964083961371	25.10451571840946	30.75315823357258
H	-7.93624554244926	23.16094589981606	29.72560570865515
H	-7.97959531340932	20.64868368638529	33.11186548073962
H	-8.86498340101786	21.01905406870910	30.75780592047687
H	-3.36438035744990	23.63719984351932	33.24206051883959
H	-4.36179281344765	24.83137914584946	33.72632202055879
H	-4.34287227101168	23.49559654956956	31.09850327931755
H	-4.07124056967496	25.19120374093910	31.38359045995226
C	-1.28122579811324	22.31782620232213	39.16351963607794
C	-1.48883006284564	22.91552865362788	37.77788890560186
C	-2.84965126207457	22.81522024534156	37.20637221345940
C	-3.21975680681074	22.62885156970847	35.90900178976067
N	-4.02299604041347	23.00674981993616	37.89567176243573
C	-5.04881976368947	22.93004457506431	37.02988681004538
N	-4.58705946317746	22.71339142636942	35.81315153826490
H	-1.20831513226933	23.97501854890767	37.80588293450360
H	-0.81820865644770	22.43560205361415	37.06206226956876
H	-4.11539878391055	23.16322757658907	38.88804083351512
H	-2.59153168121022	22.43636771871762	35.05762721111727
H	-6.08321137837295	23.02117440121092	37.31103091859493
H	-1.56003946819081	21.26323561960263	39.17675183181898
H	-1.87957006779318	22.83940472481295	39.91462523909135
H	-0.23413362378196	22.41226494627535	39.45333153622083
C	-2.80092172209173	17.40064899736631	41.23036650627314
C	-3.45418591875170	18.78479532730704	41.18690077061918
C	-4.06685227438290	19.11478210097303	39.87034146286718
C	-5.37163608122183	19.39693114036749	39.56052796106990
N	-3.29605845002445	19.20239576432392	38.72794764621879
C	-4.13296978664584	19.52930588734284	37.76035253474506
N	-5.39536575201711	19.66211182127412	38.21382248503679
H	-4.22665530043845	18.84953870186249	41.95768887437587
H	-2.70070781551265	19.54197021678167	41.42991905116865
H	-6.25955308226688	19.43109209448320	40.16923017293997
H	-3.88544770579866	19.66753266583746	36.72103137410511
H	-6.23183250652177	19.83599964200821	37.62511282636135
H	-2.01682832270222	17.31595959915555	40.47603031888706
H	-3.53821978726938	16.61693740719639	41.04045632006378
H	-2.35175163926825	17.21645125947978	42.20892438679581
C	-1.91719622741645	16.32986239445344	32.89780145951024
C	-2.80747832437511	17.42128102919608	33.47079257312578
C	-4.12936346799663	16.91945800306290	34.03403989942709
C	-4.99836497082674	18.01698741330676	34.64570349678775
O	-4.54239232807359	19.20527390293633	34.61605603117538
O	-6.09727410749873	17.69777854419165	35.15182021718316
H	-2.26928830403786	17.95605852025808	34.25666492411831
H	-3.01482476358131	18.15403739657960	32.68894706168768
H	-4.71959702374132	16.42164026845833	33.25747982414797
H	-3.96161660094183	16.16513057244845	34.81022133847407

1252	H	-2.41560557412816	15.80053542688256	32.08068269985662
1253	H	-1.65260957040881	15.59099887436506	33.65960570104347
1254	H	-0.99019820712707	16.75493456348699	32.50618476676267
1255	C	0.92262947539021	20.55669446322682	28.79022887210168
1256	C	0.61244597340102	21.75175882380898	29.70216429558181
1257	C	-0.68371250839285	21.54442006980133	30.45428198134591
1258	C	-1.92228965818654	21.63390177424824	29.81449756630349
1259	C	-0.68211755601615	21.15897805532419	31.79539916465064
1260	C	-3.10349231495829	21.32904752424760	30.47664789999389
1261	C	-1.85840262191815	20.86203169155730	32.47839237821970
1262	C	-3.08190906133655	20.93118200453594	31.81476302599054
1263	O	-4.26862908059599	20.64785992391035	32.41271680153245
1264	H	1.42754864609868	21.89678825651007	30.41441650860648
1265	H	0.54771975183573	22.65968085361416	29.09530334332523
1266	H	-1.96606704792358	21.93175870304179	28.77252946312131
1267	H	0.26247262721873	21.08123919459998	32.32256188288241
1268	H	-4.05525074089813	21.38650184160273	29.96273759338631
1269	H	-1.82837277496661	20.56388026121044	33.51878364158493
1270	H	-4.17687499310190	20.10196532085356	33.23800862785708
1271	H	0.12449215929741	20.40857202487524	28.05989938458614
1272	H	1.01468241661161	19.63909270342739	29.37612884136795
1273	H	1.85705617947408	20.70467360751979	28.24337404901543
1274	Cu	-5.64808094196083	22.51599825672074	34.21125045851252
1275	O	-7.58396014276821	19.82470948745678	36.57690290452144
1276	O	-6.57563563384779	20.92179835235945	34.85359232412693
1277	H	-7.20713759479383	19.12033162112541	36.00607407565006
1278	H	-5.84518241353801	20.27137135753264	34.95210990350673

Optimized atomic coordinates in Å – Q164E glutamate form ^{1}IC2

Q164E glutamate 1IC2 -3479.007460588682

N	-4.27495014573058	23.96981827917534	33.14634237740479
C	-4.60243515716040	24.29937308052673	31.73330435305681
C	-6.09764969098835	24.54444144131058	31.66437954139156
C	-6.89589072615297	23.29489850116240	31.69326780238590
C	-7.68797358237322	22.72332189949653	30.73813424198659
N	-6.91523674322030	22.49033777601815	32.80057543925770
C	-7.68845565189240	21.44949202829051	32.53826080821837
N	-8.17435171652426	21.56342264053525	31.29524199776725
H	-6.39701042641758	25.19833942013664	32.48890814909786
H	-6.32646172268387	25.07410232532608	30.73726696635823
H	-7.94430982943492	23.03375111305714	29.74094544734675
H	-7.89713466428456	20.62959655061728	33.20233096912995
H	-8.79934267812570	20.90671211894178	30.85179963702291
H	-3.36450973211650	23.52413827022292	33.20325815970212
H	-4.20985695841716	24.82649955598103	33.68909890224779
H	-4.31623545573379	23.47243719718243	31.08797672941096
H	-4.05007535955947	25.16931727000312	31.37103116801767
C	-1.26014341702532	22.32063369603136	39.15997142847569
C	-1.46736380143564	22.91354111183112	37.77640590503682
C	-2.82618560755024	22.80315845367350	37.21280302389239
C	-3.20909286627002	22.67155782371803	35.91318606148059
N	-3.99025099271377	22.96554689819498	37.92495006750558
C	-5.02379772904301	22.93708025378938	37.06926317748423
N	-4.57518358889882	22.76900007581596	35.83874239403475
H	-1.19139691603560	23.97427794565475	37.79935562007589
H	-0.79401740927529	22.43465971846415	37.06198467639125
H	-4.06908090087811	23.08421606827690	38.92372070759805
H	-2.58997021025596	22.51275381753804	35.04821288030949
H	-6.05571145138598	23.03592742219872	37.35699784234810
H	-1.53532850671703	21.26507401935769	39.17478881222632
H	-1.86133762221232	22.84153268403065	39.90926575643278
H	-0.21378497354852	22.41924388215533	39.45106260995849
C	-2.76574298157853	17.40211125545029	41.23394359476948
C	-3.42479679401980	18.78612930565720	41.18689745952859
C	-4.03407218196275	19.09770696697022	39.85819179625841
C	-5.35313560458479	19.29473499750878	39.53755193035317
N	-3.26749434482387	19.19920962216923	38.71381503195170
C	-4.11717414153713	19.45019399733525	37.73475093900861
N	-5.38847110189047	19.51919523241873	38.18264577782316
H	-4.19887341601248	18.85263676143711	41.95521883244355
H	-2.67517145601327	19.54683218212909	41.42937244246927
H	-6.24425926949534	19.29059226189649	40.14219504464837
H	-3.87366551658071	19.58391034442878	36.69291572183365
H	-6.21766387290202	19.65552503294038	37.59000282764124
H	-1.98033293322738	17.31873343599091	40.48089011204548
H	-3.49995599627765	16.61560129796967	41.04449935519358
H	-2.31729967825724	17.22129679187263	42.21349340311721
C	-1.86662983899631	16.31881815803903	32.90461141639211
C	-2.76575312922552	17.41340470852183	33.47703146892938
C	-4.09311277607837	16.93534066716685	34.04603284424809
C	-5.02265089452492	18.05505972681218	34.53324471226798
O	-4.63978220216474	19.25694661541050	34.45237399456776
O	-6.13265779553250	17.69958260619651	35.00925487151238
H	-2.22729481173786	17.94995153596667	34.26283784649359
H	-2.97157983709600	18.14111835169861	32.69020811795536
H	-4.64803912037986	16.35632064036847	33.30001636789945
H	-3.93630479442319	16.25697943692570	34.89127857377971

1339	H	-2.36198724537945	15.78631899233624	32.08790756787479
1340	H	-1.60054658706646	15.58228291639517	33.66818170654648
1341	H	-0.94082817705554	16.74624674137148	32.51360465150661
1342	C	0.96428492345453	20.54779445006066	28.79309267301860
1343	C	0.64866800760839	21.74355284839430	29.70241619411512
1344	C	-0.64839459288046	21.52359596746112	30.44795139665505
1345	C	-1.86573831851186	21.62362238158820	29.76700881303962
1346	C	-0.69331114751588	21.13316935628695	31.78486642003479
1347	C	-3.06977102361694	21.31829101125591	30.37980343543467
1348	C	-1.89760044530662	20.83637899588849	32.42233334488655
1349	C	-3.09825062813630	20.90839034114030	31.71510592294830
1350	O	-4.30578370326570	20.61890274019943	32.25412223654523
1351	H	1.46367335019696	21.89117205204514	30.41504157388023
1352	H	0.58081865025488	22.65206776888028	29.09760767381212
1353	H	-1.86919728220370	21.93165228280444	28.72703394812289
1354	H	0.23065950183136	21.05054745076000	32.34693030369799
1355	H	-4.00236076972792	21.38358093742085	29.83254160439273
1356	H	-1.90545913919248	20.53405629134856	33.46178304683091
1357	H	-4.25723610897626	20.10894212481286	33.10963241055072
1358	H	0.16772864738268	20.39555174024600	28.06196700685087
1359	H	1.05868572011523	19.63170368959911	29.38077707254940
1360	H	1.89907175003045	20.69800908728838	28.24719803061958
1361	Cu	-5.73263279656797	22.79136376636022	34.29024334776206
1362	O	-7.49106818019983	19.68197206090890	36.27327152610961
1363	O	-7.20193547183093	22.24948571354350	35.35996047335591
1364	H	-7.00287794906365	19.02044282424778	35.71857979106518
1365	H	-7.39529107698066	20.55894255530018	35.83264319350260

Optimized atomic coordinates in Å – Q164E glutamate form ^{3}IC2

Q164E glutamate 3IC2 -3479.012337027827

N	-4.26554526181820	23.97980439915450	33.14123789396618
C	-4.59409910261557	24.30940950241911	31.72898174747765
C	-6.08956179937296	24.55733937257983	31.65978892399796
C	-6.89006626426681	23.30938293947347	31.68918371433046
C	-7.68264486058758	22.74121965113784	30.73183213718897
N	-6.91195002859168	22.50481932223599	32.79554731949775
C	-7.68912817132262	21.46766844709395	32.53155315078369
N	-8.17447136302133	21.58329006679529	31.28797005442557
H	-6.38777500888677	25.21217080812285	32.48399245416403
H	-6.31733056064937	25.08703411428316	30.73235071996047
H	-7.93873919562714	23.05430489672237	29.73537520367097
H	-7.90131444529185	20.64813859959777	33.19523144679249
H	-8.80176724534298	20.92929872091432	30.84393828403573
H	-3.35223131152527	23.53997877429240	33.19759259907516
H	-4.20566040877657	24.83627131151539	33.68465928751409
H	-4.31007239811551	23.48150379514660	31.08380824032006
H	-4.04135620232619	25.17911018620732	31.36599640741547
C	-1.25680233785553	22.32800002642683	39.15679949578197
C	-1.46291425055982	22.92095703024290	37.77223946946165
C	-2.82390950747166	22.81011999687408	37.21319377799178
C	-3.21981623575977	22.65837308692807	35.91954654062071
N	-3.97905882079513	23.01199267487398	37.93023143142972
C	-5.01996110409346	22.98864622004405	37.08403051983444
N	-4.58463989448944	22.78509057726943	35.85305565577171
H	-1.18539905838210	23.98124127833100	37.79305586570143
H	-0.79040972232317	22.44051239218992	37.05807698573708
H	-4.04638702238095	23.15765733091400	38.92627103004511
H	-2.61173806017627	22.47161991266908	35.05239681696832
H	-6.04679664848314	23.11586620659885	37.37727685767236
H	-1.53392558010810	21.27304451116296	39.17207800141639
H	-1.85707844761670	22.85034891481207	39.90579113013121
H	-0.21023430705428	22.42480609758284	39.44794588263528
C	-2.77169479656156	17.41323430759542	41.23290548280642
C	-3.42813044545075	18.79835890749291	41.18517294251848
C	-4.03594026204214	19.10878850578081	39.85629766109003
C	-5.35958220018460	19.26774881059635	39.53414065081115
N	-3.27079142983684	19.23518690291773	38.71387047221987
C	-4.12617250667537	19.46340378075947	37.73384200435153
N	-5.39951072350240	19.49482398359061	38.18025903991199
H	-4.20207455717018	18.86647779020618	41.95342168841918
H	-2.67700357162946	19.55762862951974	41.42737434313613
H	-6.25118957225628	19.23475623138889	40.13719441229799
H	-3.88508822415656	19.60624387178997	36.69274366730346
H	-6.23549963189211	19.59576758244454	37.58988216257331
H	-1.98631544807695	17.32804563339178	40.47989290412302
H	-3.50734212744021	16.62801142975118	41.04376478969489
H	-2.32365058454615	17.23203106820159	42.21261143328208
C	-1.87391932811522	16.32444703307548	32.90414611424838
C	-2.77057641063935	17.42037815232828	33.47568397291749
C	-4.09463387162880	16.93047063624594	34.04085475659011
C	-5.03679282388382	18.03375058258799	34.53600090391491
O	-4.68138528469759	19.24268219444291	34.44395054222587
O	-6.13244445454135	17.65484016902984	35.02856985127883
H	-2.23289986551349	17.95761644797147	34.26165881578905
H	-2.97595138405507	18.14899752211020	32.68968181983443
H	-4.64323179584335	16.35227162821994	33.28935152823949
H	-3.93212557676162	16.24585212370948	34.87982320613794

1426	H	-2.37017838622531	15.79251426842699	32.08767897846730
1427	H	-1.60927604298102	15.58779894946788	33.66804655926063
1428	H	-0.94721493083367	16.75001297012728	32.51298541891446
1429	C	0.96517430606989	20.54627070134284	28.79091761748141
1430	C	0.65165951461192	21.74320029775066	29.69979450395911
1431	C	-0.64637845568008	21.52494837019845	30.44505638249968
1432	C	-1.86450814078839	21.62793278304327	29.76606328001336
1433	C	-0.69046447584655	21.12978200319833	31.78080940322047
1434	C	-3.06793838285949	21.31994173168829	30.37916622315418
1435	C	-1.89403147062978	20.83023649176230	32.41851442684538
1436	C	-3.09529816568509	20.90397806876964	31.71265416817803
1437	O	-4.30273311204611	20.60997720651948	32.25014760217130
1438	H	1.46673239615789	21.88981999690563	30.41249072193090
1439	H	0.58555885840091	22.65152415293439	29.09451059079262
1440	H	-1.86941310502191	21.93975629494200	28.72722105919778
1441	H	0.23399496944790	21.04481907629584	32.34171787103803
1442	H	-4.00101952573629	21.38727341586031	29.83300809727370
1443	H	-1.90050659869070	20.52378871376802	33.45680958647795
1444	H	-4.25773967694097	20.09657703933108	33.10316096284529
1445	H	0.16839628841000	20.39517093964956	28.05979685372121
1446	H	1.05782633733740	19.63027980014846	29.37902847125914
1447	H	1.90027975079527	20.69449842829906	28.24503234527459
1448	Cu	-5.72507521076693	22.77521266687005	34.29440264233043
1449	O	-7.52881868460342	19.58270751400526	36.29656411346036
1450	O	-7.12436681948766	22.13487033889822	35.40054896258741
1451	H	-7.02125041082301	18.92682991739537	35.74973848119187
1452	H	-7.38980013878379	20.46361513257850	35.87772396681213

7. Optimized Atomic Coordinates for the Q164E mutant (glutamic acid form)

Optimized atomic coordinates in Å – Q164E glutamic acid form [1]RC

Q164E glutamic acid 1RC -3479.445479873484

N	-4.12357839406181	24.02732891117029	33.12839827688099
C	-4.55360169817580	24.36545880792439	31.76526620154767
C	-6.06361435268245	24.62364236768478	31.70556141557700
C	-6.87323778097657	23.37510902971711	31.73980058772261
C	-7.67266392577580	22.81607378358780	30.78604371663556
N	-6.89223696262942	22.55674956351176	32.86398898591388
C	-7.68317630688323	21.53526929529422	32.57916592583815
N	-8.17161497218642	21.65798410846724	31.33497752854838
H	-6.35305998893711	25.27967874730669	32.53223418262233
H	-6.29477420319263	25.15476158855131	30.78030072132497
H	-7.92830888972982	23.13652603868115	29.79161664179696
H	-7.91516785140247	20.71219238854724	33.23169814611046
H	-8.80881201217498	21.01455426737146	30.89063648094574
H	-3.19074849746619	23.62916319549277	33.11233499837540
H	-4.06754864818806	24.86782700687329	33.69492530783815
H	-4.29773990432345	23.53353257338690	31.11172578890908
H	-4.02950308200226	25.25181966454667	31.39215628265373
C	-1.19238660291112	22.36583096134372	39.16908013696227
C	-1.40636275126402	22.96519278226951	37.77544924204715
C	-2.79237281841523	22.85258658391650	37.23276274421259
C	-3.22092729605493	22.55601294282263	35.97038229134029
N	-3.94364141847597	23.08937813044846	37.94895535271964
C	-5.00392306502332	22.93970180221800	37.12998603282826
N	-4.60111614057862	22.62223988532594	35.91226138671045
H	-1.12281143955506	24.02333701370737	37.79760737522178
H	-0.74282266444025	22.48400413121915	37.05473983962514
H	-4.00078377373907	23.32454998352121	38.92811291553643
H	-2.62794683947657	22.30679119079875	35.10834341728047
H	-6.02384899729762	23.06236938571312	37.45004087165472
H	-1.47471251506878	21.31244609470887	39.18567874556729
H	-1.78471913774396	22.89057399321359	39.92196132391267
H	-0.14340685481762	22.45717226480031	39.45329608849855
C	-2.71831294436405	17.45771423358812	41.25278473341033
C	-3.36730059955279	18.84465531871145	41.21016642809438
C	-3.98543893260320	19.16766015227013	39.88819414356810
C	-5.26822602842701	19.54863253855823	39.59303021905847
N	-3.23480090218487	19.15193837099309	38.72656528976338
C	-4.05395639880863	19.51772935611583	37.76291686847284
N	-5.29453229852935	19.76623999070989	38.23557372750910
H	-4.13628529833819	18.91828835529687	41.98311015988341
H	-2.61178930030737	19.60127034677615	41.44758469706989
H	-6.13877997106989	19.68249353608887	40.21197636316704
H	-3.80216921324818	19.62656662028113	36.72130845137994
H	-6.07851617242696	20.07710355497335	37.67533492468392
H	-1.93871709357175	17.36897694342585	40.49480412217618
H	-3.45923141253862	16.67628119932111	41.06815141010890
H	-2.26480798135695	17.27377477458206	42.22910070859093
C	-1.88161637773573	16.36881205572105	32.91768860281861
C	-2.76883374185749	17.46892945349308	33.49571603973454
C	-4.06362447981175	16.88498105559302	34.04233241332012
C	-5.06235509425186	17.85803597391891	34.59367015843740
O	-5.00771584344950	19.07839756960721	34.48042734432337
O	-6.05571724952059	17.25144862477051	35.22562349136538

H	-6.73900668969620	17.90915139699004	35.51721344467500
H	-2.23428780996502	18.01089483924856	34.28006564642607
H	-2.98191951022564	18.20461294952761	32.71945495839173
H	-4.58987361494725	16.32850625071065	33.25792723056095
H	-3.86011846271964	16.15274921469405	34.82877703517727
H	-2.38566453094372	15.84018922915234	32.10482670114654
H	-1.61582497086706	15.63096779010268	33.67888221171386
H	-0.95581045624281	16.78968850519439	32.52074808681206
C	0.95139052372347	20.57835969082656	28.78772130746325
C	0.65003792522522	21.77631253046410	29.69920984403237
C	-0.64460559574899	21.55580175021301	30.44836292120729
C	-1.86153551387237	21.64958233643247	29.76517941960843
C	-0.68699322596810	21.16037571028441	31.78334303042839
C	-3.06400212019793	21.32576850291586	30.37109019275095
C	-1.89008556278969	20.84397009822813	32.41407768486270
C	-3.08404882504122	20.90306563757207	31.69984660737633
O	-4.29491811592338	20.57156474804789	32.22546940407573
H	1.47006056481055	21.91918865157967	30.40688942537739
H	0.58429247074435	22.68584484589966	29.09590868056766
H	-1.86528434796022	21.96455456762938	28.72751171592249
H	0.23589401686094	21.08439036345467	32.34763000682284
H	-3.99621747224663	21.38484543133240	29.82309195764683
H	-1.89888876518295	20.53412381535167	33.45179045638243
H	-4.22665815561747	20.14789122455466	33.10014861084348
H	0.14910053423787	20.43171007489498	28.06194564358004
H	1.04330680605436	19.66162314781541	29.37473079248219
H	1.88358420581156	20.72213238797368	28.23568233931285
Cu	-5.58015605796248	22.69847951857225	34.26262123257133
O	-7.82354995296967	19.17032727773091	36.00890188936946
O	-7.05389845506939	20.39762971122383	35.88495670057056
H	-8.55051103133324	19.31739510781615	35.38004710847697
H	-6.31090846237028	20.10865082962089	35.29944900205872

Optimized atomic coordinates in Å – Q164E glutamic acid form ^{1}TS1

Q164E glutamic acid 1TS1 -3479.438548097527

N	-4.20929597552451	23.98568055069931	33.11246382282605
C	-4.61326638535706	24.31675676050360	31.73594704864071
C	-6.11628926174420	24.57535320209544	31.69274480681453
C	-6.92639591009134	23.32877409890710	31.73983605177023
C	-7.73447474172375	22.77732235125541	30.78914722974686
N	-6.94346895532921	22.50645351654703	32.85631034043203
C	-7.74233555637816	21.48876373849931	32.57671041767908
N	-8.23817875032575	21.62161289339219	31.33886046010132
H	-6.39559193177260	25.23419794379794	32.52066096022713
H	-6.35728402010566	25.10404014044294	30.76848770831954
H	-7.99775857738464	23.10609739851832	29.79948837551256
H	-7.96297536957036	20.66726800140910	33.23364568470832
H	-8.88228132126088	20.98328756798369	30.89684134246502
H	-3.27453022094072	23.59158841014951	33.11975163347587
H	-4.17110824271876	24.82997007475642	33.67509327624170
H	-4.35764280545421	23.48432975062654	31.08492180477052
H	-4.08090095512730	25.19476667256809	31.35649799761626
C	-1.16369162405401	22.33385982992604	39.10747125318996
C	-1.39227147954919	22.92822315574006	37.71713724319771
C	-2.77453748712115	22.82526419206384	37.17557923409064
C	-3.18557680069264	22.61233020826919	35.89175567810763
N	-3.93328030548740	22.96994718394575	37.90165899042827
C	-4.98500693339975	22.84033240197854	37.07151568746621
N	-4.56271311215887	22.63180093559574	35.83768497906403
H	-1.10638138474464	23.98611995280647	37.73595119336080
H	-0.73673935574969	22.44301000102966	36.99157143927076
H	-3.99993901578532	23.12550681416331	38.89610728762205
H	-2.57966336656082	22.44127235061098	35.02039521821512
H	-6.01034387519093	22.88756068494581	37.39184955335198
H	-1.44706666610194	21.28085188171717	39.13041357466815
H	-1.74704889361084	22.86174318886865	39.86567648925016
H	-0.11155042507595	22.42476071091686	39.37951751269987
C	-2.67178420188094	17.43383629579592	41.22296840187992
C	-3.32032508532219	18.82305807470109	41.18348808012291
C	-3.95315914342397	19.14423745292129	39.86882762203383
C	-5.27778763285562	19.34008969501992	39.57485418739547
N	-3.20587849366272	19.27419005782658	38.71458631619939
C	-4.06794028462990	19.54327174230336	37.75494556626502
N	-5.33372572624363	19.59374631598121	38.22514654850064
H	-4.07964098213422	18.89661952557482	41.96575526487686
H	-2.56087441661180	19.57852399925702	41.41010811181855
H	-6.16044341049474	19.32070437375875	40.19101610485968
H	-3.82913183460429	19.71911843029040	36.71994847774265
H	-6.16996379906158	19.75371953720968	37.67358784866967
H	-1.90056371987431	17.34184825464811	40.45614986970744
H	-3.41562114103647	16.65281016600493	41.04907053651963
H	-2.20745900550470	17.25224202253320	42.19471055366871
C	-1.93064980955513	16.31907785703505	32.88224992050855
C	-2.80928796096976	17.42113712986360	33.46651430512823
C	-4.11747912822940	16.90023793238349	34.04691316253506
C	-5.01337013203202	17.97455712140053	34.59300363007522
O	-4.76282149025405	19.17652592279325	34.52166341111047
O	-6.10708536710955	17.52303559016463	35.17044263948545
H	-6.66928535543709	18.28914193254163	35.50811493794927
H	-2.25947476318140	17.96032154543250	34.24189713090346
H	-3.02778939959064	18.15260480844866	32.68696091807853
H	-4.69532572180191	16.35687801172448	33.29143517264295

1605	H	-3.94059657113647	16.18309101535973	34.85448685422201
1606	H	-2.44460875989516	15.78851716363496	32.07652065408786
1607	H	-1.65708925819235	15.58302784398471	33.64275664238779
1608	H	-1.00910602083700	16.73754242790436	32.47371012103871
1609	C	1.18325645091626	20.54899923018381	29.14906358626251
1610	C	0.56952898391028	21.81007157939099	29.76946552242422
1611	C	-0.74612902547124	21.54095262657797	30.45449370634362
1612	C	-1.95353186687062	21.62350164315662	29.75672501193717
1613	C	-0.79527327795568	21.16048445843323	31.79548936599353
1614	C	-3.16316010869188	21.34206392975404	30.37427625481062
1615	C	-1.99776764161114	20.85872977537367	32.42508434107190
1616	C	-3.18819377902548	20.93713612679779	31.70653185984423
1617	O	-4.40080639102669	20.63834432435878	32.24459422662630
1618	H	1.27386275496677	22.23423168773686	30.49015267835953
1619	H	0.42597641527628	22.56072971677715	28.98774567367369
1620	H	-1.94749785627498	21.92732653872575	28.71580867517942
1621	H	0.12576716793630	21.09664007989193	32.36436068578251
1622	H	-4.10848739912736	21.41206375969136	29.85047257984116
1623	H	-2.01498840753997	20.55857612035842	33.46532367418888
1624	H	-4.31077980725099	20.18021748080122	33.10204033755785
1625	H	0.51217143747121	20.11739616906345	28.40278858834737
1626	H	1.36683796680221	19.79036634877593	29.91354377187816
1627	H	2.13379503240070	20.77601569081587	28.66057510125236
1628	Cu	-5.62785027950699	22.60893016857018	34.24970114206675
1629	O	-7.42240236761929	19.62246531889509	36.13041277065327
1630	O	-6.71696508502186	20.92788475119015	35.34045398896798
1631	H	-8.33132461972697	19.76312077965529	35.82737120527817
1632	H	-5.94917775330202	20.38132952878838	35.04904850490131

Optimized atomic coordinates in Å – Q164E glutamic acid form ^{1}IC1

Q164E glutamic acid 1IC1 -3479.486191745393

N	-4.33647910610426	23.97072918846051	33.17855226823652
C	-4.63812648920648	24.29757593133148	31.75562178918061
C	-6.12524035287603	24.53606575711893	31.69212073702378
C	-6.91344558463996	23.29262864954687	31.72248020838473
C	-7.71142342291342	22.69136046555350	30.79888678621605
N	-6.90275021114289	22.50894707937169	32.85035070614222
C	-7.66358436541159	21.44271041166143	32.62067919557665
N	-8.18027611612020	21.54215265649427	31.39692211976632
H	-6.42416939747331	25.18833157689691	32.51807943644038
H	-6.35877923794692	25.06534494341149	30.76657247357517
H	-8.01134007365515	22.99370181368249	29.81143804583584
H	-7.82170676228951	20.62480675188997	33.29734994681856
H	-8.81577669241496	20.87481042684218	30.98308928774769
H	-3.38666590578366	23.61916265592262	33.27667561086318
H	-4.40423450382613	24.81121506255490	33.74888683470441
H	-4.33933337580757	23.47435500322847	31.11279479084072
H	-4.07240834111452	25.15876734250214	31.39619882107277
C	-1.25578547335940	22.32361703778622	39.17036672897304
C	-1.47075994235198	22.92016823221370	37.78003913766091
C	-2.81141004732507	22.84218633207948	37.15461741295311
C	-3.09506810514366	22.63995809367350	35.83586263325876
N	-4.02209994418345	23.06819202416585	37.76384830928624
C	-4.99647302804320	22.99684811251344	36.84944966856435
N	-4.45250700672892	22.75607685216858	35.66526441555185
H	-1.19568859586483	23.98091968917518	37.81399199501228
H	-0.79496536587860	22.43907038018357	37.07010198065616
H	-4.17173118011366	23.24953909764715	38.74635630859451
H	-2.41997237773172	22.42343607887454	35.02789587304982
H	-6.04534911503758	23.10061081380677	37.05897896179477
H	-1.52721296544105	21.26737114765742	39.18530916442793
H	-1.85633943611554	22.84190905444836	39.92198936610647
H	-0.20931603831847	22.42558100016555	39.45800026225276
C	-2.73763827403923	17.39845737744004	41.24564049958243
C	-3.40162722795437	18.78026252679426	41.20173606999217
C	-4.01734377585756	19.09575895061638	39.87899059774472
C	-5.34763400768097	19.19219279919826	39.56152232343249
N	-3.26157266740235	19.29316949633696	38.74084035082625
C	-4.12795181741154	19.50193748515502	37.76668995046381
N	-5.40109326424710	19.45361709937528	38.21476833879161
H	-4.17288445364731	18.84134186721711	41.97322687913203
H	-2.65354420531462	19.54282170161018	41.44235151047668
H	-6.23589534624794	19.09605905962198	40.16262060186970
H	-3.89251909114094	19.68337312892338	36.73090280668764
H	-6.24831401505409	19.51130370365199	37.64310373209938
H	-1.95414582273563	17.31830093803381	40.48973710313983
H	-3.46972799340452	16.60957416699604	41.05801774630011
H	-2.28541110218071	17.21850084962787	42.22355733830342
C	-1.86187238393621	16.32421024299253	32.91265551775388
C	-2.76405720953275	17.41668088358362	33.48952457681320
C	-4.08302666617108	16.91149268621699	34.05723958529679
C	-5.02364892915910	17.98310400990665	34.61186500130100
O	-4.67912533414753	19.20555350214643	34.53616272055373
O	-6.09459386385042	17.58666300605174	35.12773278618158
H	-7.14021663339028	18.67402558495563	35.91293659851105
H	-2.22608361957515	17.95533040067559	34.27413040167144
H	-2.97591937852083	18.14550405657334	32.70434923571715
H	-4.64448614473208	16.36277193368132	33.29347982782633

1693	H	-3.90866225466377	16.19525765525484	34.86650350130093
1694	H	-2.35796992422028	15.79066429953530	32.09729841466365
1695	H	-1.59084317751142	15.58825060482044	33.67462123299160
1696	H	-0.93895417870598	16.75504925747144	32.51900842497117
1697	C	0.94106671565750	20.56585484513492	28.79503151048957
1698	C	0.62445260354748	21.75923595973650	29.70575569858865
1699	C	-0.66757389028568	21.54816306607044	30.46217830514822
1700	C	-1.90762404492107	21.66884723126515	29.83149113998082
1701	C	-0.66128378699372	21.14367296994092	31.79768450805922
1702	C	-3.09052454640309	21.38790799284467	30.50193351023322
1703	C	-1.83855742693860	20.87280301243924	32.48866222068343
1704	C	-3.06394582185889	20.99032042310561	31.83805101336730
1705	O	-4.25773774216591	20.76748747728468	32.46326985352671
1706	H	1.44058875796310	21.90955933768847	30.41575352273107
1707	H	0.55212385630346	22.66637898832778	29.09906629715956
1708	H	-1.95213587696716	21.97594925743483	28.79244549925432
1709	H	0.28593274276493	21.03727637064335	32.31459846575839
1710	H	-4.04499548149272	21.46822948302919	29.99633673300865
1711	H	-1.80820740035473	20.56441081547847	33.52598380684650
1712	H	-4.19121666779237	20.12660240136115	33.21924176443927
1713	H	0.14254361855497	20.41136079835436	28.06627201622042
1714	H	1.04054104412142	19.64961891287308	29.38178123418744
1715	H	1.87331271556036	20.71964320376081	28.24635387539334
1716	Cu	-5.50775225523544	22.59159168511437	34.13001100163257
1717	O	-7.69002369663878	19.30028828404908	36.45638281508304
1718	O	-6.37287129704819	21.26376029390010	35.00102974183918
1719	H	-7.60924322947465	20.14944861696650	35.99128083068665
1720	H	-5.73068534509953	20.50234628068553	34.93847022369096

Optimized atomic coordinates in Å – Q164E glutamic acid form ^{3}IC1

Q164E glutamic acid 3IC1 -3479.471098336169

N	-4.27674417977339	24.00156108407636	33.16856749824326
C	-4.60663667426847	24.32787871439811	31.75842750130019
C	-6.10140089282776	24.57403000609931	31.69493683287871
C	-6.90050601161422	23.32536997390732	31.72929630594661
C	-7.69113719290356	22.77191190428053	30.76295180423303
N	-6.92921406382947	22.50891125985858	32.82967670732938
C	-7.71718103110089	21.48114600236570	32.54692426287718
N	-8.19836161573614	21.61485829346777	31.30632224908165
H	-6.39699941081180	25.22981600091162	32.51951877927193
H	-6.33330626223947	25.10194277617383	30.76810295321307
H	-7.94684940813085	23.10213745941610	29.77191679387881
H	-7.94128198060168	20.65311582327177	33.19661508335194
H	-8.83784498217292	20.97575342392001	30.85740313077761
H	-3.32369629218463	23.65741083611108	33.23941410519677
H	-4.32124497868108	24.84591773395044	33.73232614884808
H	-4.32228744142320	23.49980448538820	31.11404707906954
H	-4.05464368987588	25.19671878622466	31.39233051805037
C	-1.23643981129582	22.36005684414810	39.17565608502112
C	-1.44849847830388	22.95032877078922	37.79233094319151
C	-2.80490162989648	22.84660073290716	37.22308252844552
C	-3.15763450168461	22.68247433952228	35.91921533372660
N	-3.98471761213619	23.01474807546727	37.90623454663348
C	-5.00146703777799	22.95244889195709	37.02991465549541
N	-4.52060691242395	22.76759195649641	35.81302140014984
H	-1.17092884586597	24.01093710153129	37.81443865848346
H	-0.77868746526308	22.46990833238599	37.07593928003609
H	-4.08607986359740	23.15554166277151	38.90037096192627
H	-2.51960739509166	22.51059696007993	35.07052400746645
H	-6.03952866640536	23.04050543187632	37.29832808522213
H	-1.51239910956551	21.30504501808926	39.19352612321796
H	-1.83434522395411	22.88296824728978	39.92645413684005
H	-0.18879728077134	22.45831031474718	39.46247459540403
C	-2.73802711217916	17.44670059265335	41.26467732649310
C	-3.39563835331517	18.83028062383313	41.21766356287509
C	-4.00706527261210	19.13469439629332	39.89186393880149
C	-5.33625036905282	19.17907258788430	39.56038531854639
N	-3.24712052503574	19.35832624551013	38.76214114185656
C	-4.10944855950580	19.53379073642830	37.77920068317819
N	-5.38509407728350	19.43492478333623	38.21295591537236
H	-4.16615982767865	18.89703849285777	41.98938605186768
H	-2.64601992168808	19.59188736535248	41.45547372973861
H	-6.22702738062523	19.04855426311014	40.15106030939113
H	-3.85989862725416	19.74191769699556	36.75256147840399
H	-6.23443632665540	19.47155588621075	37.64662556353817
H	-1.95536827839470	17.36120666723159	40.50842364013773
H	-3.47358481931833	16.66054618964591	41.07960816241719
H	-2.28586285928214	17.26731617241405	42.24285694257358
C	-1.87259441636100	16.34708553560841	32.93395364857476
C	-2.76864385896105	17.44366446027625	33.50792710900514
C	-4.07649780287214	16.88753439629312	34.06405529174201
C	-5.09505175242640	17.87821356090913	34.57607313798318
O	-4.73350065878891	19.15184867015502	34.49622308084719
O	-6.17076628880879	17.52309029395877	35.03119919305916
H	-7.37580617338489	18.58041550040738	36.03553891882640
H	-2.23512839981904	17.98687760337230	34.29159983166008
H	-2.97923065048090	18.17217516552224	32.72342238578418
H	-4.59423980692710	16.29507983221767	33.30272935013922

H	-3.88288209302088	16.19568346813239	34.88980816095302
H	-2.37132761885948	15.81379318670925	32.12066555930605
H	-1.60429564155913	15.61216252217488	33.69742331926073
H	-0.94834382547963	16.77280904384615	32.53867413069887
C	0.94577436739590	20.56591557326409	28.80344318816237
C	0.63507453525391	21.76241851500751	29.71082531272753
C	-0.65512475281820	21.54512340681551	30.45915471878453
C	-1.86537619162363	21.67206807246341	29.77050357982577
C	-0.70440533843231	21.14292735498893	31.79068174231790
C	-3.07721588121838	21.37775481287195	30.37082777980238
C	-1.91677354941545	20.85986586283343	32.41636653112665
C	-3.10460191930966	20.95881904776771	31.69795878151028
O	-4.32429004955304	20.67641072239507	32.24094490103606
H	1.45411492320655	21.90913479513506	30.41930690495099
H	0.56534099192075	22.67047968892458	29.10604213050870
H	-1.85503048919650	21.99127394901440	28.73432532320640
H	0.21608422822804	21.04220888733230	32.35473055675794
H	-4.00725657136873	21.45845657928491	29.82204470574005
H	-1.94032940149219	20.55086684871639	33.45402758673844
H	-4.23046383941712	20.13431655509879	33.04521118067308
H	0.14619368555658	20.41298831656348	28.07571094744999
H	1.04169714727498	19.65084641797229	29.39246747052177
H	1.87845263139241	20.71428685077247	28.25365273075471
Cu	-5.54440575380378	22.57601082757618	34.18106334332503
O	-7.79179735205985	19.28712912484142	36.56621554630636
O	-6.33964227093468	21.06506664981681	35.05490045563426
H	-7.52502058849075	20.09351981146182	36.08183595073169
H	-5.46270909925122	19.82765479083907	34.78234319253293
